# Safety of midodrine in patients with heart failure with reduced ejection fraction: a retrospective cohort study

**DOI:** 10.3389/fphar.2024.1367790

**Published:** 2024-03-06

**Authors:** Ming-Ju Wu, Cheng-Hsu Chen, Shang-Feng Tsai

**Affiliations:** ^1^ Division of Nephrology, Department of Internal Medicine, Taichung Veterans General Hospital, Taichung, Taiwan; ^2^ Department of Post-Baccalaureate Medicine, College of Medicine, National Chung Hsing University, Taichung, Taiwan; ^3^ Department of Life Science, Tunghai University, Taichung, Taiwan; ^4^ Ph.D. Program in Tissue Engineering and Regenerative Medicine, College of Medicine, National Chung Hsing University, Taichung, Taiwan

**Keywords:** midodrine, heart failure with reduced ejection fraction (HFrEF), mortality, acute pulmonary edema, respiratory failure, admission, safety

## Abstract

**Background:** Heart failure with reduced ejection fraction (HFrEF) poses significant health risks. Midodrine for maintaining blood pressure in HFrEF, requires further safety investigation. This study explores midodrine’s safety in HFrEF through extensive matched analysis.

**Methods:** Patients with HFrEF (LVEF <50%) without malignancy, non-dialysis dependence, or non-orthostatic hypotension, were enrolled between 28 August 2013, and 27 August 2023. Propensity score matching (PSM) created 1:1 matched groups. Outcomes included mortality, stage 4 and 5 chronic kidney disease (CKD), emergency room (ER) visits, intensive care unit (ICU) admissions, hospitalizations, and respiratory failure. Hazard ratios (HR) with 95% confidence intervals (95% CI) were calculated for each outcome, and Kaplan-Meier survival analysis was performed. Subgroup analyses were conducted based on gender, age (20-<65 vs. ≥65), medication refill frequency, and baseline LVEF.

**Results:** After 1:1 PSM, 5813 cases were included in each group. The midodrine group had higher risks of respiratory failure (HR: 1.16, 95% CI: 1.08–1.25), ICU admissions (HR: 1.14, 95% CI: 1.06–1.23), hospitalizations (HR: 1.21, 95% CI: 1.12–1.31), and mortality (HR: 1.090, 95% CI: 1.01–1.17). Interestingly, midodrine use reduced ER visits (HR: 0.77, 95% CI: 0.71–0.83). Similar patterns of lower ER visit risk and higher risks for ICU admissions, respiratory failure, and overall hospitalizations were observed in most subgroups.

**Conclusion:** In this large-scale study, midodrine use was associated with reduced ER visits but increased risks of respiratory failure, prolonged ICU stays, higher hospitalizations, and elevated mortality in HFrEF patients. Further research is needed to clarify midodrine’s role in hemodynamic support and strengthen existing evidence.

## Highlights

• Midodrine has been utilized for treating hypotension, particularly in patients with heart failure, albeit without confirmed safety.
• This large-scale study found that while midodrine use was linked to reduced emergency room visits, it also posed increased risks of respiratory failure, prolonged ICU stays, heightened hospitalizations, and elevated mortality among patients with heart failure with reduced ejection fraction (HFrEF).
• Therefore, considering both its advantages and disadvantages, the long-term usage of midodrine should be carefully assessed and justified.

## Introduction

Heart failure (HF) is a prevalent clinical syndrome stemming from various cardiac diseases, with even treated cases, particularly HF with reduced ejection fraction (HFrEF), often yielding unfavorable outcomes. Numerous factors are entwined with the prognosis of HFrEF, including age (Ho et al., 1993), gender (Levy et al., 2002), race (Dries et al., 2002; Rathore et al., 2003), and the underlying cause of the cardiomyopathy (Felker et al., 2000). The 5-year survival rate hovers around a mere 50% (Loehr et al., 2008; Jones et al., 2019), which was worse than that of many malignancies (Nordio et al., 2012). Recent years have witnessed the emergence of several medications aimed at enhancing patient survival (such as beta blockers, angiotensin-converting-enzyme inhibitors (ACEi), angiotensin II receptor blockers (ARB), angiotensin receptor-neprilysin inhibitors (ARNi), mineralocorticoid receptor antagonists (MRA), and sodium-glucose cotransporter-2 inhibitors (SGLT2i)). This amalgamation is termed guideline-directed medical therapy (GDMT) (Choi et al., 2018). Nonetheless, a subset of patients cannot endure GDMT due to advanced HF-related hypotension, notably the blood pressure (BP)-lowering effects of ACEi, ARB, ARNi, and beta blockers.

Midodrine is a peripheral alpha-1 agonist and anti-hypotensive agent approved by the U.S. Food and Drug Administration for treating orthostatic hypotension (OH) since 1996. Historically, midodrine has been well-tolerated due to its infrequent adverse effects. In a large-scale study, 7.9% of the 3,030 patients who received treatment for up to 15 months reported adverse events (McTavish and Goa, 1989). A review of midodrine usage revealed that the most commonly experienced adverse effects are minor, such as piloerector reactions (55%), gastrointestinal disorders (12.6%), cardiovascular complaints (9.5%), and urinary retention (Vaidyanathan et al., 2007; Nordio et al., 2012). Most of the aforementioned issues can be managed by reducing the dosage of midodrine (McTavish and Goa, 1989). As a result, many clinicians prescribe midodrine off-label for conditions like intradialytic hypotension (IDH), bridge from intravenous vasopressor for patients in the intensive care unit (ICU), and hypotension related to HF.

Based on a systemic review of IDH (Prakash et al., 2004), intermittent usage of midodrine before dialysis might be considered safe. However, the daily usage of midodrine for conditions like hypotension (such as HFrEF) has not been thoroughly studied. A recent matched study (Brunelli et al., 2018) indicated that midodrine is associated with a higher mortality rate (incidence rate ratio: 1.37, 95% confidence interval [CI]:1.15–1.62). For ICU usage, a study demonstrated a shorter ICU stay but a higher risk of 1-year mortality (hazard ratio [HR]: 1.60, 95% CI:1.26–2.04) (Rizvi et al., 2019). Another study involving propensity score matching (PSM) for patients undergoing cardiac surgery revealed that midodrine was linked to higher mortality (13.5% vs. 1.4%, *p* = 0.036) (Tremblay et al., 2020). Consequently, midodrine’s safety level is not as assured as our previous expectations.

The evidence regarding midodrine’s safety for HFrEF is also quite limited. In 2009, a case series involving 10 patients (Zakir et al., 2009) demonstrated that after a 6-month follow-up, a higher percentage of patients were on GDMT (*p* < 0.001). Additionally, their left ventricular ejection fraction (LVEF) improved from 24.0% to 32.2% (*p* < 0.001), and there were reduced hospital admissions (32 vs. 12; *p* = 0.02) and total hospital days (150 vs 58; *p* = 0.02). However, a poster presentation (Scoma et al., 2021) indicated significantly higher all-cause mortality at 6 months following hospitalization in the midodrine group compared to the non-midodrine group (HR:6.7, 95% CI:5.2%–8.5%). There have been some recent case reports showing potential benefits (at least in terms of blood pressure improvement and a higher likelihood of GDMT) (Hajjiah et al., 2022; Shiu et al., 2022). However, these studies were only case series with small sample sizes, lacking matched controls, and limited to short-term follow-ups. Consequently, many clinicians still have concerns about the long-term effectiveness and safety of midodrine in HFrEF (Fernández-Fernández et al., 2022; Sinagra and Fabris, 2022). Moreover, there are concerns regarding alpha-1 agonists (such as Phenylephrine) potentially leading to renal dysfunction, as indicated in previous case reports (Shinomiya et al., 2003; Dean and Reddivari, 2023).

Thus, in this study, we utilized TriNetx—a collaborative network offering matched controls and long-term follow-up—to investigate the safety of midodrine for HFrEF in terms of mortality, admissions, and renal function.

## Materials and methods

### Database of TriNetx

We utilized the extensive and expansive dataset provided by TriNetx’s Global Collaborative Network. This repository consisted of information gathered from 104 prominent healthcare organizations (HCOs). TriNetx is a platform that amalgamates data from Electronic Health Records (EHR) and insurance claims into a unified, longitudinal record. To date, it has amassed a participant pool exceeding 122 million individuals from 15 different nations. Furthermore, TriNetx has contributed over 400 articles to PubMed research. Within this dataset, crucial information is included, encompassing demographic particulars, diagnoses [encoded using the 10th Revision of the International Classification of Diseases, Clinical Modification, ICD-10-CM), procedures (encoded using the 10th Revision of the International Classification of Diseases, Procedure Coding System, ICD-10-PCS, or Current Procedural Terminology, CPT), supplementary procedures (encoded using the Systematized Nomenclature of Medicine Clinical Terms, SNOMED CT], medications (encoded using the Veterans Affairs National Formulary), laboratory tests (encoded using the Logical Observation Identifiers Names and Codes, LOINC), and patterns of healthcare utilization. The dataset originated from various sources, including hospitals, primary-care units, and specialized medical facilities. Serving as the cornerstone of our research data, the TriNetx database serves its role as a global health-collaborative clinical-research platform.

For our study, we collected data spanning a decade (from 28 August 2013, to 27 August 2023), capitalizing on the extensive global collaborative network facilitated by TriNetX.

### Ethics statement

As the data is anonymized, the need for informed consent was deemed unnecessary. It is of utmost importance to emphasize that TriNetX diligently adheres to the principles outlined in the Health Insurance Portability and Accountability Act and the General Data Protection Regulation. Additionally, the Western Institutional Review Board has granted TriNetX the authority to waive the informed consent requirement, given that the platform exclusively aggregates counts and statistical summaries of de-identified data. Moreover, our meticulous utilization of the TriNetX platform within the scope of this study received approval from the institutional review board (IRB) at Taichung Veterans General Hospital (approval number: SE22220A-1).

### Study design

**FIGURE 1:**
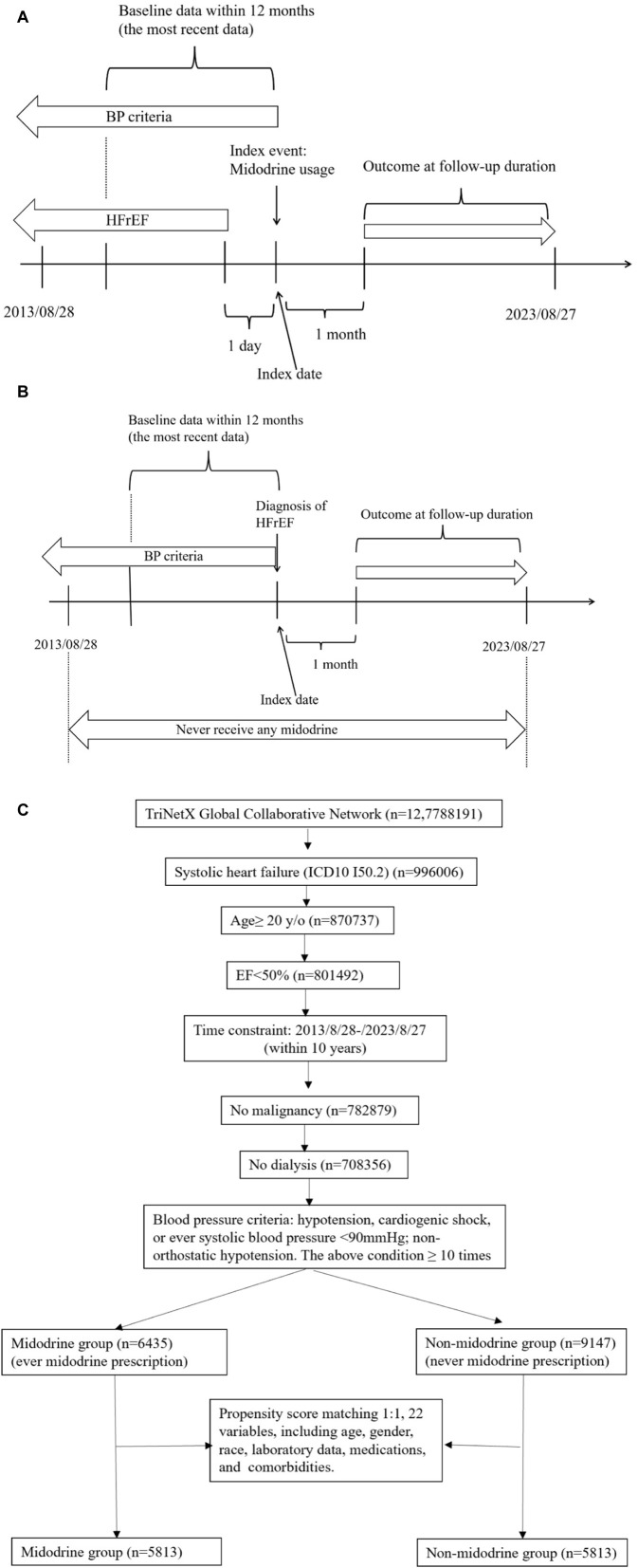
(Continued). Study design [smidodrine **(A)** and non-midodrine **(B)** group], and flowchart of cohort construction **(C)**.

Detailed study designs are presented in Figure 1A for the midodrine group and Figure 1B for the non-midodrine group. For the midodrine group (Figure 1A), the index event is the use of midodrine, and the reference time is the index date. Any diagnosis of HFrEF should precede the index event by at least 1 day. Additionally, included patients should also meet the blood pressure criteria on the same day or before the index date. In the non-midodrine study design (Figure 1B), included patients should not have taken any midodrine during the study period. The index event is the diagnosis of HFrEF, and any blood pressure criteria should be met on the same day or before the index event. The time windows for both groups is the same. The time window for baseline data collection is within 1 year before the index event, and we collect the most recent data available. The time window for the outcome follow-up is at least 1 month after the index event until end of follow-up to avoid reverse causality.

### Definition of population

The study population was defined based on ICD-10-CM code I50.2 for systolic heart failure. Individuals under the age of 20 were excluded in accordance with the regulations set forth by our institute’s IRB. To ensure a cleaner dataset, we also established a criterion that left ventricular ejection fraction (LVEF) should be less than 50% (LOINC Code 10230-1). Additionally, we applied specific BP criteria to identify cases of HFrEF and hypotension. This involved a minimum of ten diagnoses for conditions such as hypotension (ICD-10-CM code I95), cardiogenic shock (ICD-10-CM code R57.0), or a systolic blood pressure (SBP) below 90 mmHg (LOINC Code 8480-6). Cases with a diagnosis of orthostatic hypotension (ICD-10-CM code I95.1) were excluded. Patients with end-stage kidney disease (ESKD) (ICD-10-CM code N18.6) were excluded to prevent the influence of midodrine administration for intradialytic hypotension. Additionally, individuals with malignancy (ICD-10-CM code C80.1) were also excluded. The administration of midodrine was documented in accordance with ATC code C01CA17.

For the purpose of subgroup analysis, we categorized the study population into the following groups: male vs. female, individuals aged 20-65 vs. those aged 65 and above, frequency of prescription (with at least a certain number of medication refills, specifically 1, 3, 6, and 9 times at a minimum), and baseline LVEF categories (40%–50%, 30%–40%, 20%–30%, and <20%).

### Pre-specified outcomes

To prevent reverse causality, the follow-up period commenced 30 days after the test and continued until the end of the study. Outcomes were defined using diagnosis codes derived from inpatient claims and records, which included both primary and secondary diagnoses for a comprehensive assessment. All intended outcomes are pre-specified with the following designs, as indicated by the corresponding codes:

(1) Renal funciotn related: CKD stage 4 (ICD-10-CM N18.4), and CKD stage 5 (ICD-10-CM N18.5).
(2) Respiraotry conditon related: acute pulmonary edema (ICD-10-CM J81.0), and respiratory failure (ICD-10-CM J96, ICD-10-PCS 5A19054, 5A1935Z, 5A1945Z, 5A1955Z, 31500, 0BH18EZ, 0BH17EZ, 0BH13EZ, CPT: 94002, 94003, 94004, 94005, 1015098, 31500, 1022227, or 1015198).
(3) Admission related: staty intensive care unit (ICU) (CPT: 99291, 99292, 94002, 94003, 94004, 94005, 33946, 33947, 33948, 33949, 33951,33952, 33953, 33954, 33955, 33956, 33957, 33958, 33959, 33962, 33963, 33964, 33965, 33966, 33969, 33984, 33985, 33986, 33987, 33988, or 33989), emergency room (ER) visit (CPT: 1013711, 99281, 99282, 99283, 99284, or 99285), and all hospitlization (CPT: 1013659, 1013660, 1013699, 1013729, 99221, 99222, 99223,99231, 99232, 99233, 99234, 99235, 99236, 99238, 99239,99251, 99252, 99253, 99254, or 99255).
(4) Others: cardiac arrest (ICD-10-CM I46) and all-cause mortality (ICD10 = R99).

### Statistical analyses

In this research, we employed propensity score matching (PSM) on the TriNetX platform. This approach generated matched groups in a 1:1 ratio, ensuring they had comparable baseline characteristics through a greedy nearest neighbor matching technique, with a caliper set at 0.1 times the pooled standard deviations (SDs). The variables used for the matching process encompassed a range of factors, including demographic particulars (age at index, gender, and race), underlying medical conditions (such as diabetes mellitus, ischemic heart disease, and chronic obstructive pulmonary disease), medications (including ACE inhibitors, angiotensin II inhibitors (including angiotensin receptor-neprilysin inhibitor), beta blockers, spironolactone, eplerenone, empagliflozin, dapagliflozin, canagliflozin, and ivabradine), blood-related laboratory data (glomerular filtration rate in ml/min/1.732 m^2^ and N-terminal pro–B-type natriuretic peptide (NT-proBNP) in pg/mL), LVEF (%), as well as SBP (mmHg).

Continuous variables were reported as mean ± SD, while categorical variables were presented as n (%). To assess the comparability of baseline characteristics within the matched groups established by propensity scores, the standardized mean difference (SMD) was utilized. A SMD value <0.1 indicates a minor disparity, signifying successful matching.

Following that, hazard ratios (HRs) for all outcomes were computed for both the midodrine and non-midodrine groups. The assumption of proportional hazards was evaluated using the generalized Schoenfeld approach, conveniently integrated into the TriNetX platform. If the HR is statistically significant, the Kaplan-Meier survival curve will be plotted. Censoring was applied for patients who exited the cohort during the analysis period and should not be included. Throughout all analyses, statistical significance was defined as a confidence level of 95% (95% CI).

## Results

### Patient selection algorithms

The patient selection algorithm is summarized in Figure 1C. We enrolled patients with HFrEF (LVEF <50%), aged ≥20 years old, within the recent 10 years (from 2013/08/28 to 2023/08/27), without malignancy or ESKD. To enhance PSM, we applied BP criteria (fit at least 10 times: hypotension, cardiogenic shock, or ever systolic blood pressure <90 mmHg; non-orthostatic hypotension) to select advanced HFrEF patients. Ultimately, we included patients who had taken midodrine (*n* = 6435) and those who had never taken midodrine (*n* = 9147). Subsequently, they underwent 1:1 PSM using 22 variables (as shown in Table 1) that are associated with mortality in patients with HFrEF. In each group, we had a total of 5813 patients available for further analysis.

**TABLE 1:** Baseline characteristics of study subjects (before and after propensity score matching).

	Before matching				After matching			
Characteristic Name	Midodrine group (n = 6435)	Non-midodrine group (n = 9174)	*p*-value	SMD	Midodrine group (n = 5813)	Non-midodrine group (n = 5813)	*p*-value	SMD
Demographic data								
Age at Index (y/o)	65.0 ± 13.1	61.3 ± 13.9	<0.001	0.2757	64.1 ± 13.2	64.2 ± 12.9	0.9496	0.0012
Male	4085(63.48%)	5998(65.38%)	0.0146	0.0397	3718(63.96%)	3738(64.30%)	0.6990	0.0072
Race								
White	4294(66.73%)	5342(58.23%)	<0.001	0.1762	3737(64.29%)	3766(64.79%)	0.5740	0.0104
Black or African American	1210(18.80%)	2348(25.59%)	<0.001	0.1639	1186(20.40%)	1167(20.08%)	0.6610	0.0081
Asian	147(2.28%)	164(1.79%)	0.0288	0.0352	130(2.24%)	120(2.06%)	0.5226	0.0119
Vital sign								
Blood Pressure, Systolic (mmHg)	88.9 ± 21.5	89.8 ± 20.2	0.0137	0.0424	88.8 ± 21.4	90.4 ± 20.4	<0.001	0.0778
Comorbidity								
Ischemic heart diseases	4771(74.14%)	6502(70.87%)	<0.001	0.0732	4240(72.94%)	4255(73.20%)	0.7538	0.0058
Cardiomyopathy	3307(51.39%)	5685(61.97%)	<0.001	0.2147	3161(54.38%)	3138(53.98%)	0.6686	0.0079
DM	3075(47.79%)	4043(44.07%)	<0.001	0.0746	2716(46.72%)	2712(46.65%)	0.9407	0.0014
COPD	2071(32.18%)	2558(27.88%)	<0.001	0.0939	1799(30.95%)	1824(31.38%)	0.6166	0.0093
Laboratory data of blood								
Glomerular filtration rate (MDRD) (ml/min/1.732m^2^)	59.15 ± 40.84	58.32 ± 29.25	0.1545	0.0234	59.34 ± 40.85	57.23 ± 28.91	0.0020′	0.0594
NT-proBNP (pg/mL)	9872.8 ± 11977.7	8308.2 ± 10699.1	<0.001	0.1378	9812.3 ± 11915.1	8708.1 ± 11221.9	0.0056	0.0954
LVEF (%)	27.5 ± 11.0	25.7 ± 11.4	0.0005	0.1556	27.4 ± 11.0	27.1 ± 11.5	0.5724	0.0295
Medication								
ACE inhibitor	2089(32.46%)	3944(42.99%)	<0.001	0.2185	2026(34.85%)	1970(33.89%)	0.2742	0.0203
Angiotensin II inhibitor (including angiotensin receptor/neprilysin inhibitor)	2104(32.70%)	3297(35.94%)	<0.001	0.0683	1923(33.08%)	1955(33.63%)	0.5290	0.0117
Beta blockers	5184(80.56%)	7383(80.48%)	0.8987	0.0021	4649(79.98%)	4618(79.44%)	0.4747	0.0133
Spironolactone	2556(39.72%)	4335(47.25%)	<0.001	0.1524	2414(41.53%)	2431(41.82%)	0.7491	0.0059
Eplerenone	142(2.21%)	324(3.53%)	<0.001	0.0794	140(2.41%)	148(2.55%)	0.6331	0.0089
Empagliflozin	480(7.46%)	695(7.58%)	0.7859	0.0044	439(7.55%)	434(7.47%)	0.8603	0.0033
Dapagliflozin	365(5.67%)	538(5.86%)	0.6125	0.0080	325(5.59%)	327(5.63%)	0.9357	0.0015
Canagliflozin	28(0.44%)	45(0.49%)	0.6175	0.0082	25(0.43%)	23(0.40%	0.7724	0.0054
Ivabradine	130(2.02%)	200(2.18%)	0.4943	0.0111	122(2.10%)	117(2.01%)	0.7438	0.0060

DM, diabetes mellitus; COPD, chronic obstructive pulmonary disease; MDRD, modification of diet in renal disease; NT-proBNP, N-terminal pro b-type natriuretic peptide; LVEF, left ventricular ejection fraction; ACE, inhibitor Angiotensin-converting enzyme; SDM: standardized mean difference.

### Baseline characteristics of this cohort before and after matching

In Table 1, prior to PSM, the midodrine group exhibited significant differences (*p* < 0.05), with the following characteristics: they were notably older, had fewer males, a higher proportion of white individuals, fewer black or African American individuals, more Asians, lower SBP, a higher prevalence of ischemic heart disease, a lower prevalence of cardiomyopathy, a higher prevalence of diabetes mellitus and chronic obstructive pulmonary disease, elevated NT-proBNP levels, higher LVEF, lower prescription rates of ACEi, ARBs, spironolactone, and eplerenone. However, after performing 1:1 PSM, there were no statistically significant differences between the two groups (Supplementary Figure S1), as indicated by all SMDs being less than 0.1. The median duration of follow-up for the midodrine group was 10.3 months, while it was 11.5 months for the non-midodrine group. In terms of loss of follow-up, there were 329 patients (5.6%) in the midodrine group and 266 patients (4.6%) in the non-midodrine group who were censored.

### Incidence of outcomes between midodrine and non-midodrine group

**FIGURE 2:**
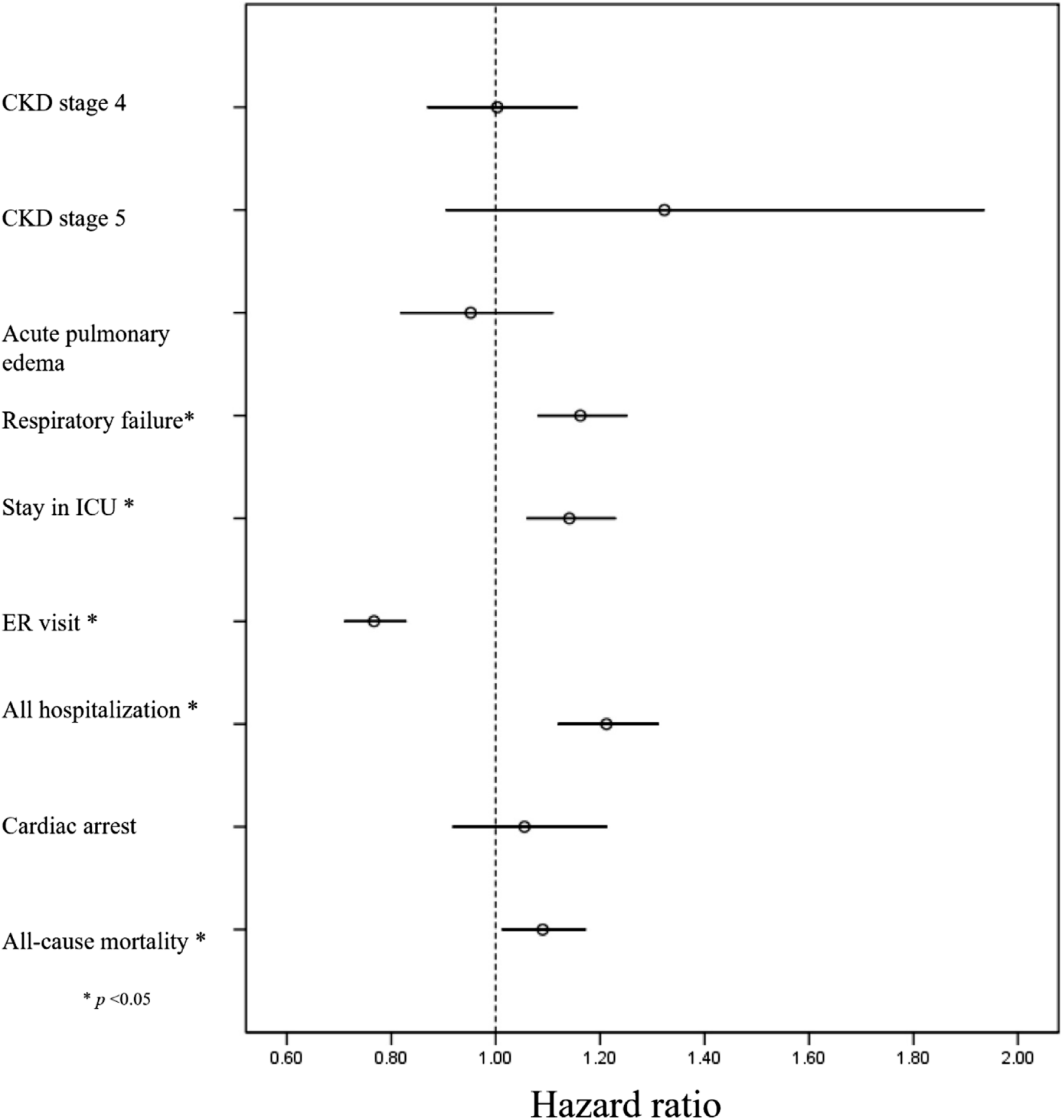
Foret plot of all outcomes in midodrine and non-midodrine groups.

The incidence of all pre-specified outcomes is presented in Table 2 (following PSM), along with Supplementary Figure S1 (prior to PSM). The midodrine group exhibited a significantly higher risk of various outcomes: respiratory failure (HR: 1.16, 95%CI: 1.08–1.25), ICU admission (HR: 1.14, 95% CI: 1.06–1.23), hospitalizations (HR: 1.21, 95%CI: 1.12–1.31), and all-cause mortality (HR: 1.090, 95%CI: 1.01–1.17). However, midodrine was associated with a lower frequency of ER visits (HR: 0.77, 95%CI: 0.71–0.83). The forest plot depicting all outcomes can be found in Figure 2.

**TABLE 2:** Incidence of outcomes among midodrine and non-midodrine group (after prosperity score matching).

Outcome	Patients with outcome		Hazard ratio (95% with CI)	*p*-value
	Midodrine	Non-midodrine		
CKD, stage 4	402	401	1.003 (0.869, 1.157)	0.971
CKD, stage 5	62	47	1.323 (0.904, 1.936)	0.149
Acute pulmonary edema	340	356	0.952 (0.817, 1.110)	0.532
Respiratory failure	2506	2294	1.162 (1.080, 1.252)	<0.001
Stay in intensive care unit	2295	2114	1.141 (1.059, 1.230)	0.001
Emergency room visit	1768	2110	0.767 (0.710, 0.829)	<0.001
All hospitalization	4190	3956	1.212 (1.119, 1.312)	<0.001
Cardiac arrest	436	415	1.055 (0.917, 1.213)	0.455
All-cause mortality	2470	2349	1.090 (1.012, 1.173)	0.023

CKD: chronic kidney disease.

**FIGURE 3:**
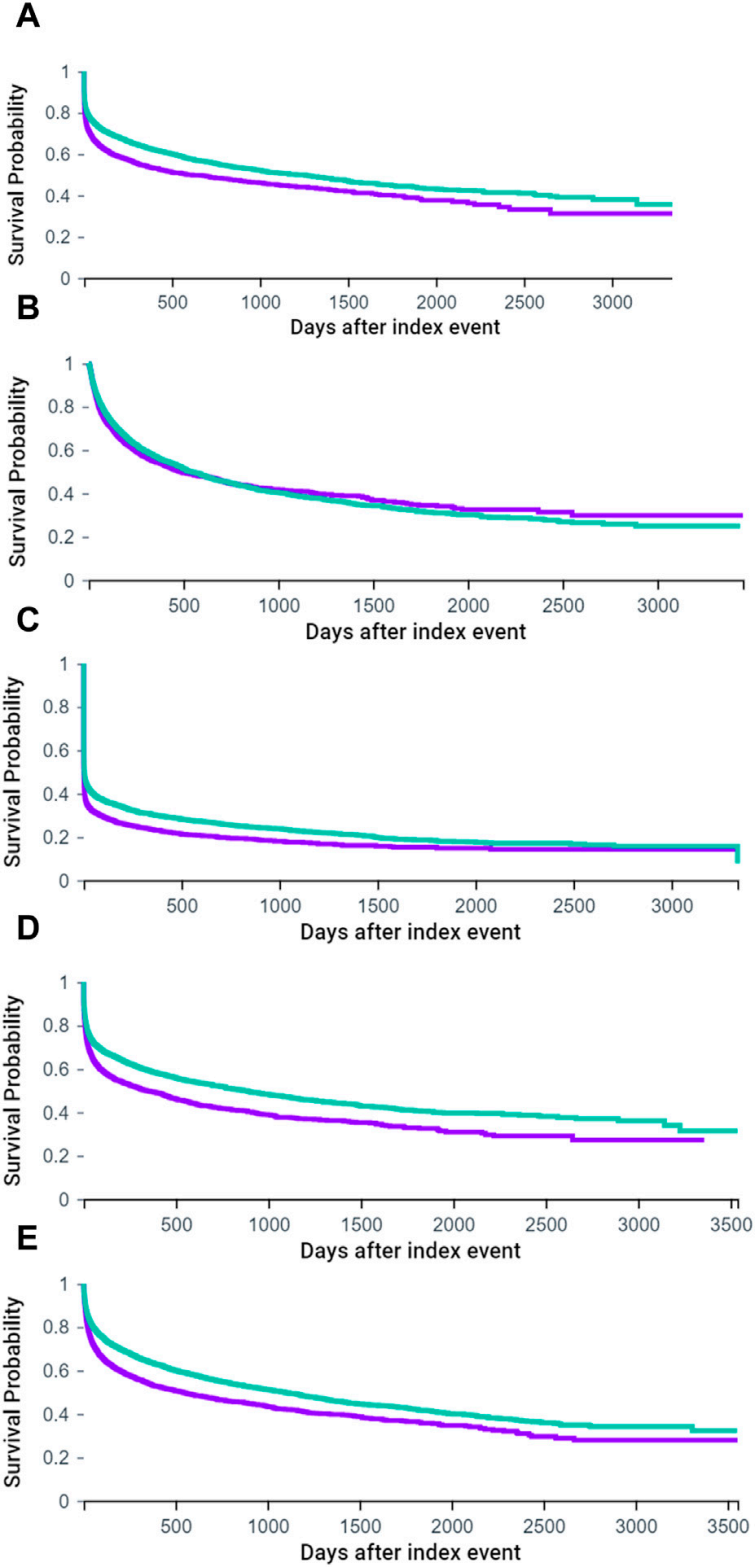
Kaplan-Meier curves of ICU stay **(A)**, ER visit **(B)**, all hospitalization **(C)**, respiratory failure **(D)** and all-cause mortality **(E)**. (purple: midodrine group, green: non-midodrine group) **(A)**. ICU stay (log-rank *p* < 0.001).

In terms of Kaplan-Meier curves (Figure 3), the midodrine group exhibited reduced survival rates across various outcomes, including ICU stays (Figure 3A), all hospitalizations (Figure 3C), respiratory failure (Figure 3D), and all-cause mortality (Figure 3E). Regarding ER visits (Figure 3B), the midodrine group initially exhibited a probability of ER visits that was similar to or higher than the comparison group. However, the probability of ER visits for this group may have decreased later on (without statistical significance, log-rank *p* = 0.1672).

### Subgroup analyses conducted based on gender, age, frequency of medication prescription, and baseline LVEF

Firstly, in the subgroup analysis of male and female midodrine groups (Supplementary Figure S2A for males, 2B for females, and Supplementary Table S14), both exhibited significantly higher risks for respiratory failure, ICU stay, all hospitalizations, and fewer ER visits. The female midodrine group also demonstrated a higher risk of cardiac arrest. The baseline characteristics, both before and after PSM, for the male and female groups were presented in Supplementary Table S2, S3, respectively.

Next, in the subgroup analysis of young and old midodrine groups (Supplementary Figure S3A for young, 3B for old, and Supplementary Table S14), the young midodrine group had a greater risk of respiratory failure and all-cause mortality, along with a lower risk of ER visits. Conversely, the older midodrine group exhibited higher risks of stage 5-CKD, acute pulmonary edema, respiratory failure, ICU stay, and all hospitalizations. The baseline characteristics, before and after PSM, for the young and old groups were shown in Supplementary Table S4, S5, respectively.

In the analysis of medication refill times (Supplementary Figure S4A for ≥3, 4B for ≥6, and 4C for ≥9 times; Supplementary Table S15), across all conditions, the midodrine group had a higher risk of respiratory failure, ICU stay, and all hospitalizations. As the number of refill times increased, the midodrine group’s risk heightened for stage 4-CKD, acute pulmonary edema, respiratory failure, ICU stay, ER visits, all hospitalizations, cardiac arrest, and all-cause mortality. Baseline characteristics for groups with different refill times were displayed in Supplementary Table S6 (refill time ≥3), Supplementary Table S7 (refill time ≥6), and Supplementary Table S8 (refill time ≥9), respectively.

Concerning baseline LVEF (Supplementary Figure S5A for LVEF = 40–50%, 5B for 30%–40%, 5C for 20%–30%, and 5D for <20%; Supplementary Table S16), across all baseline LVEF levels, the midodrine group exhibited a higher risk of respiratory failure (except for LVEF< 20%), ICU stay, and all hospitalizations. For all baseline LVEF levels (except LVEF = 40–50%), the midodrine group had a lower risk of ER visits. Baseline characteristics for groups with different LVEF levels were outlined in Supplementary Table S10 (LVEF = 40–50%), Supplementary Table S11 (LVEF = 30–40%), Supplementary Table S12 (LVEF = 20–30%), and Supplementary Table S13 (LVEF< 20%).

For all the aforementioned subgroups, due to limited data, complete matching for all variables was not achievable. The incidence of outcomes in all conditions was listed in Supplementary Table S17.

## Discussion

This is the first large-scale matched cohort study investigating the safety of midodrine in patients with systolic heart failure. To date, there have been no studies investigating the impact of midodrine usage on ER visits, ICU stays, and all-cause hospitalizations in patients with systolic heart failure. Based on our results, midodrine group had lower risk for ER visit, but higher risk for respiratory failure, ICU stay, all hospitalization and all-cause mortality. Especially, lower risk for ER visit, higher risk for ICU stay, respiratory failure, and all hospitalization in midodrine were noted across nearly all subgroups.

Firstly, in terms of hospitalization-related outcomes, the midodrine group exhibited fewer ER visits but more ICU stays, respiratory failures, all hospitalizations, and mortality. Until now, only case reports (Hajjiah et al., 2022) or case series (Zakir et al., 2009) have addressed midodrine usage in heart failure. In one case series (*n* = 10) (Zakir et al., 2009), midodrine usage was associated with fewer total hospital admissions and total hospital days. However, they did not differentiate between ER visits or ICU stays in their study. Additionally, in their patient cohort, midodrine was used to achieve a higher rate of GDMT. In our study, the primary use of midodrine among patients was to maintain hemodynamic stability rather than to achieve a higher rate of GDMT. Particularly noteworthy, our study is the first to differentiate between ER visits and ICU stays for hospitalizations in this context.

The majority of hospitalizations for HF typically originate in the ER (Schuur and Venkatesh, 2012). ER visits are both common and vital for patients with HFrEF (Martindale et al., 2016; Miró et al., 2017). Moreover, many patients can be adequately treated and discharged directly from the ER (Weintraub et al., 2010; Hasin et al., 2018). Consequently, a substantial number of HF patients may experience more ER visits while encountering fewer hospitalizations. Nevertheless, based on our findings, a different narrative may emerge when midodrine is introduced. Patients with HFrEF administered midodrine to achieve improved BP levels. This enhanced SBP could potentially mislead caregivers and physicians into erroneously perceiving stability in HFrEF, thereby leading to delays in diagnosis and treatment. The mistaken impression of hemodynamic stability may prevent patients from recognizing episodes of hypotension, subsequently prompting visits to the ER. This delay in detection and treatment, in turn, contributes to an elevated occurrence of hospitalizations, respiratory failures, prolonged stays in the ICU, and increased mortality rates. The 2017 American College of Cardiology Expert Consensus introduced the mnemonic “I-NEED-HELP” as a tool for rapidly identifying high-risk patients (Yancy et al., 2018). When patients use midodrine to sustain their SBP, they could overlook the “L” criterion (low SBP≤ 90 mmHg) and potentially experience worsened final outcomes. In light of these considerations, we recommend that all patients receiving midodrine undergo similar early referrals to cardiologists. This approach aims to ensure comprehensive and proactive management, thereby mitigating the risk of adverse events. Furthermore, the escalating refill frequency of midodrine serves as an early indicator of deteriorating heart failure, prompting individuals to promptly consult a cardiologist. This phenomenon is discernible in our dataset (Supplementary Table S15), wherein a rise in refill frequency correlates with further elevated HRs for outcomes such as respiratory failure, extended ICU stays, overall hospitalizations, and mortality.

Indeed, it is intriguing that the HR for ER visit exhibits a corresponding increase in conjunction with refill frequency: transitioning from lower (refill time≥1) to neutral (≥3 and ≥6) and then to higher (≥9) risk levels. Initially, the risk of ER visits appears to be diminished in refill frequency is ≥ 1, a trend that could be attributed to delayed detection. However, the risk profile becomes neutral as refill frequencies reach ≥3 and ≥6. This shift might be attributed to the fact that much higher refill frequencies potentially serve as an alert to both patients and caregivers regarding the worsening state of heart failure. Ultimately, when the refill frequency reaches ≥9, even the utilization of midodrine fails to mask the advanced stage of HFrEF, leading to a surge in ER visits when compared to the non-midodrine cohort. Therefore, when it comes to delayed diagnosis, special attention should be given to cases involving even low frequencies of midodrine refills. In this study, we emphasized the importance of not overlooking patients who are taking midodrine. This medication has the potential to conceal hypotension from patients, their families, and physicians. By the time patients require midodrine, they may already be experiencing unstable hemodynamic status. It is imperative that we inform patients and their families about this aspect and educate them on when to seek further evaluation in the emergency room or cardiovascular department (such as with higher doses or more frequent use of midodrine). We believe that clinicians should be mindful of this consideration when prescribing midodrine to patients.

Apart from delayed detection of deteriorating heart function, there may be other reasons contributing to the elevated mortality associated with midodrine usage. Firstly, excessive midodrine consumption can potentially lead to severe hypertension and reflex bradycardia (Wong et al., 2017). A study involving ICU patients highlighted that bradycardia was the most prevalent adverse effect (15% for heart rate <50/min and 9% for heart rate <40/min) (Rizvi et al., 2018). This effect might be more pronounced in HFrEF patients concurrently taking beta blockers. Secondly, midodrine has the potential to induce peripheral vasoconstriction, leading to ischemic events (Rubinstein et al., 2008). This scenario bears resemblance to mortality related to phenylephrine-induced vasoconstriction (Vail et al., 2017). Thirdly, the use of midodrine has been linked to the development of supine hypertension (SH). When midodrine is administered, the likelihood of SH increases (relative risk: 5.1, 95%CI:1.6–24), particularly at doses exceeding 20 mg/day (Olshansky and Muldowney, 2020). SH has demonstrated superior predictive value for all-cause mortality and cardiovascular mortality compared to blood pressure measured in other body postures (Baik et al., 2023). Furthermore, SH has been independently associated with an earlier onset of cardiovascular events and death (Palma et al., 2020).

There are certain limitations to this study. Firstly, it is an observational and retrospective study. However, conducting randomized controlled trials in patients with advanced HFrEF is nearly impossible. Secondly, we cannot entirely rule out the presence of unknown residual confounders. To mitigate this potential bias, we employed PSM to align as many relevant heart-related factors as possible. Thirdly, we did not differentiate between inpatients and outpatients when administering midodrine, as our intention was to capture a comprehensive clinical overview of midodrine’s usage. Additionally, healthcare providers typically transition patients from intravenous vasopressors to oral midodrine when they are in a more stable condition. This transition usually occurs a few days before discharge for inpatients. Consequently, we believed that this limitation would have a minimal impact. Fourthly, it cannot be conclusively determined that all hospitalization-related outcomes are solely attributed to HFrEF. However, HF stands as one of the leading causes of readmission (Jencks et al., 2009; Blecker et al., 2013). Fifthly, we did not include all patients with heart failure in this study. Moreover, the majority of patients in this study (>99%) originate from United States regions, with less than 1% of patients coming from ex-United States regions. In the future, we plan to conduct similar studies using databases from regions other than the United States. Despite these limitations, we maintain confidence in the robustness of our study to examine the impact of midodrine on patient safety.

## Conclusion

This extensive matched study demonstrated an association between midodrine usage and a reduced frequency of ER visits, yet an elevated occurrence of respiratory failure, extended ICU stays, increased overall hospitalizations, and higher mortality rates in HFrEF patients. Logically, higher refill frequencies may indicate a more advanced condition, necessitating consultation with a cardiologist. Greater emphasis should be placed on monitoring lower refill frequencies to prevent delayed detection of deteriorating heart function. The use of midodrine for hemodynamic support for GDMT warrants further research to strengthen the available evidence.

## Data Availability

The raw data supporting the conclusion of this article will be made available by the authors, without undue reservation.
