# Supplementary material for: Safety of midodrine in patients with heart failure with reduced ejection fraction: a retrospective cohort study
Source: Front Pharmacol. 2024 Mar 6;15:1367790. doi: 10.3389/fphar.2024.1367790 (PMC10953504; doi:10.3389/fphar.2024.1367790)
Supplement: Supplementary file 1 [file DataSheet1.PDF]

Supplementary data

- Table 1. Incidence of outcomes among midodrine and non-midodrine group (before prosperity score matching)
- Table 2. Baseline characteristics of study subjects in male gender (before and after propensity score matching). (cohort 1, purple, midodrine group; cohort 2, green: non-midodrine group)
- Table 3. Baseline characteristics of study subjects in female gender (before and after Propensity score matching). (cohort 1, purple, midodrine group; cohort 2, green: non-midodrine group)
- Table 4. Baseline characteristics of study subjects in young age (18-<65 y/o) (before and after Propensity score matching). (cohort 1, purple, midodrine group; cohort 2, green: non-midodrine group)
- Table 5. Baseline characteristics of study subjects in old age (≥65 y/o) (before and after Propensity score matching). (cohort 1, purple, midodrine group; cohort 2, green: non-midodrine group)
- Table 6. Baseline characteristics of study subjects in frequency of medication (at least 3 times of medication refills) (before and after Propensity score matching). (cohort 1, purple, midodrine group; cohort 2, green: non-midodrine group)
- Table 7. Baseline characteristics of study subjects in frequency of medication (at least 6 times of medication refills) (before and after Propensity score matching). (cohort 1, purple, midodrine group; cohort 2, green: non-midodrine group)
- Table 8. Baseline characteristics of study subjects in frequency of medication (at least 9 times of medication refills) (before and after Propensity score matching). (cohort 1, purple, midodrine group; cohort 2, green: non-midodrine group)
- Table 9. Baseline characteristics of study subjects in baseline LVEF=40-50% (before and after Propensity score matching). (cohort 1, purple, midodrine group; cohort 2,

green: non-midodrine group)

- Table 10. Baseline characteristics of study subjects in baseline LVEF=30-40% (before and after Propensity score matching). (cohort 1, purple, midodrine group; cohort 2, green: non-midodrine group)
- Table 11. Baseline characteristics of study subjects in baseline LVEF=20-30% (before and after Propensity score matching). (cohort 1, purple, midodrine group; cohort 2, green: non-midodrine group)
- Table 12. Baseline characteristics of study subjects in baseline LVEF<20% (before and after Propensity score matching). (cohort 1, purple, midodrine group; cohort 2, green: non-midodrine group)
- Table 13. Incidence of outcomes among midodrine and non-midodrine groups (after prosperity score matching) stratified by genders.
- Table 14. Incidence of outcomes among midodrine and non-midodrine (after prosperity score matching) stratified by age (18-65y/o vs. ≥65y/o)
- Table 15. Incidence of outcomes among midodrine and non-midodrine (after prosperity score matching) stratified by frequency of medication (at least a certain number of medication refills)
- Table 16. Incidence of outcomes among midodrine and non-midodrine (after prosperity score matching) stratified baseline LVEF
- Figure 1. Propensity score density function – before (A) and after (B) matching (cohort 1 -midodrine group (purple), cohort 2 -non-midodrine group (green))
- Figure 2. Forest plot of all outcomes in midodrine and non-midodrine groups in male (A) and female (B).
- Figure 3. Forest plot of all outcomes in midodrine and non-midodrine groups in young (18-<65 y/o) (A) and elderly group (≥65 y/o) (B).
- Figure 4. Forest plot of all outcomes in midodrine and non-midodrine groups in different groups according to times of prescription (at least a certain number of medication refills): ≥3 (A), ≥6 (B), ≥9 (C), and ≥12 (D)

- Figure 5. Forest plot of all outcomes in midodrine and non-midodrine groups in different groups according to baseline LVEF: 40-50% (A), 30-40%6 (B),20-30% (C), and <20% (D)

Table 1. Incidence of outcomes among midodrine and non-midodrine group (before prosperity score matching)

Outcome	Patients with outcome		Hazard ratio (95% with CI)	*p* value
	Midodrine	Non-midodrine		
CKD, stage 4	433	650	0.944 (0.833, 1.071)	0.373
CKD, stage 5	66	82	1.147 (0.828, 1.589)	0.409
Acute pulmonary edema	355	640	0.777 (0.680, 0.888)	<0.001
Respiratory failure	2764	3626	1.149 (1.077, 1.226)	<0.001
Stay in intensive care unit	2523	3548	1.020 (0.955, 1.089)	0.554
Emergency room visit	1933	3617	0.658 (0.615, 0.704)	<0.001
All hospitalization	4659	6412	1.124 (1.047, 1.207)	0.001
Cardiac arrest	477	704	0.961 (0.852, 1.085)	0.524
All-cause mortality	2746	3658	1.119 (1.049, 1.194)	0.001

Table 2. Baseline characteristics of study subjects in male gender (before and after propensity score matching). (cohort 1, purple, midodrine group; cohort 2, green: non-midodrine group)

Cohort 1 and cohort 2 patient count before and after propensity score matching

Cohort	Patient count before matching	Patient count after matching
1 - M+, Male	4,085	3,720
2 - M-, male	5,998	3,720

Propensity score density function - Before and after matching (cohort 1 - purple, cohort 2 - green)

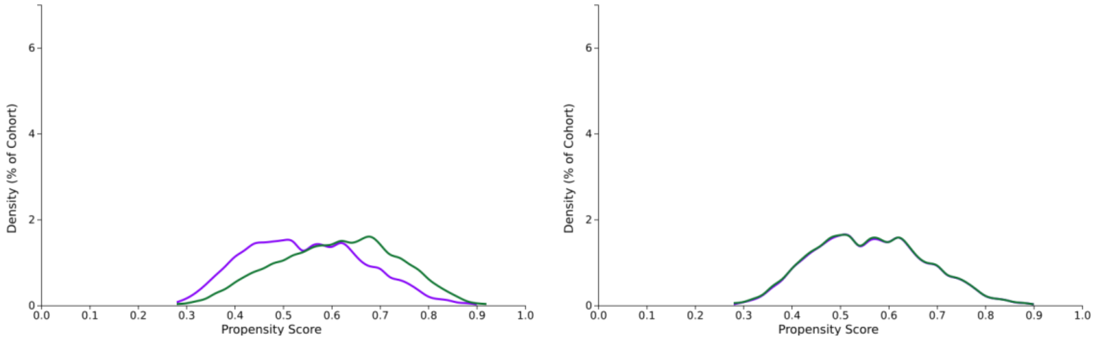

Cohort 1 (N = 4,085) and cohort 2 (N = 5,998) characteristics before propensity score matching

Demographics

Cohort			Mean ± SD	Patients	% of Cohort	P-Value	Std diff.
1	AI	Age at Index	65.7 +/- 12.7	4,085	100%	<0.001	0.314
2			61.6 +/- 13.4	5,998	100%		
1	2106-3	White		2,789	68.3%	<0.001	0.175
2				3,594	59.9%		
1	2054-5	Black or African American		656	16.1%	<0.001	0.182
2				1,396	23.3%		
1	M	Male		4,085	100%	--	--
2				5,998	100%		
1	2028-9	Asian		105	2.6%	0.057	0.038
2				120	2.0%		

Diagnosis

Cohort			Mean ± SD	Patients	% of Cohort	P-Value	Std diff.
1	I20-I25	Ischemic heart diseases		3,191	78.1%	<0.001	0.090
2				4,456	74.3%		
1	E08-E13	Diabetes mellitus		1,976	48.4%	<0.001	0.078
2				2,667	44.5%		
1	I42	Cardiomyopathy		2,181	53.4%	<0.001	0.208
2				3,813	63.6%		

Cohort			Mean ± SD	Patients	% of Cohort	P-Value	Std diff.
1<br>2	J44	Other chronic obstructive pulmonary disease		1,282<br>1,582	31.4%<br>26.4%	<0.001	0.111

Medication

Cohort			Mean ± SD	Patients	% of Cohort	P-Value	Std diff.
1<br>2	CV100	BETA BLOCKERS/RELATED		3,324<br>4,854	81.4%<br>80.9%	0.576	0.011
1<br>2	1649480	ivabradine		82<br>134	2.0%<br>2.2%	0.440	0.016
1<br>2	CV805	ANGIOTENSIN II INHIBITOR		1,412<br>2,209	34.6%<br>36.8%	0.020	0.047
1<br>2	CV800	ACE INHIBITORS		1,385<br>2,607	33.9%<br>43.5%	<0.001	0.197
1<br>2	1545653	empagliflozin		344<br>482	8.4%<br>8.0%	0.489	0.014
1<br>2	1488564	dapagliflozin		247<br>356	6.0%<br>5.9%	0.817	0.005
1<br>2	1373458	canagliflozin		15<br>33	0.4%<br>0.6%	0.190	0.027
1<br>2	9997	spironolactone		1,665<br>2,812	40.8%<br>46.9%	<0.001	0.124
1<br>2	298869	eplerenone		123<br>288	3.0%<br>4.8%	<0.001	0.093

Laboratory

Cohort			Mean ± SD	Patients	% of Cohort	P-Value	Std diff.
1<br>2	9072	Natriuretic peptide.B prohormone N-Terminal [Mass/volume] in Serum, Plasma or Blood	9748.7 +/- 11349.4<br>7905.0 +/- 9960.0	1,110<br>2,071	27.2%<br>34.5%	<0.001	0.173
1<br>2		0 - 0 pg/mL		1,110<br>2,071	27.2%<br>34.5%	<0.001	0.160
1<br>2	2003	Left Ventricular Ejection Fraction (LVEF) (%)	26.8 +/- 10.7<br>24.8 +/- 11.0	516<br>1,177	12.6%<br>19.6%	<0.001	0.186
1<br>2		0 - 0 %		516<br>1,177	12.6%<br>19.6%	<0.001	0.191
1	9085	Blood Pressure, Systolic	88.9 +/- 21.2	3,677	90.0%	0.369	0.019

Cohort			Mean ± SD	Patients	% of Cohort	P-Value	Std diff.
2			89.3 +/- 20.1	5,127	85.5%		
1<br>2		0 - 0 mm[Hg]		3,677<br>5,127	90.0%<br>85.5%	<0.001	0.139
1<br>2	8001	Glomerular filtration rate/1.73 sq M.predicted [Volume Rate/Area] in Serum, Plasma or Blood by Creatinine-based formula (MDRD)	59.6 +/- 40.0<br>59.1 +/- 28.9	3,835<br>5,443	93.9%<br>90.7%	0.453	0.015
1<br>2		0 - 0 mL/min/{1.73_m2}		3,845<br>5,445	94.1%<br>90.8%	<0.001	0.127
**Cohort 1 (N = 3,720) and cohort 2 (N = 3,720) characteristics after propensity score matching**							
**Demographics**							
Cohort			Mean ± SD	Patients	% of Cohort	P-Value	Std diff.
1<br>2	AI	Age at Index	64.7 +/- 12.7<br>64.6 +/- 12.3	3,720<br>3,720	100%<br>100%	0.645	0.011
1<br>2	2106-3	White		2,480<br>2,473	66.7%<br>66.5%	0.863	0.004
1<br>2	2054-5	Black or African American		645<br>635	17.3%<br>17.1%	0.759	0.007
1<br>2	M	Male		3,720<br>3,720	100%<br>100%	--	--
1<br>2	2028-9	Asian		84<br>92	2.3%<br>2.5%	0.542	0.014
**Diagnosis**							
Cohort			Mean ± SD	Patients	% of Cohort	P-Value	Std diff.
1<br>2	I20-I25	Ischemic heart diseases		2,861<br>2,862	76.9%<br>76.9%	0.978	0.001
1<br>2	E08-E13	Diabetes mellitus		1,762<br>1,769	47.4%<br>47.6%	0.871	0.004
1<br>2	I42	Cardiomyopathy		2,090<br>2,100	56.2%<br>56.5%	0.815	0.005
1<br>2	J44	Other chronic obstructive pulmonary disease		1,116<br>1,097	30%<br>29.5%	0.630	0.011
**Medication**							

Cohort			Mean ± SD	Patients	% of Cohort	P-Value	Std diff.
1<br>2	CV100	BETA BLOCKERS/RELATED		3,006<br>3,005	80.8%<br>80.8%	0.977	0.001
1<br>2	1649480	ivabradine		76<br>73	2.0%<br>2.0%	0.804	0.006
1<br>2	CV805	ANGIOTENSIN II INHIBITOR		1,309<br>1,346	35.2%<br>36.2%	0.371	0.021
1<br>2	CV800	ACE INHIBITORS		1,346<br>1,324	36.2%<br>35.6%	0.595	0.012
1<br>2	1545653	empagliflozin		319<br>318	8.6%<br>8.5%	0.967	0.001
1<br>2	1488564	dapagliflozin		221<br>220	5.9%<br>5.9%	0.961	0.001
1<br>2	1373458	canagliflozin		14<br>15	0.4%<br>0.4%	0.852	0.004
1<br>2	9997	spironolactone		1,588<br>1,556	42.7%<br>41.8%	0.453	0.017
1<br>2	298869	eplerenone		123<br>124	3.3%<br>3.3%	0.948	0.002

Laboratory

Cohort			Mean ± SD	Patients	% of Cohort	P-Value	Std diff.
1<br>2	9072	Natriuretic peptide.B prohormone N-Terminal [Mass/volume] in Serum, Plasma or Blood	9654.0 +/- 11253.6<br>8026.8 +/- 10190.0	1,089<br>1,078	29.3%<br>29.0%	<0.001	0.152
1<br>2		0 - 0 pg/mL		1,089<br>1,078	29.3%<br>29.0%	0.779	0.007
1<br>2	2003	Left Ventricular Ejection Fraction (LVEF) (%)	26.9 +/- 10.7<br>26.2 +/- 11.4	513<br>517	13.8%<br>13.9%	0.325	0.061
1<br>2		0 - 0 %		513<br>517	13.8%<br>13.9%	0.893	0.003
1<br>2	9085	Blood Pressure, Systolic	88.6 +/- 21.3<br>89.6 +/- 20.0	3,315<br>3,323	89.1%<br>89.3%	0.054	0.047
1<br>2		0 - 0 mm[Hg]		3,315<br>3,323	89.1%<br>89.3%	0.765	0.007

1<br>2	8001	Glomerular filtration rate/1.73 sq M.predicted [Volume Rate/Area] in Serum, Plasma or Blood by Creatinine-based formula (MDRD)	60.1 +/- 40.4<br>58.3 +/- 28.9	3,470<br>3,474	93.3%<br>93.4%	0.037	0.050
1<br>2		0 - 0 mL/min/{1.73_m2}		3,480<br>3,474	93.5%<br>93.4%	0.778	0.007

Table 3. Baseline characteristics of study subjects in female gender (before and after Propensity score matching). (cohort 1, purple, midodrine group; cohort 2, green: non-midodrine group)

Cohort 1 and cohort 2 patient count before and after propensity score matching

Cohort	Patient count before matching	Patient count after matching
1 - M+, femlae	2,349	2,057
2 - M-, female	3,133	2,057

Propensity score density function - Before and after matching (cohort 1 - purple, cohort 2 - green)

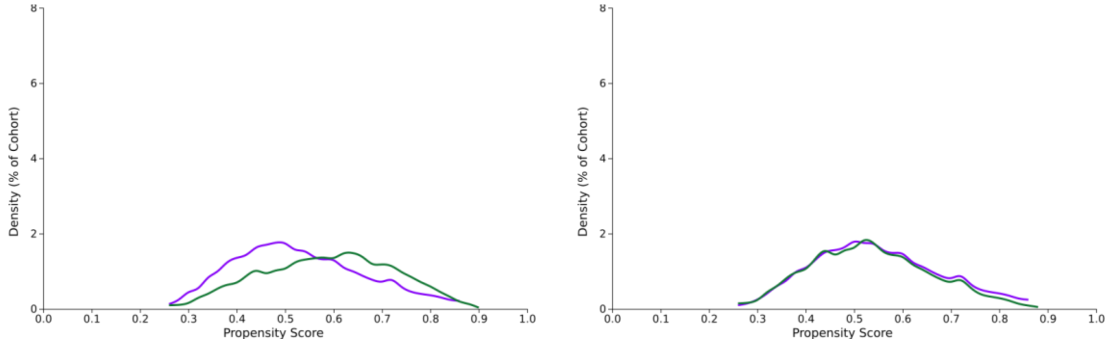

Cohort 1 (N = 2,349) and cohort 2 (N = 3,133) characteristics before propensity score matching

Demographics

Cohort			Mean ± SD	Patients	% of Cohort	P-Value	Std diff.
1	AI	Age at Index	63.9 +/- 13.8	2,349	100%	<0.001	0.223
2			60.7 +/- 14.7	3,133	100%		
1	2106-3	White		1,504	64.0%	<0.001	0.183
2				1,725	55.1%		
1	2054-5	Black or African American		554	23.6%	<0.001	0.151
2				948	30.3%		
1	M	Male		0	0%	--	--
2				0	0%		
1	2028-9	Asian		42	1.8%	0.258	0.031
2				44	1.4%		

Diagnosis

Cohort			Mean ± SD	Patients	% of Cohort	P-Value	Std diff.
1	I20-I25	Ischemic heart diseases		1,580	67.3%	0.050	0.053
2				2,028	64.7%		
1	E08-E13	Diabetes mellitus		1,099	46.8%	0.019	0.064
2				1,366	43.6%		
1	I42	Cardiomyopathy		1,126	47.9%	<0.001	0.231

2				1,861	59.4%		
1<br>2	J44	Other chronic obstructive pulmonary disease		789<br>969	33.6%<br>30.9%	0.037	0.057

Medication

Cohort			Mean ± SD	Patients	% of Cohort	P-Value	Std diff.
1<br>2	CV100	BETA BLOCKERS/RELATED		1,860<br>2,513	79.2%<br>80.2%	0.348	0.026
1<br>2	1649480	ivabradine		48<br>63	2.0%<br>2.0%	0.932	0.002
1<br>2	CV805	ANGIOTENSIN II INHIBITOR		692<br>1,078	29.5%<br>34.4%	<0.001	0.106
1<br>2	CV800	ACE INHIBITORS		704<br>1,329	30.0%<br>42.4%	<0.001	0.261
1<br>2	1545653	empagliflozin		136<br>211	5.8%<br>6.7%	0.155	0.039
1<br>2	1488564	dapagliflozin		118<br>177	5.0%<br>5.6%	0.309	0.028
1<br>2	1373458	canagliflozin		13<br>12	0.6%<br>0.4%	0.354	0.025
1<br>2	9997	spironolactone		891<br>1,514	37.9%<br>48.3%	<0.001	0.211
1<br>2	298869	eplerenone		19<br>33	0.8%<br>1.1%	0.355	0.025

Laboratory

Cohort			Mean ± SD	Patients	% of Cohort	P-Value	Std diff.
1<br>2	9072	Natriuretic peptide.B prohormone N-Terminal [Mass/volume] in Serum, Plasma or Blood	10106.3 +/- 13085.1<br>9132.8 +/- 11973.3	590<br>1,103	25.1%<br>35.2%	0.123	0.078
1<br>2		0 - 0 pg/mL		590<br>1,103	25.1%<br>35.2%	<0.001	0.221
1<br>2	2003	Left Ventricular Ejection Fraction (LVEF) (%)	29.0 +/- 11.4<br>27.6 +/- 11.9	212<br>575	9.0%<br>18.4%	0.127	0.124
1<br>2		0 - 0 %		212<br>575	9.0%<br>18.4%	<0.001	0.274

1<br>2	9085	Blood Pressure, Systolic	89.0 +/- 21.9<br>90.8 +/- 20.4	2,119<br>2,751	90.2%<br>87.8%	0.003	0.085
1<br>2		0 - 0 mm[Hg]		2,119<br>2,751	90.2%<br>87.8%	0.005	0.077
1<br>2	8001	Glomerular filtration rate/1.73 sq M.predicted [Volume Rate/Area] in Serum, Plasma or Blood by Creatinine-based formula (MDRD)	58.4 +/- 42.3<br>57.1 +/- 30.0	2,211<br>2,892	94.1%<br>92.3%	0.190	0.036
1<br>2		0 - 0 mL/min/{1.73_m2}		2,213<br>2,893	94.2%<br>92.3%	0.007	0.075

Cohort 1 (N = 2,057) and cohort 2 (N = 2,057) characteristics after propensity score matching

Demographics

Cohort			Mean ± SD	Patients	% of Cohort	P-Value	Std diff.
1<br>2	AI	Age at Index	63.1 +/- 13.9<br>63.0 +/- 13.9	2,057<br>2,057	100%<br>100%	0.830	0.007
1<br>2	2106-3	White		1,265<br>1,270	61.5%<br>61.7%	0.873	0.005
1<br>2	2054-5	Black or African American		524<br>537	25.5%<br>26.1%	0.643	0.014
1<br>2	M	Male		0<br>0	0%<br>0%	--	--
1<br>2	2028-9	Asian		34<br>31	1.7%<br>1.5%	0.708	0.012

Diagnosis

Cohort			Mean ± SD	Patients	% of Cohort	P-Value	Std diff.
1<br>2	I20-I25	Ischemic heart diseases		1,351<br>1,360	65.7%<br>66.1%	0.767	0.009
1<br>2	E08-E13	Diabetes mellitus		946<br>933	46.0%<br>45.4%	0.684	0.013
1<br>2	I42	Cardiomyopathy		1,049<br>1,040	51.0%<br>50.6%	0.779	0.009
1<br>2	J44	Other chronic obstructive pulmonary disease		672<br>678	32.7%<br>33.0%	0.842	0.006

Medication

Cohort			Mean ± SD	Patients	% of Cohort	P-Value	Std diff.
1<br>2	CV100	BETA BLOCKERS/RELATED		1,619<br>1,612	78.7%<br>78.4%	0.790	0.008
1<br>2	1649480	ivabradine		43<br>34	2.1%<br>1.7%	0.300	0.032
1<br>2	CV805	ANGIOTENSIN II INHIBITOR		634<br>601	30.8%<br>29.2%	0.262	0.035
1<br>2	CV800	ACE INHIBITORS		675<br>680	32.8%<br>33.1%	0.868	0.005
1<br>2	1545653	empagliflozin		124<br>125	6.0%<br>6.1%	0.948	0.002
1<br>2	1488564	dapagliflozin		113<br>106	5.5%<br>5.2%	0.627	0.015
1<br>2	1373458	canagliflozin		10<br>10	0.5%<br>0.5%	1	<0.001
1<br>2	9997	spironolactone		846<br>823	41.1%<br>40.0%	0.465	0.023
1<br>2	298869	eplerenone		19<br>13	0.9%<br>0.6%	0.287	0.033

Laboratory

Cohort			Mean ± SD	Patients	% of Cohort	P-Value	Std diff.
1<br>2	9072	Natriuretic peptide.B prohormone N-Terminal [Mass/volume] in Serum, Plasma or Blood	9945.4 +/- 12940.8<br>9651.9 +/- 12705.3	566<br>575	27.5%<br>28.0%	0.699	0.023
1<br>2		0 - 0 pg/mL		566<br>575	27.5%<br>28.0%	0.754	0.010
1<br>2	2003	Left Ventricular Ejection Fraction (LVEF) (%)	29.0 +/- 11.4<br>29.0 +/- 11.3	212<br>217	10.3%<br>10.5%	0.996	<0.001
1<br>2		0 - 0 %		212<br>217	10.3%<br>10.5%	0.799	0.008
1<br>2	9085	Blood Pressure, Systolic	88.9 +/- 21.7<br>91.8 +/- 20.4	1,839<br>1,853	89.4%<br>90.1%	<0.001	0.137
1		0 - 0 mm[Hg]		1,839	89.4%	0.472	0.022

2				1,853	90.1%		
1<br>2	8001	Glomerular filtration rate/1.73 sq M.predicted [Volume Rate/Area] in Serum, Plasma or Blood by Creatinine-based formula (MDRD)	58.3 +/- 41.8<br>56.0 +/- 29.4	1,922<br>1,925	93.4%<br>93.6%	0.047	0.064
1<br>2		0 - 0 mL/min/{1.73_m2}		1,924<br>1,925	93.5%<br>93.6%	0.949	0.002

Table 4. Baseline characteristics of study subjects in young age (18-<65 y/o) (before and after Propensity score matching). (cohort 1, purple, midodrine group; cohort 2, green: non-midodrine group)

Cohort 1 and cohort 2 patient count before and after propensity score matching

Cohort	Patient count before matching	Patient count after matching
1 - M+, young	2,192	2,092
2 - M-, young	3,909	2,092

Propensity score density function - Before and after matching (cohort 1 - purple, cohort 2 - green)

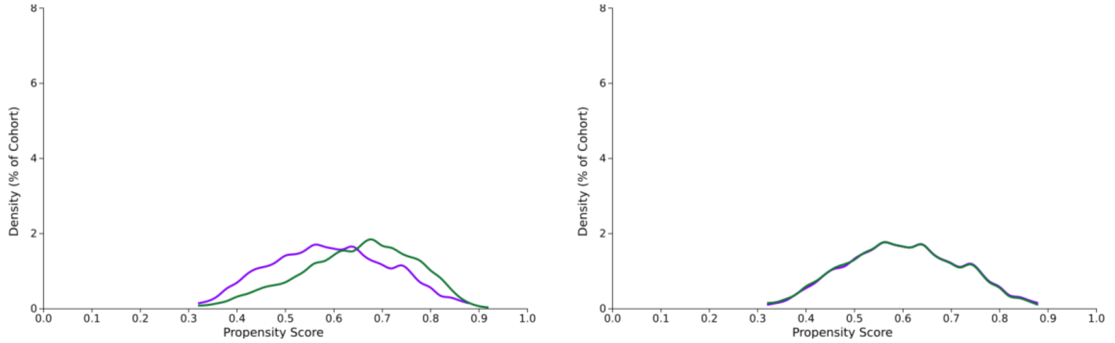

Cohort 1 (N = 2,192) and cohort 2 (N = 3,909) characteristics before propensity score matching

Demographics

Cohort			Mean ± SD	Patients	% of Cohort	P-Value	Std diff.
1	AI	Age at Index	50.6 +/- 9.8	2,192	100%	<0.001	0.204
2			48.6 +/- 10.1	3,909	100%		
1	2106-3	White		1,251	57.1%	<0.001	0.163
2				1,914	49.0%		
1	2054-5	Black or African American		573	26.1%	<0.001	0.168
2				1,321	33.8%		
1	M	Male		1,292	58.9%	<0.001	0.110
2				2,512	64.3%		
1	2028-9	Asian		51	2.3%	0.651	0.012
2				84	2.1%		

Diagnosis

Cohort			Mean ± SD	Patients	% of Cohort	P-Value	Std diff.
1	I20-I25	Ischemic heart diseases		1,397	63.7%	0.152	0.038
2				2,419	61.9%		
1	E08-E13	Diabetes mellitus		940	42.9%	0.226	0.032
2				1,614	41.3%		
1	I42	Cardiomyopathy		1,270	57.9%	<0.001	0.241

Cohort	Code	Name	Mean ± SD	Patients	% of Cohort	P-Value	Std diff.
2				2,714	69.4%		
1	J44	Other chronic obstructive pulmonary disease		546	24.9%	<0.001	0.108
2				798	20.4%		

Medication

Cohort			Mean ± SD	Patients	% of Cohort	P-Value	Std diff.
1	CV100	BETA BLOCKERS/RELATED		1,670	76.2%	<0.001	0.102
2				3,143	80.4%		
1	1649480	ivabradine		69	3.1%	0.788	0.007
2				128	3.3%		
1	CV805	ANGIOTENSIN II INHIBITOR		723	33.0%	<0.001	0.135
2				1,542	39.4%		
1	CV800	ACE INHIBITORS		737	33.6%	<0.001	0.232
2				1,755	44.9%		
1	1545653	empagliflozin		194	8.9%	0.839	0.005
2				352	9.0%		
1	1488564	dapagliflozin		163	7.4%	0.657	0.012
2				303	7.8%		
1	1373458	canagliflozin		12	0.5%	0.657	0.012
2				25	0.6%		
1	9997	spironolactone		986	45.0%	<0.001	0.194
2				2,135	54.6%		
1	298869	eplerenone		57	2.6%	0.008	0.073
2				152	3.9%		

Laboratory

Cohort			Mean ± SD	Patients	% of Cohort	P-Value	Std diff.
1	9072	Natriuretic peptide.B prohormone N-Terminal [Mass/volume] in Serum, Plasma or Blood	7997.0 +/- 9724.9	562	25.6%	<0.001	0.229
2			5969.8 +/- 7865.0	1,275	32.6%		
1		0 - 0 pg/mL		562	25.6%	<0.001	0.154
2				1,275	32.6%		
1	2003	Left Ventricular Ejection Fraction (LVEF) (%)	25.9 +/- 11.1	262	12.0%	0.064	0.134
2			24.4 +/- 11.4	744	19.0%		
1		0 - 0 %		262	12.0%	<0.001	0.197
2				744	19.0%		

1<br>2	9085	Blood Pressure, Systolic	88.0 +/- 21.3<br>88.5 +/- 20.9	1,985<br>3,341	90.6%<br>85.5%	0.392	0.024
1<br>2		0 - 0 mm[Hg]		1,985<br>3,341	90.6%<br>85.5%	<0.001	0.157
1<br>2	8001	Glomerular filtration rate/1.73 sq M.predicted [Volume Rate/Area] in Serum, Plasma or Blood by Creatinine-based formula (MDRD)	68.0 +/- 46.7<br>65.5 +/- 31.7	2,049<br>3,600	93.5%<br>92.1%	0.015	0.064
1<br>2		0 - 0 mL/min/{1.73_m2}		2,061<br>3,602	94.0%<br>92.1%	0.006	0.074

Cohort 1 (N = 2,092) and cohort 2 (N = 2,092) characteristics after propensity score matching

Demographics

Cohort			Mean ± SD	Patients	% of Cohort	P-Value	Std diff.
1<br>2	AI	Age at Index	50.4 +/- 9.9<br>50.6 +/- 9.3	2,092<br>2,092	100%<br>100%	0.501	0.021
1<br>2	2106-3	White		1,171<br>1,167	56.0%<br>55.8%	0.901	0.004
1<br>2	2054-5	Black or African American		566<br>563	27.1%<br>26.9%	0.917	0.003
1<br>2	M	Male		1,248<br>1,273	59.7%<br>60.9%	0.430	0.024
1<br>2	2028-9	Asian		50<br>47	2.4%<br>2.2%	0.758	0.010

Diagnosis

Cohort			Mean ± SD	Patients	% of Cohort	P-Value	Std diff.
1<br>2	I20-I25	Ischemic heart diseases		1,327<br>1,323	63.4%<br>63.2%	0.898	0.004
1<br>2	E08-E13	Diabetes mellitus		890<br>890	42.5%<br>42.5%	1	<0.001
1<br>2	I42	Cardiomyopathy		1,249<br>1,240	59.7%<br>59.3%	0.777	0.009
1<br>2	J44	Other chronic obstructive pulmonary disease		502<br>510	24.0%<br>24.4%	0.773	0.009

Medication

Cohort			Mean ± SD	Patients	% of Cohort	P-Value	Std diff.
1<br>2	CV100	BETA BLOCKERS/RELATED		1,602<br>1,614	76.6%<br>77.2%	0.660	0.014
1<br>2	1649480	ivabradine		67<br>62	3.2%<br>3.0%	0.655	0.014
1<br>2	CV805	ANGIOTENSIN II INHIBITOR		715<br>712	34.2%<br>34.0%	0.922	0.003
1<br>2	CV800	ACE INHIBITORS		729<br>728	34.8%<br>34.8%	0.974	0.001
1<br>2	1545653	empagliflozin		185<br>183	8.8%<br>8.7%	0.913	0.003
1<br>2	1488564	dapagliflozin		162<br>154	7.7%<br>7.4%	0.640	0.014
1<br>2	1373458	canagliflozin		12<br>10	0.6%<br>0.5%	0.669	0.013
1<br>2	9997	spironolactone		969<br>965	46.3%<br>46.1%	0.901	0.004
1<br>2	298869	eplerenone		57<br>63	2.7%<br>3.0%	0.578	0.017

Laboratory

Cohort			Mean ± SD	Patients	% of Cohort	P-Value	Std diff.
1<br>2	9072	Natriuretic peptide.B prohormone N-Terminal [Mass/volume] in Serum, Plasma or Blood	7965.7 +/- 9732.0<br>5767.8 +/- 7452.3	558<br>567	26.7%<br>27.1%	<0.001	0.254
1<br>2		0 - 0 pg/mL		558<br>567	26.7%<br>27.1%	0.754	0.010
1<br>2	2003	Left Ventricular Ejection Fraction (LVEF) (%)	25.9 +/- 11.1<br>26.4 +/- 12.4	262<br>290	12.5%<br>13.9%	0.614	0.043
1<br>2		0 - 0 %		262<br>290	12.5%<br>13.9%	0.201	0.040
1<br>2	9085	Blood Pressure, Systolic	88.0 +/- 21.4<br>89.5 +/- 21.4	1,885<br>1,903	90.1%<br>91.0%	0.040	0.067
1		0 - 0 mm[Hg]		1,885	90.1%	0.342	0.029

2				1,903	91.0%		
1<br>2	8001	Glomerular filtration rate/1.73 sq M.predicted [Volume Rate/Area] in Serum, Plasma or Blood by Creatinine-based formula (MDRD)	68.1 +/- 46.8<br>64.4 +/- 32.5	1,949<br>1,965	93.2%<br>93.9%	0.004	0.091
1<br>2		0 - 0 mL/min/{1.73_m2}		1,961<br>1,965	93.7%<br>93.9%	0.797	0.008

Table 5. Baseline characteristics of study subjects in old age (≥65 y/o) (before and after Propensity score matching). (cohort 1, purple, midodrine group; cohort 2, green: non-midodrine group)

Cohort 1 and cohort 2 patient count before and after propensity score matching

Cohort	Patient count before matching	Patient count after matching
1 - M+ ,old	4,243	3,707
2 - M-, old	5,265	3,707

Propensity score density function - Before and after matching (cohort 1 - purple, cohort 2 - green)

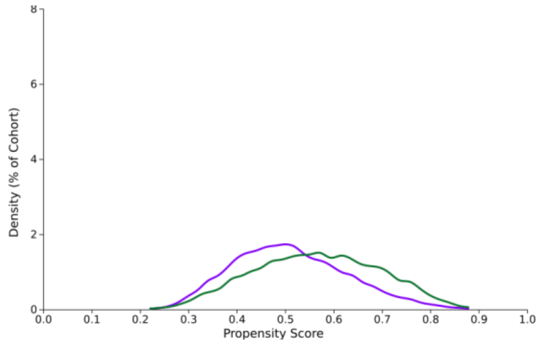

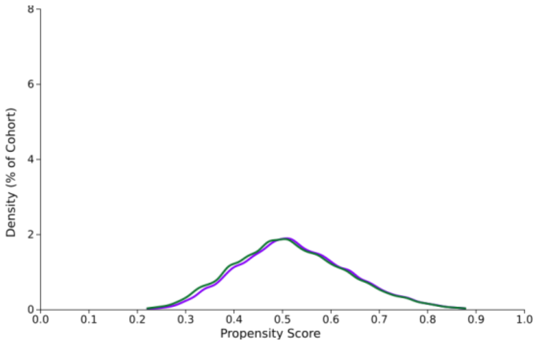

Cohort 1 (N = 4,243) and cohort 2 (N = 5,265) characteristics before propensity score matching

Demographics

Cohort			Mean ± SD	Patients	% of Cohort	P-Value	Std diff.
1	AI	Age at Index	72.3 +/- 7.1	4,243	100%	<0.001	0.222
2			70.7 +/- 7.2	5,265	100%		
1	2106-3	White		3,043	71.7%	<0.001	0.143
2				3,428	65.1%		
1	2054-5	Black or African American		637	15.0%	<0.001	0.119
2				1,027	19.5%		
1	M	Male		2,793	65.8%	0.694	0.008
2				3,486	66.2%		
1	2028-9	Asian		96	2.3%	0.008	0.055
2				80	1.5%		

Diagnosis

Cohort			Mean ± SD	Patients	% of Cohort	P-Value	Std diff.
1	I20-I25	Ischemic heart diseases		3,218	75.8%	0.050	0.040
2				4,083	77.5%		
1	E08-E13	Diabetes mellitus		2,044	48.2%	0.048	0.041
2				2,429	46.1%		
1	I42	Cardiomyopathy		1,923	45.3%	<0.001	0.224

2				2,971	56.4%		
1<br>2	J44	Other chronic obstructive pulmonary disease		1,444<br>1,760	34.0%<br>33.4%	0.536	0.013
**Medication**							
Cohort			Mean ± SD	Patients	% of Cohort	P-Value	Std diff.
1<br>2	CV100	BETA BLOCKERS/RELATED		3,354<br>4,240	79.0%<br>80.5%	0.073	0.037
1<br>2	1649480	ivabradine		53<br>72	1.2%<br>1.4%	0.614	0.010
1<br>2	CV805	ANGIOTENSIN II INHIBITOR		1,312<br>1,755	30.9%<br>33.3%	0.012	0.052
1<br>2	CV800	ACE INHIBITORS		1,310<br>2,189	30.9%<br>41.6%	<0.001	0.224
1<br>2	1545653	empagliflozin		252<br>343	5.9%<br>6.5%	0.249	0.024
1<br>2	1488564	dapagliflozin		162<br>235	3.8%<br>4.5%	0.118	0.032
1<br>2	1373458	canagliflozin		12<br>20	0.3%<br>0.4%	0.417	0.017
1<br>2	9997	spironolactone		1,427<br>2,200	33.6%<br>41.8%	<0.001	0.169
1<br>2	298869	eplerenone		79<br>172	1.9%<br>3.3%	<0.001	0.089
**Laboratory**							
Cohort			Mean ± SD	Patients	% of Cohort	P-Value	Std diff.
1<br>2	9072	Natriuretic peptide.B prohormone N-Terminal [Mass/volume] in Serum, Plasma or Blood	10118.1 +/- 11928.3<br>9865.9 +/- 11979.0	1,084<br>1,914	25.5%<br>36.4%	0.579	0.021
1<br>2		0 - 0 pg/mL		1,084<br>1,914	25.5%<br>36.4%	<0.001	0.235
1<br>2	2003	Left Ventricular Ejection Fraction (LVEF) (%)	28.5 +/- 10.4<br>26.7 +/- 11.3	434<br>1,011	10.2%<br>19.2%	0.004	0.167
1<br>2		0 - 0 %		434<br>1,011	10.2%<br>19.2%	<0.001	0.255

Cohort	Code	Characteristic	Mean ± SD	Patients	% of Cohort	P-Value	Std diff.
1<br>2	9085	Blood Pressure, Systolic	89.4 +/- 23.0<br>90.8 +/- 19.6	3,684<br>4,579	86.8%<br>87.0%	0.003	0.064
1<br>2		0 - 0 mm[Hg]		3,684<br>4,579	86.8%<br>87.0%	0.835	0.004
1<br>2	8001	Glomerular filtration rate/1.73 sq M.predicted [Volume Rate/Area] in Serum, Plasma or Blood by Creatinine-based formula (MDRD)	53.8 +/- 35.5<br>52.9 +/- 26.0	3,876<br>4,775	91.4%<br>90.7%	0.194	0.028
1<br>2		0 - 0 mL/min/{1.73_m2}		3,876<br>4,776	91.4%<br>90.7%	0.280	0.022

Cohort 1 (N = 3,707) and cohort 2 (N = 3,707) characteristics after propensity score matching

Demographics

Cohort			Mean ± SD	Patients	% of Cohort	P-Value	Std diff.
1<br>2	AI	Age at Index	71.8 +/- 7.0<br>71.7 +/- 7.2	3,707<br>3,707	100%<br>100%	0.414	0.019
1<br>2	2106-3	White		2,577<br>2,599	69.5%<br>70.1%	0.578	0.013
1<br>2	2054-5	Black or African American		610<br>572	16.5%<br>15.4%	0.228	0.028
1<br>2	M	Male		2,445<br>2,467	66.0%<br>66.5%	0.589	0.013
1<br>2	2028-9	Asian		74<br>69	2.0%<br>1.9%	0.673	0.010

Diagnosis

Cohort			Mean ± SD	Patients	% of Cohort	P-Value	Std diff.
1<br>2	I20-I25	Ischemic heart diseases		2,811<br>2,807	75.8%<br>75.7%	0.914	0.003
1<br>2	E08-E13	Diabetes mellitus		1,749<br>1,758	47.2%<br>47.4%	0.834	0.005
1<br>2	I42	Cardiomyopathy		1,802<br>1,802	48.6%<br>48.6%	1	<0.001
1<br>2	J44	Other chronic obstructive pulmonary disease		1,245<br>1,259	33.6%<br>34.0%	0.731	0.008

**Medication**								
Cohort			Mean ± SD	Patients	% of Cohort	P-Value	Std diff.
1<br>2	CV100	BETA BLOCKERS/RELATED		2,914<br>2,905	78.6%<br>78.4%	0.799	0.006
1<br>2	1649480	ivabradine		47<br>46	1.3%<br>1.2%	0.917	0.002
1<br>2	CV805	ANGIOTENSIN II INHIBITOR		1,167<br>1,157	31.5%<br>31.2%	0.802	0.006
1<br>2	CV800	ACE INHIBITORS		1,251<br>1,244	33.7%<br>33.6%	0.863	0.004
1<br>2	1545653	empagliflozin		231<br>234	6.2%<br>6.3%	0.886	0.003
1<br>2	1488564	dapagliflozin		150<br>142	4.0%<br>3.8%	0.633	0.011
1<br>2	1373458	canagliflozin		11<br>11	0.3%<br>0.3%	1	<0.001
1<br>2	9997	spironolactone		1,319<br>1,332	35.6%<br>35.9%	0.753	0.007
1<br>2	298869	eplerenone		79<br>77	2.1%<br>2.1%	0.871	0.004

**Laboratory**							
Cohort			Mean ± SD	Patients	% of Cohort	P-Value	Std diff.
1<br>2	9072	Natriuretic peptide.B prohormone N-Terminal [Mass/volume] in Serum, Plasma or Blood	10112.2 +/- 11885.8<br>9931.9 +/- 12348.2	1,048<br>1,046	28.3%<br>28.2%	0.734	0.015
1<br>2		0 - 0 pg/mL		1,048<br>1,046	28.3%<br>28.2%	0.959	0.001
1<br>2	2003	Left Ventricular Ejection Fraction (LVEF) (%)	28.6 +/- 10.4<br>27.6 +/- 11.4	429<br>426	11.6%<br>11.5%	0.180	0.092
1<br>2		0 - 0 %		429<br>426	11.6%<br>11.5%	0.913	0.003
1<br>2	9085	Blood Pressure, Systolic	89.3 +/- 23.0<br>91.1 +/- 20.0	3,197<br>3,201	86.2%<br>86.4%	0.001	0.083
1		0 - 0 mm[Hg]		3,197	86.2%	0.893	0.003

2				3,201	86.4%		
1<br>2	8001	Glomerular filtration rate/1.73 sq M.predicted [Volume Rate/Area] in Serum, Plasma or Blood by Creatinine-based formula (MDRD)	54.0 +/- 35.4<br>53.0 +/- 26.0	3,355<br>3,359	90.5%<br>90.6%	0.168	0.034
1<br>2		0 - 0 mL/min/{1.73_m2}		3,355<br>3,360	90.5%<br>90.6%	0.842	0.005

Table 6. Baseline characteristics of study subjects in frequency of medication (at least 3 times of medication refills) (before and after Propensity score matching). (cohort 1, purple, midodrine group; cohort 2, green: non-midodrine group)

Cohort 1 and cohort 2 patient count before and after propensity score matching

Cohort	Patient count before matching	Patient count after matching
1 - midodrine>=3	4,095	4,063
2 - midodrine- 10yrs;Hypotension>15	10,632	4,063

Propensity score density function - Before and after matching (cohort 1 - purple, cohort 2 - green)

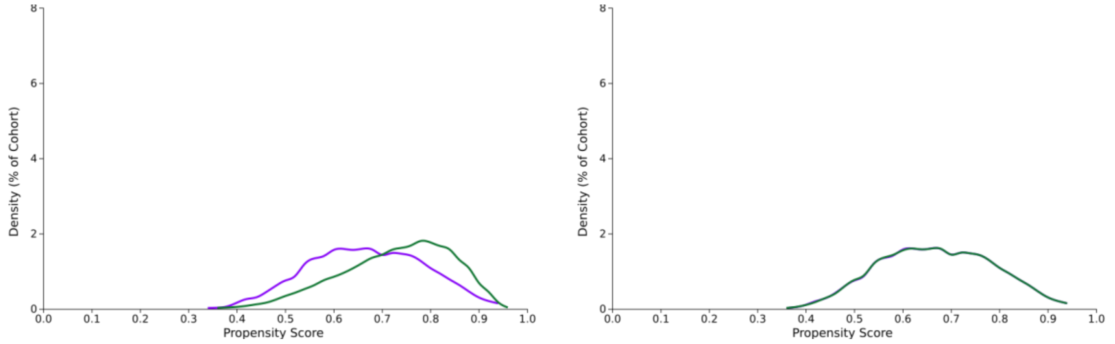

Cohort 1 (N = 4,095) and cohort 2 (N = 10,632) characteristics before propensity score matching

Demographics

Cohort			Mean ± SD	Patients	% of Cohort	P-Value	Std diff.
1	AI	Age at Index	65.1 +/- 13.1	4,095	100%	<0.001	0.342
2			60.4 +/- 14.4	10,632	100%		
1	2106-3	White		2,761	67.4%	<0.001	0.217
2				6,056	57.0%		
1	2054-5	Black or African American		758	18.5%	<0.001	0.175
2				2,737	25.7%		
1	M	Male		2,572	62.8%	0.001	0.060
2				6,983	65.7%		
1	2028-9	Asian		100	2.4%	0.034	0.038
2				201	1.9%		

Diagnosis

Cohort			Mean ± SD	Patients	% of Cohort	P-Value	Std diff.
1	I20-I25	Ischemic heart diseases		3,018	73.7%	<0.001	0.087
2				7,422	69.8%		
1	E08-E13	Diabetes mellitus		1,945	47.5%	<0.001	0.093
2				4,558	42.9%		
1	I42	Cardiomyopathy		2,074	50.6%	<0.001	0.200

2				6,436	60.5%		
1<br>2	J44	Other chronic obstructive pulmonary disease		1,347<br>2,962	32.9%<br>27.9%	<0.001	0.110

Medication

Cohort			Mean ± SD	Patients	% of Cohort	P-Value	Std diff.
1<br>2	CV100	BETA BLOCKERS/RELATED		3,274<br>8,515	80.0%<br>80.1%	0.852	0.003
1<br>2	1649480	ivabradine		83<br>243	2.0%<br>2.3%	0.339	0.018
1<br>2	CV805	ANGIOTENSIN II INHIBITOR		1,258<br>3,741	30.7%<br>35.2%	<0.001	0.095
1<br>2	CV800	ACE INHIBITORS		1,309<br>4,558	32.0%<br>42.9%	<0.001	0.227
1<br>2	1545653	empagliflozin		277<br>789	6.8%<br>7.4%	0.168	0.026
1<br>2	1488564	dapagliflozin		220<br>616	5.4%<br>5.8%	0.322	0.018
1<br>2	1373458	canagliflozin		21<br>49	0.5%<br>0.5%	0.681	0.007
1<br>2	9997	spironolactone		1,623<br>4,863	39.6%<br>45.7%	<0.001	0.124
1<br>2	298869	eplerenone		92<br>375	2.2%<br>3.5%	<0.001	0.077

Laboratory

Cohort			Mean ± SD	Patients	% of Cohort	P-Value	Std diff.
1<br>2	9072	Natriuretic peptide.B prohormone N-Terminal [Mass/volume] in Serum, Plasma or Blood	10085.6 +/- 12296.2<br>8047.8 +/- 10483.5	1,033<br>3,681	25.2%<br>34.6%	<0.001	0.178
1<br>2		0 - 0 pg/mL		1,033<br>3,681	25.2%<br>34.6%	<0.001	0.206
1<br>2	2003	Left Ventricular Ejection Fraction (LVEF) (%)	27.7 +/- 10.6<br>25.9 +/- 11.6	436<br>2,011	10.6%<br>18.9%	0.003	0.164
1<br>2		0 - 0 %		436<br>2,011	10.6%<br>18.9%	<0.001	0.235

1<br>2	9085	Blood Pressure, Systolic	89.5 +/- 20.8<br>89.9 +/- 20.2	3,736<br>9,159	91.2%<br>86.1%	0.380	0.017
1<br>2		0 - 0 mm[Hg]		3,736<br>9,159	91.2%<br>86.1%	<0.001	0.161
1<br>2	8001	Glomerular filtration rate/1.73 sq M.predicted [Volume Rate/Area] in Serum, Plasma or Blood by Creatinine-based formula (MDRD)	59.9 +/- 41.5<br>64.9 +/- 36.2	3,836<br>9,753	93.7%<br>91.7%	<0.001	0.127
1<br>2		0 - 0 mL/min/{1.73_m2}		3,844<br>9,770	93.9%<br>91.9%	<0.001	0.077

Cohort 1 (N = 4,063) and cohort 2 (N = 4,063) characteristics after propensity score matching

Demographics

Cohort			Mean ± SD	Patients	% of Cohort	P-Value	Std diff.
1<br>2	AI	Age at Index	65.0 +/- 13.1<br>64.7 +/- 13.1	4,063<br>4,063	100%<br>100%	0.269	0.025
1<br>2	2106-3	White		2,732<br>2,744	67.2%<br>67.5%	0.776	0.006
1<br>2	2054-5	Black or African American		758<br>740	18.7%<br>18.2%	0.607	0.011
1<br>2	M	Male		2,561<br>2,549	63.0%<br>62.7%	0.783	0.006
1<br>2	2028-9	Asian		97<br>94	2.4%<br>2.3%	0.826	0.005

Diagnosis

Cohort			Mean ± SD	Patients	% of Cohort	P-Value	Std diff.
1<br>2	I20-I25	Ischemic heart diseases		2,988<br>2,953	73.5%<br>72.7%	0.381	0.019
1<br>2	E08-E13	Diabetes mellitus		1,918<br>1,902	47.2%<br>46.8%	0.722	0.008
1<br>2	I42	Cardiomyopathy		2,070<br>2,035	50.9%<br>50.1%	0.437	0.017
1<br>2	J44	Other chronic obstructive pulmonary disease		1,328<br>1,318	32.7%<br>32.4%	0.813	0.005

Medication

Cohort			Mean ± SD	Patients	% of Cohort	P-Value	Std diff.
1<br>2	CV100	BETA BLOCKERS/RELATED		3,248<br>3,215	79.9%<br>79.1%	0.364	0.020
1<br>2	1649480	ivabradine		83<br>87	2.0%<br>2.1%	0.757	0.007
1<br>2	CV805	ANGIOTENSIN II INHIBITOR		1,257<br>1,278	30.9%<br>31.5%	0.615	0.011
1<br>2	CV800	ACE INHIBITORS		1,309<br>1,271	32.2%<br>31.3%	0.365	0.020
1<br>2	1545653	empagliflozin		276<br>268	6.8%<br>6.6%	0.723	0.008
1<br>2	1488564	dapagliflozin		219<br>219	5.4%<br>5.4%	1	<0.001
1<br>2	1373458	canagliflozin		21<br>16	0.5%<br>0.4%	0.410	0.018
1<br>2	9997	spironolactone		1,616<br>1,609	39.8%<br>39.6%	0.874	0.004
1<br>2	298869	eplerenone		92<br>99	2.3%<br>2.4%	0.608	0.011

Laboratory

Cohort			Mean ± SD	Patients	% of Cohort	P-Value	Std diff.
1<br>2	9072	Natriuretic peptide.B prohormone N-Terminal [Mass/volume] in Serum, Plasma or Blood	10085.6 +/- 12296.2<br>8388.9 +/- 11367.5	1,033<br>984	25.4%<br>24.2%	0.001	0.143
1<br>2		0 - 0 pg/mL		1,033<br>984	25.4%<br>24.2%	0.208	0.028
1<br>2	2003	Left Ventricular Ejection Fraction (LVEF) (%)	27.7 +/- 10.6<br>27.6 +/- 12.2	436<br>436	10.7%<br>10.7%	0.951	0.004
1<br>2		0 - 0 %		436<br>436	10.7%<br>10.7%	1	<0.001
1<br>2	9085	Blood Pressure, Systolic	89.6 +/- 20.7<br>90.6 +/- 20.1	3,704<br>3,706	91.2%<br>91.2%	0.023	0.053
1		0 - 0 mm[Hg]		3,704	91.2%	0.938	0.002

2				3,706	91.2%		
1<br>2	8001	Glomerular filtration rate/1.73 sq M.predicted [Volume Rate/Area] in Serum, Plasma or Blood by Creatinine-based formula (MDRD)	60.0 +/- 41.6<br>63.3 +/- 36.1	3,804<br>3,796	93.6%<br>93.4%	<0.001	0.086
1<br>2		0 - 0 mL/min/{1.73_m2}		3,812<br>3,800	93.8%<br>93.5%	0.584	0.012

Table 7. Baseline characteristics of study subjects in frequency of medication (at least 6 times of medication refills) (before and after Propensity score matching). (cohort 1, purple, midodrine group; cohort 2, green: non-midodrine group)

Cohort 1 and cohort 2 patient count before and after propensity score matching

Cohort	Patient count before matching	Patient count after matching
1 - modidrine>=6	2,282	2,280
2 - midodrine- 10yrs;Hypotension>15	10,632	2,280

Propensity score density function - Before and after matching (cohort 1 - purple, cohort 2 - green)

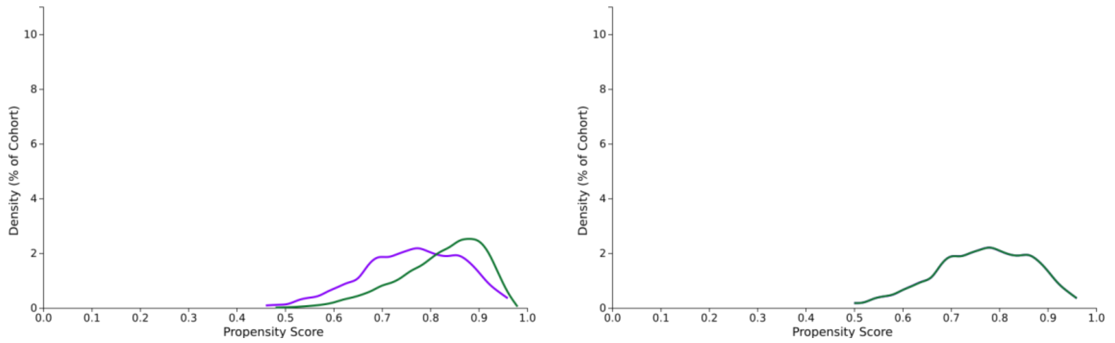

Cohort 1 (N = 2,282) and cohort 2 (N = 10,632) characteristics before propensity score matching

Demographics

Cohort			Mean ± SD	Patients	% of Cohort	P-Value	Std diff.
1	AI	Age at Index	65.1 +/- 13.1	2,282	100%	<0.001	0.345
2			60.4 +/- 14.4	10,632	100%		
1	2106-3	White		1,554	68.1%	<0.001	0.232
2				6,056	57.0%		
1	2054-5	Black or African American		426	18.7%	<0.001	0.171
2				2,737	25.7%		
1	M	Male		1,440	63.1%	0.019	0.054
2				6,983	65.7%		
1	2028-9	Asian		55	2.4%	0.106	0.036
2				201	1.9%		

Diagnosis

Cohort			Mean ± SD	Patients	% of Cohort	P-Value	Std diff.
1	I20-I25	Ischemic heart diseases		1,673	73.3%	0.001	0.078
2				7,422	69.8%		
1	E08-E13	Diabetes mellitus		1,077	47.2%	<0.001	0.087
2				4,558	42.9%		
1	I42	Cardiomyopathy		1,148	50.3%	<0.001	0.207

Cohort			Mean ± SD	Patients	% of Cohort	P-Value	Std diff.
2				6,436	60.5%		
1<br>2	J44	Other chronic obstructive pulmonary disease		769<br>2,962	33.7%<br>27.9%	<0.001	0.127
**Medication**							
Cohort			Mean ± SD	Patients	% of Cohort	P-Value	Std diff.
1<br>2	CV100	BETA BLOCKERS/RELATED		1,807<br>8,515	79.2%<br>80.1%	0.328	0.022
1<br>2	1649480	ivabradine		42<br>243	1.8%<br>2.3%	0.189	0.031
1<br>2	CV805	ANGIOTENSIN II INHIBITOR		664<br>3,741	29.1%<br>35.2%	<0.001	0.131
1<br>2	CV800	ACE INHIBITORS		705<br>4,558	30.9%<br>42.9%	<0.001	0.250
1<br>2	1545653	empagliflozin		139<br>789	6.1%<br>7.4%	0.026	0.053
1<br>2	1488564	dapagliflozin		131<br>616	5.7%<br>5.8%	0.921	0.002
1<br>2	1373458	canagliflozin		10<br>49	0.4%<br>0.5%	0.884	0.003
1<br>2	9997	spironolactone		930<br>4,863	40.8%<br>45.7%	<0.001	0.101
1<br>2	298869	eplerenone		50<br>375	2.2%<br>3.5%	0.001	0.080
**Laboratory**							
Cohort			Mean ± SD	Patients	% of Cohort	P-Value	Std diff.
1<br>2	9072	Natriuretic peptide.B prohormone N-Terminal [Mass/volume] in Serum, Plasma or Blood	9458.3 +/- 12331.3<br>8047.8 +/- 10483.5	548<br>3,681	24.0%<br>34.6%	0.004	0.123
1<br>2		0 - 0 pg/mL		548<br>3,681	24.0%<br>34.6%	<0.001	0.235
1<br>2	2003	Left Ventricular Ejection Fraction (LVEF) (%)	26.6 +/- 10.4<br>25.9 +/- 11.6	230<br>2,011	10.1%<br>18.9%	0.346	0.068
1<br>2		0 - 0 %		230<br>2,011	10.1%<br>18.9%	<0.001	0.253

1<br>2	9085	Blood Pressure, Systolic	90.8 +/- 20.3<br>89.9 +/- 20.2	2,093<br>9,159	91.7%<br>86.1%	0.053	0.047
1<br>2		0 - 0 mm[Hg]		2,093<br>9,159	91.7%<br>86.1%	<0.001	0.178
1<br>2	8001	Glomerular filtration rate/1.73 sq M.predicted [Volume Rate/Area] in Serum, Plasma or Blood by Creatinine-based formula (MDRD)	60.5 +/- 41.9<br>64.9 +/- 36.2	2,115<br>9,753	92.7%<br>91.7%	<0.001	0.113
1<br>2		0 - 0 mL/min/{1.73_m2}		2,122<br>9,770	93.0%<br>91.9%	0.078	0.041

Cohort 1 (N = 2,280) and cohort 2 (N = 2,280) characteristics after propensity score matching

Demographics

Cohort			Mean ± SD	Patients	% of Cohort	P-Value	Std diff.
1<br>2	AI	Age at Index	65.1 +/- 13.1<br>65.1 +/- 12.7	2,280<br>2,280	100%<br>100%	0.886	0.004
1<br>2	2106-3	White		1,553<br>1,564	68.1%<br>68.6%	0.726	0.010
1<br>2	2054-5	Black or African American		426<br>411	18.7%<br>18.0%	0.566	0.017
1<br>2	M	Male		1,440<br>1,456	63.2%<br>63.9%	0.623	0.015
1<br>2	2028-9	Asian		54<br>50	2.4%<br>2.2%	0.692	0.012

Diagnosis

Cohort			Mean ± SD	Patients	% of Cohort	P-Value	Std diff.
1<br>2	I20-I25	Ischemic heart diseases		1,671<br>1,699	73.3%<br>74.5%	0.345	0.028
1<br>2	E08-E13	Diabetes mellitus		1,075<br>1,094	47.1%<br>48.0%	0.573	0.017
1<br>2	I42	Cardiomyopathy		1,148<br>1,154	50.4%<br>50.6%	0.859	0.005
1<br>2	J44	Other chronic obstructive pulmonary disease		767<br>752	33.6%<br>33.0%	0.637	0.014

Medication								
Cohort			Mean ± SD	Patients	% of Cohort	P-Value	Std diff.
1<br>2	CV100	BETA BLOCKERS/RELATED		1,805<br>1,801	79.2%<br>79.0%	0.884	0.004
1<br>2	1649480	ivabradine		42<br>38	1.8%<br>1.7%	0.652	0.013
1<br>2	CV805	ANGIOTENSIN II INHIBITOR		664<br>670	29.1%<br>29.4%	0.845	0.006
1<br>2	CV800	ACE INHIBITORS		705<br>716	30.9%<br>31.4%	0.725	0.010
1<br>2	1545653	empagliflozin		139<br>141	6.1%<br>6.2%	0.902	0.004
1<br>2	1488564	dapagliflozin		130<br>141	5.7%<br>6.2%	0.491	0.020
1<br>2	1373458	canagliflozin		10<br>10	0.4%<br>0.4%	1	<0.001
1<br>2	9997	spironolactone		929<br>912	40.7%<br>40%	0.608	0.015
1<br>2	298869	eplerenone		50<br>39	2.2%<br>1.7%	0.239	0.035

Laboratory							
Cohort			Mean ± SD	Patients	% of Cohort	P-Value	Std diff.
1<br>2	9072	Natriuretic peptide.B prohormone N-Terminal [Mass/volume] in Serum, Plasma or Blood	9458.3 +/- 12331.3<br>9864.3 +/- 12670.1	548<br>526	24.0%<br>23.1%	0.595	0.032
1<br>2		0 - 0 pg/mL		548<br>526	24.0%<br>23.1%	0.443	0.023
1<br>2	2003	Left Ventricular Ejection Fraction (LVEF) (%)	26.6 +/- 10.4<br>27.2 +/- 12.0	230<br>233	10.1%<br>10.2%	0.593	0.050
1<br>2		0 - 0 %		230<br>233	10.1%<br>10.2%	0.883	0.004
1<br>2	9085	Blood Pressure, Systolic	90.8 +/- 20.3<br>90.7 +/- 20.6	2,091<br>2,092	91.7%<br>91.8%	0.873	0.005
1		0 - 0 mm[Hg]		2,091	91.7%	0.957	0.002

2				2,092	91.8%		
1<br>2	8001	Glomerular filtration rate/1.73 sq M.predicted [Volume Rate/Area] in Serum, Plasma or Blood by Creatinine-based formula (MDRD)	60.4 +/- 41.9<br>61.7 +/- 34.5	2,113<br>2,113	92.7%<br>92.7%	0.261	0.035
1<br>2		0 - 0 mL/min/{1.73_m2}		2,120<br>2,113	93.0%<br>92.7%	0.688	0.012

Table 8. Baseline characteristics of study subjects in frequency of medication (at least 9 times of medication refills) (before and after Propensity score matching). (cohort 1, purple, midodrine group; cohort 2, green: non-midodrine group)

Cohort 1 and cohort 2 patient count before and after propensity score matching

Cohort	Patient count before matching	Patient count after matching
1 - midodrine>=9	1,490	1,488
2 - midodrine- 10yrs;Hypotension>15	10,632	1,488

Propensity score density function - Before and after matching (cohort 1 - purple, cohort 2 - green)

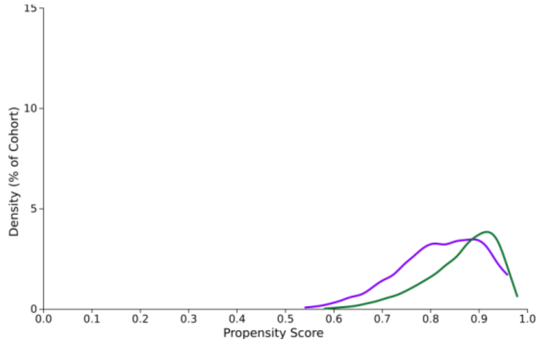

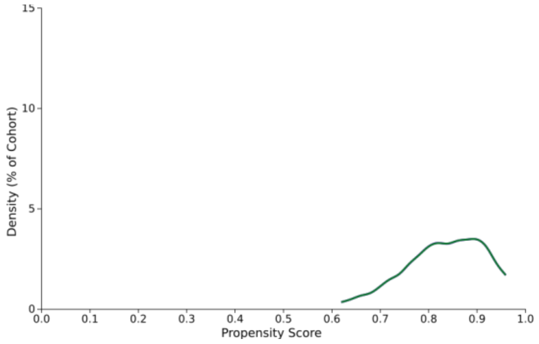

Cohort 1 (N = 1,490) and cohort 2 (N = 10,632) characteristics before propensity score matching

Demographics

Cohort			Mean ± SD	Patients	% of Cohort	P-Value	Std diff.
1	AI	Age at Index	64.6 +/- 13.1	1,490	100%	<0.001	0.304
2			60.4 +/- 14.4	10,632	100%		
1	2106-3	White		1,030	69.1%	<0.001	0.254
2				6,056	57.0%		
1	2054-5	Black or African American		266	17.9%	<0.001	0.192
2				2,737	25.7%		
1	M	Male		939	63.0%	0.043	0.056
2				6,983	65.7%		
1	2028-9	Asian		39	2.6%	0.059	0.049
2				201	1.9%		

Diagnosis

Cohort			Mean ± SD	Patients	% of Cohort	P-Value	Std diff.
1	I20-I25	Ischemic heart diseases		1,084	72.8%	0.020	0.065
2				7,422	69.8%		
1	E08-E13	Diabetes mellitus		702	47.1%	0.002	0.085
2				4,558	42.9%		
1	I42	Cardiomyopathy		754	50.6%	<0.001	0.201

2				6,436	60.5%		
1<br>2	J44	Other chronic obstructive pulmonary disease		499<br>2,962	33.5%<br>27.9%	<0.001	0.122

Medication

Cohort			Mean ± SD	Patients	% of Cohort	P-Value	Std diff.
1<br>2	CV100	BETA BLOCKERS/RELATED		1,155<br>8,515	77.5%<br>80.1%	0.021	0.063
1<br>2	1649480	ivabradine		23<br>243	1.5%<br>2.3%	0.067	0.054
1<br>2	CV805	ANGIOTENSIN II INHIBITOR		400<br>3,741	26.8%<br>35.2%	<0.001	0.181
1<br>2	CV800	ACE INHIBITORS		455<br>4,558	30.5%<br>42.9%	<0.001	0.258
1<br>2	1545653	empagliflozin		82<br>789	5.5%<br>7.4%	0.007	0.078
1<br>2	1488564	dapagliflozin		65<br>616	4.4%<br>5.8%	0.025	0.065
1<br>2	1373458	canagliflozin		10<br>49	0.7%<br>0.5%	0.275	0.028
1<br>2	9997	spironolactone		603<br>4,863	40.5%<br>45.7%	<0.001	0.107
1<br>2	298869	eplerenone		31<br>375	2.1%<br>3.5%	0.004	0.088

Laboratory

Cohort			Mean ± SD	Patients	% of Cohort	P-Value	Std diff.
1<br>2	9072	Natriuretic peptide.B prohormone N-Terminal [Mass/volume] in Serum, Plasma or Blood	9035.1 +/- 12770.9<br>8047.8 +/- 10483.5	348<br>3,681	23.4%<br>34.6%	0.100	0.084
1<br>2		0 - 0 pg/mL		348<br>3,681	23.4%<br>34.6%	<0.001	0.250
1<br>2	2003	Left Ventricular Ejection Fraction (LVEF) (%)	26.8 +/- 10.5<br>25.9 +/- 11.6	155<br>2,011	10.4%<br>18.9%	0.342	0.082
1<br>2		0 - 0 %		155<br>2,011	10.4%<br>18.9%	<0.001	0.242

1<br>2	9085	Blood Pressure, Systolic	91.2 +/- 20.2<br>89.9 +/- 20.2	1,373<br>9,159	92.1%<br>86.1%	0.026	0.064
1<br>2		0 - 0 mm[Hg]		1,373<br>9,159	92.1%<br>86.1%	<0.001	0.194
1<br>2	8001	Glomerular filtration rate/1.73 sq M.predicted [Volume Rate/Area] in Serum, Plasma or Blood by Creatinine-based formula (MDRD)	61.9 +/- 43.6<br>64.9 +/- 36.2	1,365<br>9,753	91.6%<br>91.7%	0.005	0.075
1<br>2		0 - 0 mL/min/{1.73_m2}		1,371<br>9,770	92.0%<br>91.9%	0.873	0.004

Cohort 1 (N = 1,488) and cohort 2 (N = 1,488) characteristics after propensity score matching

Demographics

Cohort			Mean ± SD	Patients	% of Cohort	P-Value	Std diff.
1<br>2	AI	Age at Index	64.6 +/- 13.1<br>64.2 +/- 12.8	1,488<br>1,488	100%<br>100%	0.480	0.026
1<br>2	2106-3	White		1,029<br>1,055	69.2%<br>70.9%	0.298	0.038
1<br>2	2054-5	Black or African American		266<br>247	17.9%<br>16.6%	0.356	0.034
1<br>2	M	Male		939<br>960	63.1%<br>64.5%	0.423	0.029
1<br>2	2028-9	Asian		38<br>33	2.6%<br>2.2%	0.548	0.022

Diagnosis

Cohort			Mean ± SD	Patients	% of Cohort	P-Value	Std diff.
1<br>2	I20-I25	Ischemic heart diseases		1,082<br>1,091	72.7%<br>73.3%	0.710	0.014
1<br>2	E08-E13	Diabetes mellitus		700<br>680	47.0%<br>45.7%	0.462	0.027
1<br>2	I42	Cardiomyopathy		754<br>742	50.7%<br>49.9%	0.660	0.016
1<br>2	J44	Other chronic obstructive pulmonary disease		497<br>490	33.4%<br>32.9%	0.785	0.010

Medication

Cohort			Mean ± SD	Patients	% of Cohort	P-Value	Std diff.
1<br>2	CV100	BETA BLOCKERS/RELATED		1,153<br>1,134	77.5%<br>76.2%	0.409	0.030
1<br>2	1649480	ivabradine		23<br>27	1.5%<br>1.8%	0.568	0.021
1<br>2	CV805	ANGIOTENSIN II INHIBITOR		400<br>411	26.9%<br>27.6%	0.651	0.017
1<br>2	CV800	ACE INHIBITORS		455<br>445	30.6%<br>29.9%	0.690	0.015
1<br>2	1545653	empagliflozin		82<br>76	5.5%<br>5.1%	0.624	0.018
1<br>2	1488564	dapagliflozin		65<br>63	4.4%<br>4.2%	0.857	0.007
1<br>2	1373458	canagliflozin		10<br>10	0.7%<br>0.7%	1	<0.001
1<br>2	9997	spironolactone		601<br>562	40.4%<br>37.8%	0.143	0.054
1<br>2	298869	eplerenone		31<br>26	2.1%<br>1.7%	0.504	0.025

Laboratory

Cohort			Mean ± SD	Patients	% of Cohort	P-Value	Std diff.
1<br>2	9072	Natriuretic peptide.B prohormone N-Terminal [Mass/volume] in Serum, Plasma or Blood	9035.1 +/- 12770.9<br>8013.3 +/- 10569.8	348<br>344	23.4%<br>23.1%	0.252	0.087
1<br>2		0 - 0 pg/mL		348<br>344	23.4%<br>23.1%	0.862	0.006
1<br>2	2003	Left Ventricular Ejection Fraction (LVEF) (%)	26.8 +/- 10.5<br>28.3 +/- 12.4	155<br>142	10.4%<br>9.5%	0.249	0.134
1<br>2		0 - 0 %		155<br>142	10.4%<br>9.5%	0.427	0.029
1<br>2	9085	Blood Pressure, Systolic	91.2 +/- 20.2<br>90.5 +/- 18.9	1,371<br>1,371	92.1%<br>92.1%	0.333	0.037
1		0 - 0 mm[Hg]		1,371	92.1%	1	<0.001

2				1,371	92.1%		
1<br>2	8001	Glomerular filtration rate/1.73 sq M.predicted [Volume Rate/Area] in Serum, Plasma or Blood by Creatinine-based formula (MDRD)	61.8 +/- 43.5<br>60.7 +/- 34.2	1,363<br>1,343	91.6%<br>90.3%	0.457	0.029
1<br>2		0 - 0 mL/min/{1.73_m2}		1,369<br>1,344	92.0%<br>90.3%	0.106	0.059

Table 9. Baseline characteristics of study subjects in baseline LVEF=40-50% (before and after Propensity score matching). (cohort 1, purple, midodrine group; cohort 2, green: non-midodrine group)

Cohort 1 and cohort 2 patient count before and after propensity score matching

Cohort	Patient count before matching	Patient count after matching
1 - M+; EF40-50	693	419
2 - M- EF 40-50	477	419

Propensity score density function - Before and after matching (cohort 1 - purple, cohort 2 - green)

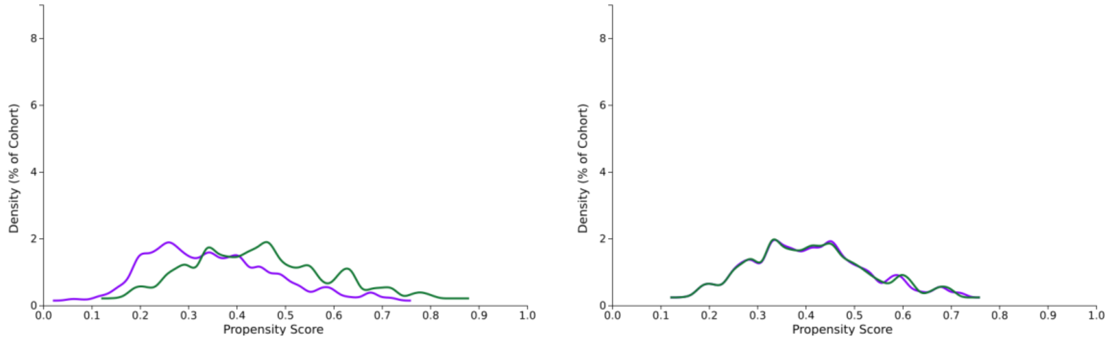

Cohort 1 (N = 693) and cohort 2 (N = 477) characteristics before propensity score matching

Demographics

Cohort			Mean ± SD	Patients	% of Cohort	P-Value	Std diff.
1	AI	Age at Index	65.9 +/- 13.0	693	100%	<0.001	0.215
2			63.1 +/- 13.0	477	100%		
1	2106-3	White		523	75.5%	0.279	0.065
2				373	78.2%		
1	2054-5	Black or African American		122	17.6%	0.925	0.006
2				85	17.8%		
1	M	Male		425	61.3%	0.067	0.109
2				267	56.0%		
1	2028-9	Asian		10	1.4%	0.397	0.050
2				10	2.1%		

Diagnosis

Cohort			Mean ± SD	Patients	% of Cohort	P-Value	Std diff.
1	I20-I25	Ischemic heart diseases		536	77.3%	0.028	0.130
2				342	71.7%		
1	E08-E13	Diabetes mellitus		301	43.4%	0.043	0.121
2				179	37.5%		
1	I42	Cardiomyopathy		368	53.1%	0.687	0.024

Cohort			Mean ± SD	Patients	% of Cohort	P-Value	Std diff.
2				259	54.3%		
1<br>2	J44	Other chronic obstructive pulmonary disease		216<br>133	31.2%<br>27.9%	0.227	0.072

Medication

Cohort			Mean ± SD	Patients	% of Cohort	P-Value	Std diff.
1<br>2	CV100	BETA BLOCKERS/RELATED		583<br>395	84.1%<br>82.8%	0.550	0.035
1<br>2	1649480	ivabradine		10<br>10	1.4%<br>2.1%	0.397	0.050
1<br>2	CV805	ANGIOTENSIN II INHIBITOR		195<br>137	28.1%<br>28.7%	0.828	0.013
1<br>2	CV800	ACE INHIBITORS		202<br>197	29.1%<br>41.3%	<0.001	0.256
1<br>2	1545653	empagliflozin		47<br>34	6.8%<br>7.1%	0.819	0.014
1<br>2	1488564	dapagliflozin		44<br>32	6.3%<br>6.7%	0.806	0.015
1<br>2	1373458	canagliflozin		10<br>10	1.4%<br>2.1%	0.397	0.050
1<br>2	9997	spironolactone		264<br>183	38.1%<br>38.4%	0.926	0.006
1<br>2	298869	eplerenone		15<br>15	2.2%<br>3.1%	0.297	0.061

Laboratory

Cohort			Mean ± SD	Patients	% of Cohort	P-Value	Std diff.
1<br>2	9072	Natriuretic peptide.B prohormone N-Terminal [Mass/volume] in Serum, Plasma or Blood	9662.5 +/- 12453.5<br>7265.6 +/- 10927.5	323<br>176	46.6%<br>36.9%	0.033	0.205
1<br>2		0 - 0 pg/mL		324<br>176	46.8%<br>36.9%	0.001	0.201
1<br>2	2003	Left Ventricular Ejection Fraction (LVEF) (%)	42.8 +/- 13.0<br>42.5 +/- 9.8	593<br>350	85.6%<br>73.4%	0.779	0.020
1<br>2		0 - 0 %		593<br>350	85.6%<br>73.4%	<0.001	0.305

1<br>2	9085	Blood Pressure, Systolic	88.2 +/- 22.0<br>97.4 +/- 19.3	683<br>475	98.6%<br>99.6%	<0.001	0.448
1<br>2		0 - 0 mm[Hg]		683<br>475	98.6%<br>99.6%	0.088	0.107
1<br>2	8001	Glomerular filtration rate/1.73 sq M.predicted [Volume Rate/Area] in Serum, Plasma or Blood by Creatinine-based formula (MDRD)	60.3 +/- 41.2<br>59.7 +/- 27.5	688<br>468	99.3%<br>98.1%	0.811	0.015
1<br>2		0 - 0 mL/min/{1.73_m2}		688<br>468	99.3%<br>98.1%	0.072	0.103

Cohort 1 (N = 419) and cohort 2 (N = 419) characteristics after propensity score matching

Demographics

Cohort			Mean ± SD	Patients	% of Cohort	P-Value	Std diff.
1<br>2	AI	Age at Index	63.8 +/- 13.5<br>64.1 +/- 12.7	419<br>419	100%<br>100%	0.786	0.019
1<br>2	2106-3	White		328<br>324	78.3%<br>77.3%	0.740	0.023
1<br>2	2054-5	Black or African American		76<br>77	18.1%<br>18.4%	0.929	0.006
1<br>2	M	Male		246<br>236	58.7%<br>56.3%	0.485	0.048
1<br>2	2028-9	Asian		10<br>10	2.4%<br>2.4%	1	<0.001

Diagnosis

Cohort			Mean ± SD	Patients	% of Cohort	P-Value	Std diff.
1<br>2	I20-I25	Ischemic heart diseases		308<br>309	73.5%<br>73.7%	0.938	0.005
1<br>2	E08-E13	Diabetes mellitus		163<br>164	38.9%<br>39.1%	0.944	0.005
1<br>2	I42	Cardiomyopathy		234<br>227	55.8%<br>54.2%	0.627	0.034
1<br>2	J44	Other chronic obstructive pulmonary disease		122<br>121	29.1%<br>28.9%	0.939	0.005

Medication								
Cohort			Mean ± SD	Patients	% of Cohort	P-Value	Std diff.
1<br>2	CV100	BETA BLOCKERS/RELATED		348<br>354	83.1%<br>84.5%	0.574	0.039
1<br>2	1649480	ivabradine		10<br>10	2.4%<br>2.4%	1	<0.001
1<br>2	CV805	ANGIOTENSIN II INHIBITOR		115<br>116	27.4%<br>27.7%	0.938	0.005
1<br>2	CV800	ACE INHIBITORS		166<br>161	39.6%<br>38.4%	0.723	0.024
1<br>2	1545653	empagliflozin		25<br>28	6.0%<br>6.7%	0.670	0.029
1<br>2	1488564	dapagliflozin		27<br>25	6.4%<br>6.0%	0.775	0.020
1<br>2	1373458	canagliflozin		0<br>10	0%<br>2.4%	0.001	0.221
1<br>2	9997	spironolactone		167<br>158	39.9%<br>37.7%	0.523	0.044
1<br>2	298869	eplerenone		10<br>11	2.4%<br>2.6%	0.825	0.015

Laboratory							
Cohort			Mean ± SD	Patients	% of Cohort	P-Value	Std diff.
1<br>2	9072	Natriuretic peptide.B prohormone N-Terminal [Mass/volume] in Serum, Plasma or Blood	7540.8 +/- 9457.0<br>7521.3 +/- 11186.9	150<br>166	35.8%<br>39.6%	0.987	0.002
1<br>2		0 - 0 pg/mL		151<br>166	36.0%<br>39.6%	0.285	0.074
1<br>2	2003	Left Ventricular Ejection Fraction (LVEF) (%)	43.0 +/- 12.9<br>42.8 +/- 9.7	331<br>323	79.0%<br>77.1%	0.862	0.014
1<br>2		0 - 0 %		331<br>323	79.0%<br>77.1%	0.504	0.046
1<br>2	9085	Blood Pressure, Systolic	88.6 +/- 21.9<br>97.5 +/- 19.7	417<br>417	99.5%<br>99.5%	<0.001	0.426
1		0 - 0 mm[Hg]		417	99.5%	1	<0.001

2				417	99.5%		
1<br>2	8001	Glomerular filtration rate/1.73 sq M.predicted [Volume Rate/Area] in Serum, Plasma or Blood by Creatinine-based formula (MDRD)	62.9 +/- 43.3<br>60.3 +/- 27.9	415<br>415	99.0%<br>99.0%	0.311	0.070
1<br>2		0 - 0 mL/min/{1.73_m2}		415<br>415	99.0%<br>99.0%	1	<0.001

Table 10. Baseline characteristics of study subjects in baseline LVEF=30-40% (before and after Propensity score matching). (cohort 1, purple, midodrine group; cohort 2, green: non-midodrine group)

Cohort 1 and cohort 2 patient count before and after propensity score matching

Cohort	Patient count before matching	Patient count after matching
1 - M+; EF30-40	739	495
2 - M- EF 30-40	610	495

Propensity score density function - Before and after matching (cohort 1 - purple, cohort 2 - green)

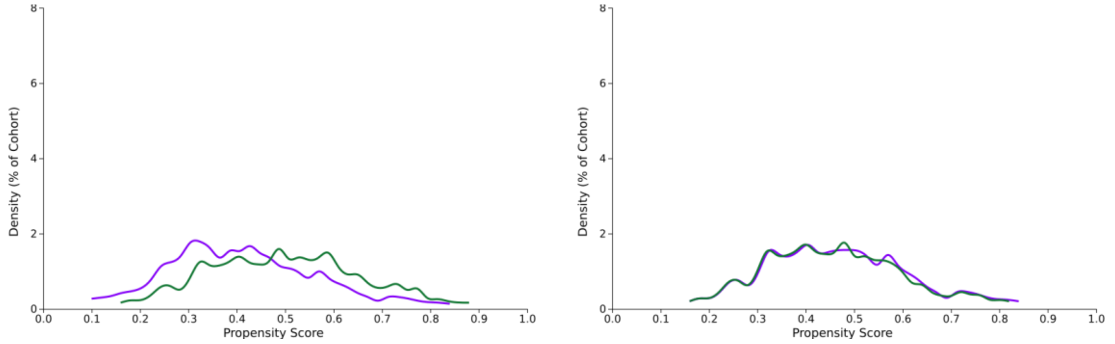

Cohort 1 (N = 739) and cohort 2 (N = 610) characteristics before propensity score matching

Demographics

Cohort			Mean ± SD	Patients	% of Cohort	P-Value	Std diff.
1	AI	Age at Index	66.1 +/- 12.7	739	100%	<0.001	0.254
2			62.8 +/- 12.9	610	100%		
1	2106-3	White		537	72.7%	0.879	0.008
2				441	72.3%		
1	2054-5	Black or African American		132	17.9%	0.042	0.111
2				136	22.3%		
1	M	Male		491	66.4%	0.305	0.056
2				389	63.8%		
1	2028-9	Asian		11	1.5%	0.824	0.012
2				10	1.6%		

Diagnosis

Cohort			Mean ± SD	Patients	% of Cohort	P-Value	Std diff.
1	I20-I25	Ischemic heart diseases		610	82.5%	0.037	0.114
2				476	78.0%		
1	E08-E13	Diabetes mellitus		356	48.2%	0.012	0.138
2				252	41.3%		
1	I42	Cardiomyopathy		449	60.8%	0.650	0.025

Cohort			Mean ± SD	Patients	% of Cohort	P-Value	Std diff.
2				378	62.0%		
1<br>2	J44	Other chronic obstructive pulmonary disease		235<br>163	31.8%<br>26.7%	0.042	0.112

Medication

Cohort			Mean ± SD	Patients	% of Cohort	P-Value	Std diff.
1<br>2	CV100	BETA BLOCKERS/RELATED		637<br>521	86.2%<br>85.4%	0.680	0.023
1<br>2	1649480	ivabradine		10<br>13	1.4%<br>2.1%	0.272	0.059
1<br>2	CV805	ANGIOTENSIN II INHIBITOR		251<br>222	34.0%<br>36.4%	0.352	0.051
1<br>2	CV800	ACE INHIBITORS		256<br>295	34.6%<br>48.4%	<0.001	0.281
1<br>2	1545653	empagliflozin		64<br>52	8.7%<br>8.5%	0.929	0.005
1<br>2	1488564	dapagliflozin		55<br>51	7.4%<br>8.4%	0.533	0.034
1<br>2	1373458	canagliflozin		10<br>10	1.4%<br>1.6%	0.665	0.024
1<br>2	9997	spironolactone		303<br>276	41.0%<br>45.2%	0.117	0.086
1<br>2	298869	eplerenone		21<br>17	2.8%<br>2.8%	0.952	0.003

Laboratory

Cohort			Mean ± SD	Patients	% of Cohort	P-Value	Std diff.
1<br>2	9072	Natriuretic peptide.B prohormone N-Terminal [Mass/volume] in Serum, Plasma or Blood	9818.0 +/- 11492.4<br>8072.8 +/- 11028.6	351<br>241	47.5%<br>39.5%	0.066	0.155
1<br>2		0 - 0 pg/mL		351<br>241	47.5%<br>39.5%	0.003	0.162
1<br>2	2003	Left Ventricular Ejection Fraction (LVEF) (%)	36.4 +/- 13.2<br>33.5 +/- 9.1	657<br>471	88.9%<br>77.2%	<0.001	0.250
1<br>2		0 - 0 %		657<br>471	88.9%<br>77.2%	<0.001	0.316

1<br>2	9085	Blood Pressure, Systolic	86.5 +/- 21.8<br>95.5 +/- 20.2	731<br>608	98.9%<br>99.7%	<0.001	0.428
1<br>2		0 - 0 mm[Hg]		731<br>608	98.9%<br>99.7%	0.108	0.090
1<br>2	8001	Glomerular filtration rate/1.73 sq M.predicted [Volume Rate/Area] in Serum, Plasma or Blood by Creatinine-based formula (MDRD)	57.3 +/- 39.2<br>60.7 +/- 29.8	730<br>604	98.8%<br>99.0%	0.079	0.098
1<br>2		0 - 0 mL/min/{1.73_m2}		730<br>604	98.8%<br>99.0%	0.683	0.022

Cohort 1 (N = 495) and cohort 2 (N = 495) characteristics after propensity score matching

Demographics

Cohort			Mean ± SD	Patients	% of Cohort	P-Value	Std diff.
1<br>2	AI	Age at Index	63.9 +/- 13.3<br>63.8 +/- 12.4	495<br>495	100%<br>100%	0.896	0.008
1<br>2	2106-3	White		359<br>357	72.5%<br>72.1%	0.887	0.009
1<br>2	2054-5	Black or African American		103<br>107	20.8%<br>21.6%	0.756	0.020
1<br>2	M	Male		323<br>318	65.3%<br>64.2%	0.739	0.021
1<br>2	2028-9	Asian		10<br>10	2.0%<br>2.0%	1	<0.001

Diagnosis

Cohort			Mean ± SD	Patients	% of Cohort	P-Value	Std diff.
1<br>2	I20-I25	Ischemic heart diseases		396<br>393	80%<br>79.4%	0.813	0.015
1<br>2	E08-E13	Diabetes mellitus		215<br>221	43.4%<br>44.6%	0.701	0.024
1<br>2	I42	Cardiomyopathy		300<br>305	60.6%<br>61.6%	0.744	0.021
1<br>2	J44	Other chronic obstructive pulmonary disease		139<br>144	28.1%<br>29.1%	0.725	0.022

Medication								
Cohort			Mean ± SD	Patients	% of Cohort	P-Value	Std diff.
1<br>2	CV100	BETA BLOCKERS/RELATED		425<br>427	85.9%<br>86.3%	0.854	0.012
1<br>2	1649480	ivabradine		10<br>10	2.0%<br>2.0%	1	<0.001
1<br>2	CV805	ANGIOTENSIN II INHIBITOR		177<br>181	35.8%<br>36.6%	0.791	0.017
1<br>2	CV800	ACE INHIBITORS		219<br>221	44.2%<br>44.6%	0.898	0.008
1<br>2	1545653	empagliflozin		39<br>44	7.9%<br>8.9%	0.566	0.036
1<br>2	1488564	dapagliflozin		35<br>38	7.1%<br>7.7%	0.715	0.023
1<br>2	1373458	canagliflozin		10<br>0	2.0%<br>0%	0.001	0.203
1<br>2	9997	spironolactone		211<br>216	42.6%<br>43.6%	0.748	0.020
1<br>2	298869	eplerenone		17<br>12	3.4%<br>2.4%	0.346	0.060

Laboratory							
Cohort			Mean ± SD	Patients	% of Cohort	P-Value	Std diff.
1<br>2	9072	Natriuretic peptide.B prohormone N-Terminal [Mass/volume] in Serum, Plasma or Blood	10252.6 +/- 12381.9<br>8594.0 +/- 11477.0	214<br>208	43.2%<br>42.0%	0.155	0.139
1<br>2		0 - 0 pg/mL		214<br>208	43.2%<br>42.0%	0.700	0.025
1<br>2	2003	Left Ventricular Ejection Fraction (LVEF) (%)	36.5 +/- 13.5<br>33.7 +/- 9.1	417<br>416	84.2%<br>84.0%	<0.001	0.243
1<br>2		0 - 0 %		417<br>416	84.2%<br>84.0%	0.931	0.006
1<br>2	9085	Blood Pressure, Systolic	88.6 +/- 21.0<br>94.8 +/- 19.5	494<br>493	99.8%<br>99.6%	<0.001	0.305
1		0 - 0 mm[Hg]		494	99.8%	0.563	0.037

2				493	99.6%		
1<br>2	8001	Glomerular filtration rate/1.73 sq M.predicted [Volume Rate/Area] in Serum, Plasma or Blood by Creatinine-based formula (MDRD)	60.1 +/- 41.8<br>59.2 +/- 30.5	490<br>489	99.0%<br>98.8%	0.712	0.024
1<br>2		0 - 0 mL/min/{1.73_m2}		490<br>489	99.0%<br>98.8%	0.762	0.019

Table 11. Baseline characteristics of study subjects in baseline LVEF=20-30% (before and after Propensity score matching). (cohort 1, purple, midodrine group; cohort 2, green: non-midodrine group)

Cohort 1 and cohort 2 patient count before and after propensity score matching

Cohort	Patient count before matching	Patient count after matching
1 - M+; EF20-30	738	666
2 - M- EF 20-30	992	666

Propensity score density function - Before and after matching (cohort 1 - purple, cohort 2 - green)

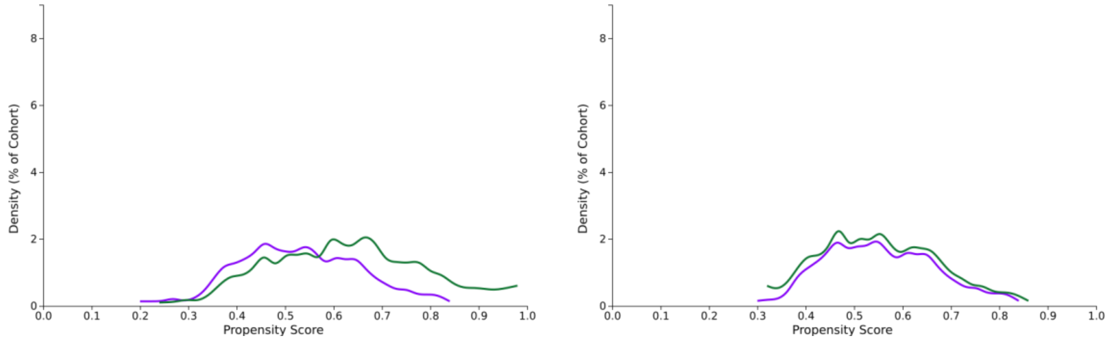

Cohort 1 (N = 738) and cohort 2 (N = 992) characteristics before propensity score matching

Demographics

Cohort			Mean ± SD	Patients	% of Cohort	P-Value	Std diff.
1	AI	Age at Index	64.6 +/- 12.8	738	100%	<0.001	0.217
2			61.8 +/- 13.1	992	100%		
1	2106-3	White		515	69.8%	0.041	0.100
2				646	65.1%		
1	2054-5	Black or African American		154	20.9%	0.001	0.158
2				274	27.6%		
1	M	Male		525	71.1%	0.534	0.030
2				692	69.8%		
1	2028-9	Asian		10	1.4%	0.644	0.022
2				11	1.1%		

Diagnosis

Cohort			Mean ± SD	Patients	% of Cohort	P-Value	Std diff.
1	I20-I25	Ischemic heart diseases		604	81.8%	0.011	0.124
2				762	76.8%		
1	E08-E13	Diabetes mellitus		374	50.7%	0.017	0.117
2				445	44.9%		
1	I42	Cardiomyopathy		525	71.1%	0.029	0.106

Cohort	Code	Description	Mean ± SD	Patients	% of Cohort	P-Value	Std diff.
2				752	75.8%		
1<br>2	J44	Other chronic obstructive pulmonary disease		213<br>263	28.9%<br>26.5%	0.279	0.053

Medication

Cohort			Mean ± SD	Patients	% of Cohort	P-Value	Std diff.
1<br>2	CV100	BETA BLOCKERS/RELATED		626<br>857	84.8%<br>86.4%	0.357	0.045
1<br>2	1649480	ivabradine		10<br>14	1.4%<br>1.4%	0.921	0.005
1<br>2	CV805	ANGIOTENSIN II INHIBITOR		297<br>381	40.2%<br>38.4%	0.439	0.038
1<br>2	CV800	ACE INHIBITORS		242<br>460	32.8%<br>46.4%	<0.001	0.280
1<br>2	1545653	empagliflozin		71<br>82	9.6%<br>8.3%	0.326	0.047
1<br>2	1488564	dapagliflozin		59<br>73	8.0%<br>7.4%	0.622	0.024
1<br>2	1373458	canagliflozin		0<br>10	0%<br>1.0%	0.006	0.143
1<br>2	9997	spironolactone		347<br>547	47.0%<br>55.1%	0.001	0.163
1<br>2	298869	eplerenone		29<br>39	3.9%<br>3.9%	0.998	<0.001

Laboratory

Cohort			Mean ± SD	Patients	% of Cohort	P-Value	Std diff.
1<br>2	9072	Natriuretic peptide.B prohormone N-Terminal [Mass/volume] in Serum, Plasma or Blood	10480.5 +/- 12529.1<br>8528.8 +/- 11257.5	355<br>436	48.1%<br>44.0%	0.021	0.164
1<br>2		0 - 0 pg/mL		356<br>436	48.2%<br>44.0%	0.077	0.086
1<br>2	2003	Left Ventricular Ejection Fraction (LVEF) (%)	29.0 +/- 13.1<br>24.4 +/- 8.3	653<br>803	88.5%<br>80.9%	<0.001	0.418
1<br>2		0 - 0 %		653<br>803	88.5%<br>80.9%	<0.001	0.211

Cohort			Mean ± SD	Patients	% of Cohort	P-Value	Std diff.
1<br>2	9085	Blood Pressure, Systolic	83.9 +/- 22.1<br>89.6 +/- 20.1	731<br>989	99.1%<br>99.7%	<0.001	0.269
1<br>2		0 - 0 mm[Hg]		731<br>989	99.1%<br>99.7%	0.080	0.082
1<br>2	8001	Glomerular filtration rate/1.73 sq M.predicted [Volume Rate/Area] in Serum, Plasma or Blood by Creatinine-based formula (MDRD)	55.6 +/- 37.4<br>56.6 +/- 26.6	730<br>982	98.9%<br>99.0%	0.527	0.030
1<br>2		0 - 0 mL/min/{1.73_m2}		732<br>983	99.2%<br>99.1%	0.834	0.010

Cohort 1 (N = 666) and cohort 2 (N = 666) characteristics after propensity score matching

Demographics

Cohort			Mean ± SD	Patients	% of Cohort	P-Value	Std diff.
1<br>2	AI	Age at Index	63.9 +/- 12.8<br>63.9 +/- 11.8	666<br>666	100%<br>100%	0.982	0.001
1<br>2	2106-3	White		466<br>448	70.0%<br>67.3%	0.288	0.058
1<br>2	2054-5	Black or African American		147<br>160	22.1%<br>24.0%	0.398	0.046
1<br>2	M	Male		470<br>460	70.6%<br>69.1%	0.551	0.033
1<br>2	2028-9	Asian		10<br>10	1.5%<br>1.5%	1	<0.001

Diagnosis

Cohort			Mean ± SD	Patients	% of Cohort	P-Value	Std diff.
1<br>2	I20-I25	Ischemic heart diseases		537<br>535	80.6%<br>80.3%	0.890	0.008
1<br>2	E08-E13	Diabetes mellitus		325<br>325	48.8%<br>48.8%	1	<0.001
1<br>2	I42	Cardiomyopathy		484<br>489	72.7%<br>73.4%	0.758	0.017
1<br>2	J44	Other chronic obstructive pulmonary disease		184<br>187	27.6%<br>28.1%	0.855	0.010

Medication							
Cohort			Mean ± SD	Patients	% of Cohort	P-Value	Std diff.
1<br>2	CV100	BETA BLOCKERS/RELATED		565<br>572	84.8%<br>85.9%	0.587	0.030
1<br>2	1649480	ivabradine		10<br>10	1.5%<br>1.5%	1	<0.001
1<br>2	CV805	ANGIOTENSIN II INHIBITOR		269<br>274	40.4%<br>41.1%	0.780	0.015
1<br>2	CV800	ACE INHIBITORS		238<br>238	35.7%<br>35.7%	1	<0.001
1<br>2	1545653	empagliflozin		60<br>65	9.0%<br>9.8%	0.638	0.026
1<br>2	1488564	dapagliflozin		45<br>53	6.8%<br>8.0%	0.401	0.046
1<br>2	1373458	canagliflozin		0<br>0	0%<br>0%	--	--
1<br>2	9997	spironolactone		329<br>325	49.4%<br>48.8%	0.826	0.012
1<br>2	298869	eplerenone		27<br>25	4.1%<br>3.8%	0.777	0.016

Laboratory							
Cohort			Mean ± SD	Patients	% of Cohort	P-Value	Std diff.
1<br>2	9072	Natriuretic peptide.B prohormone N-Terminal [Mass/volume] in Serum, Plasma or Blood	9935.5 +/- 11548.1<br>9090.6 +/- 11830.7	311<br>319	46.7%<br>47.9%	0.365	0.072
1<br>2		0 - 0 pg/mL		312<br>319	46.8%<br>47.9%	0.701	0.021
1<br>2	2003	Left Ventricular Ejection Fraction (LVEF) (%)	28.6 +/- 13.1<br>24.5 +/- 8.2	582<br>581	87.4%<br>87.2%	<0.001	0.385
1<br>2		0 - 0 %		582<br>581	87.4%<br>87.2%	0.934	0.005
1<br>2	9085	Blood Pressure, Systolic	83.8 +/- 22.2<br>89.0 +/- 21.0	665<br>665	99.8%<br>99.8%	<0.001	0.241
1		0 - 0 mm[Hg]		665	99.8%	1	<0.001

2				665	99.8%		
1<br>2	8001	Glomerular filtration rate/1.73 sq M.predicted [Volume Rate/Area] in Serum, Plasma or Blood by Creatinine-based formula (MDRD)	56.6 +/- 37.6<br>54.0 +/- 25.7	659<br>660	98.9%<br>99.1%	0.142	0.081
1<br>2		0 - 0 mL/min/{1.73_m2}		661<br>660	99.2%<br>99.1%	0.762	0.017

Table 12. Baseline characteristics of study subjects in baseline LVEF<20% (before and after Propensity score matching). (cohort 1, purple, midodrine group; cohort 2, green: non-midodrine group)

Cohort 1 and cohort 2 patient count before and after propensity score matching

Cohort	Patient count before matching	Patient count after matching
1 - M-+EF<20%	488	469
2 - M-EF<20%	1,473	469

Propensity score density function - Before and after matching (cohort 1 - purple, cohort 2 - green)

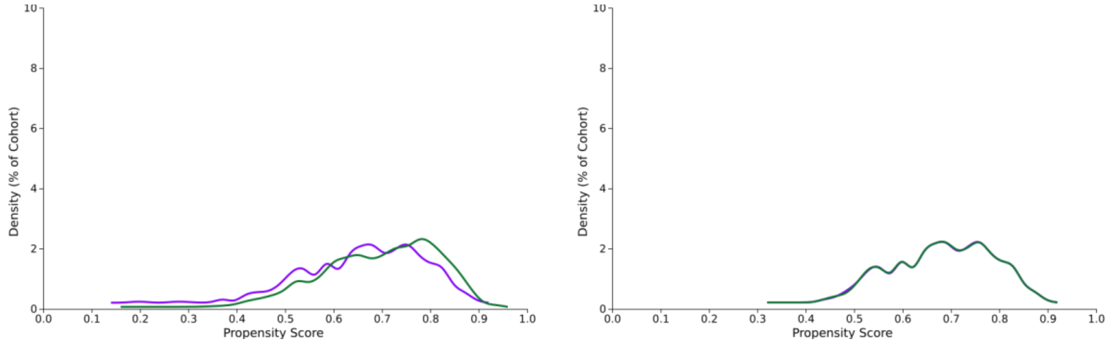

Cohort 1 (N = 488) and cohort 2 (N = 1,473) characteristics before propensity score matching

Demographics

Cohort			Mean ± SD	Patients	% of Cohort	P-Value	Std diff.
1	AI	Age at Index	62.5 +/- 13.1	488	100%	<0.001	0.297
2			58.5 +/- 13.5	1,473	100%		
1	2106-3	White		317	65.0%	0.083	0.091
2				892	60.6%		
1	2054-5	Black or African American		128	26.2%	0.014	0.131
2				474	32.2%		
1	M	Male		353	72.3%	0.537	0.032
2				1,044	70.9%		
1	2028-9	Asian		10	2.0%	0.025	0.104
2				12	0.8%		

Diagnosis

Cohort			Mean ± SD	Patients	% of Cohort	P-Value	Std diff.
1	I20-I25	Ischemic heart diseases		389	79.7%	0.018	0.126
2				1,096	74.4%		
1	E08-E13	Diabetes mellitus		235	48.2%	0.115	0.082
2				649	44.1%		
1	I42	Cardiomyopathy		384	78.7%	0.077	0.091

Cohort			Mean ± SD	Patients	% of Cohort	P-Value	Std diff.
2				1,212	82.3%		
1<br>2	J44	Other chronic obstructive pulmonary disease		142<br>351	29.1%<br>23.8%	0.020	0.120

Medication

Cohort			Mean ± SD	Patients	% of Cohort	P-Value	Std diff.
1<br>2	CV100	BETA BLOCKERS/RELATED		399<br>1,278	81.8%<br>86.8%	0.007	0.138
1<br>2	1649480	ivabradine		10<br>33	2.0%<br>2.2%	0.803	0.013
1<br>2	CV805	ANGIOTENSIN II INHIBITOR		206<br>629	42.2%<br>42.7%	0.850	0.010
1<br>2	CV800	ACE INHIBITORS		168<br>721	34.4%<br>48.9%	<0.001	0.298
1<br>2	1545653	empagliflozin		52<br>121	10.7%<br>8.2%	0.099	0.084
1<br>2	1488564	dapagliflozin		42<br>105	8.6%<br>7.1%	0.282	0.055
1<br>2	1373458	canagliflozin		10<br>10	2.0%<br>0.7%	0.009	0.118
1<br>2	9997	spironolactone		240<br>918	49.2%<br>62.3%	<0.001	0.267
1<br>2	298869	eplerenone		23<br>80	4.7%<br>5.4%	0.538	0.033

Laboratory

Cohort			Mean ± SD	Patients	% of Cohort	P-Value	Std diff.
1<br>2	9072	Natriuretic peptide.B prohormone N-Terminal [Mass/volume] in Serum, Plasma or Blood	10493.4 +/- 11805.7<br>7845.5 +/- 10596.6	210<br>604	43.0%<br>41.0%	0.003	0.236
1<br>2		0 - 0 pg/mL		210<br>604	43.0%<br>41.0%	0.431	0.041
1<br>2	2003	Left Ventricular Ejection Fraction (LVEF) (%)	23.3 +/- 13.2<br>20.4 +/- 9.8	417<br>1,238	85.5%<br>84.0%	<0.001	0.248
1<br>2		0 - 0 %		417<br>1,238	85.5%<br>84.0%	0.459	0.039

1<br>2	9085	Blood Pressure, Systolic	83.2 +/- 21.6<br>88.4 +/- 20.5	478<br>1,469	98.0%<br>99.7%	<0.001	0.246
1<br>2		0 - 0 mm[Hg]		478<br>1,469	98.0%<br>99.7%	<0.001	0.167
1<br>2	8001	Glomerular filtration rate/1.73 sq M.predicted [Volume Rate/Area] in Serum, Plasma or Blood by Creatinine-based formula (MDRD)	56.0 +/- 37.2<br>58.9 +/- 26.6	483<br>1,464	99.0%<br>99.4%	0.062	0.090
1<br>2		0 - 0 mL/min/{1.73_m2}		484<br>1,464	99.2%<br>99.4%	0.622	0.025

Cohort 1 (N = 469) and cohort 2 (N = 469) characteristics after propensity score matching

Demographics

Cohort			Mean ± SD	Patients	% of Cohort	P-Value	Std diff.
1<br>2	AI	Age at Index	62.1 +/- 13.1<br>62.8 +/- 12.2	469<br>469	100%<br>100%	0.384	0.057
1<br>2	2106-3	White		301<br>285	64.2%<br>60.8%	0.281	0.071
1<br>2	2054-5	Black or African American		127<br>140	27.1%<br>29.9%	0.347	0.061
1<br>2	M	Male		336<br>330	71.6%<br>70.4%	0.666	0.028
1<br>2	2028-9	Asian		10<br>10	2.1%<br>2.1%	1	<0.001

Diagnosis

Cohort			Mean ± SD	Patients	% of Cohort	P-Value	Std diff.
1<br>2	I20-I25	Ischemic heart diseases		372<br>372	79.3%<br>79.3%	1	<0.001
1<br>2	E08-E13	Diabetes mellitus		223<br>203	47.5%<br>43.3%	0.190	0.086
1<br>2	I42	Cardiomyopathy		370<br>372	78.9%<br>79.3%	0.872	0.010
1<br>2	J44	Other chronic obstructive pulmonary disease		132<br>120	28.1%<br>25.6%	0.377	0.058

Medication							
Cohort			Mean ± SD	Patients	% of Cohort	P-Value	Std diff.
1<br>2	CV100	BETA BLOCKERS/RELATED		387<br>383	82.5%<br>81.7%	0.733	0.022
1<br>2	1649480	ivabradine		10<br>10	2.1%<br>2.1%	1	<0.001
1<br>2	CV805	ANGIOTENSIN II INHIBITOR		201<br>187	42.9%<br>39.9%	0.353	0.061
1<br>2	CV800	ACE INHIBITORS		167<br>164	35.6%<br>35.0%	0.838	0.013
1<br>2	1545653	empagliflozin		49<br>53	10.4%<br>11.3%	0.675	0.027
1<br>2	1488564	dapagliflozin		37<br>35	7.9%<br>7.5%	0.806	0.016
1<br>2	1373458	canagliflozin		10<br>0	2.1%<br>0%	0.001	0.209
1<br>2	9997	spironolactone		233<br>246	49.7%<br>52.5%	0.396	0.055
1<br>2	298869	eplerenone		23<br>23	4.9%<br>4.9%	1	<0.001

Laboratory							
Cohort			Mean ± SD	Patients	% of Cohort	P-Value	Std diff.
1<br>2	9072	Natriuretic peptide.B prohormone N-Terminal [Mass/volume] in Serum, Plasma or Blood	10635.6 +/- 11907.4<br>8528.5 +/- 10446.5	205<br>192	43.7%<br>40.9%	0.062	0.188
1<br>2		0 - 0 pg/mL		205<br>192	43.7%<br>40.9%	0.390	0.056
1<br>2	2003	Left Ventricular Ejection Fraction (LVEF) (%)	22.9 +/- 12.7<br>20.7 +/- 10.3	399<br>400	85.1%<br>85.3%	0.006	0.194
1<br>2		0 - 0 %		399<br>400	85.1%<br>85.3%	0.927	0.006
1<br>2	9085	Blood Pressure, Systolic	83.0 +/- 21.7<br>87.8 +/- 21.5	467<br>467	99.6%<br>99.6%	0.001	0.222
1		0 - 0 mm[Hg]		467	99.6%	1	<0.001

2				467	99.6%		
1<br>2	8001	Glomerular filtration rate/1.73 sq M.predicted [Volume Rate/Area] in Serum, Plasma or Blood by Creatinine-based formula (MDRD)	55.6 +/- 35.7<br>54.8 +/- 25.1	464<br>464	98.9%<br>98.9%	0.694	0.026
1<br>2		0 - 0 mL/min/{1.73_m2}		465<br>464	99.1%<br>98.9%	0.738	0.022

Table 13. Incidence of outcomes among midodrine and non-midodrine groups (after prosperity score matching) stratified by genders.

Outcome	Hazard ratio (95% with CI)	
	Male	Female
CKD, stage 4	0.975 (0.813, 1.169)	0.948 (0.745, 1.207)
CKD, stage 5	1.632 (0.979, 2.719)	1.160 (0.626, 2.149)
Acute pulmonary edema	0.905 (0.741, 1.107)	1.278 (0.994, 1.644)
Respiratory failure	**1.169 (1.065, 1.283)**	**1.297 (1.146, 1.468)**
Stay in intensive care unit	**1.170 (1.065, 1.285)**	**1.144 (1.009, 1.297)**
Emergency room visit	**0.797 (0.723, 0.879)**	**0.687 (0.605, 0.781)**
All hospitalization	**1.161 (1.052, 1.282)**	**1.231 (1.075, 1.409)**
Cardiac arrest	0.959 (0.807, 1.138)	**1.457 (1.137, 1.867)**
All-cause mortality	1.030 (0.940, 1.129)	1.132 (0.999, 1.283)

Table 14. Incidence of outcomes among midodrine and non-midodrine (after prosperity score matching) stratified by age (18-65y/o vs. ≥65y/o)

Outcome	Hazard ratio (95% with CI)	
	Young	The elderly
CKD, stage 4	1.307 (0.995, 1.716)	1.025 (0.870, 1.208)
CKD, stage 5	0.944 (0.485, 1.837)	**1.553 (1.000, 2.412)**
Acute pulmonary edema	0.992 (0.776, 1.268)	**1.264 (1.043, 1.532)**
Respiratory failure	**1.187 (1.050, 1.342)**	**1.467 (1.337, 1.609)**
Stay in intensive care unit	1.114 (0.985, 1.260)	**1.647 (1.498, 1.809)**
Emergency room visit	**0.777 (0.685, 0.881)**	1.016 (0.923, 1.118)
All hospitalization	1.110 (0.970, 1.271)	**1.810 (1.634, 2.004)**
Cardiac arrest	1.152 (0.935, 1.419)	1.169 (0.976, 1.401)
All-cause mortality	**1.196 (1.053, 1.360)**	1.087 (0.992, 1.191)

Table 15. Incidence of outcomes among midodrine and non-midodrine (after prosperity score matching) stratified by frequency of medication (at least a certain number of medication refills)

Outcome	Hazard ratio (95% with CI)			
	frequency of medication (at least a certain number of medication refills)			
	≥1	≥3	≥6	≥9
CKD, stage 4	1.003 (0.869, 1.157)	1.181 (0.994, 1.404)	1.199 (0.962, 1.493)	**1.750 (1.322, 2.316)**
CKD, stage 5	1.323 (0.904, 1.936)	**1.728 (1.053, 2.834)**	1.189 (0.610, 2.318)	1.202 (0.518, 2.790)
Acute pulmonary edema	0.952 (0.817, 1.110)	1.138 (0.939, 1.378)	**1.331 (1.035, 1.713)**	**1.569 (1.135, 2.168)**
Respiratory failure	**1.162 (1.080, 1.252)**	**1.271 (1.164, 1.388)**	**1.592 (1.416, 1.790)**	**1.889 (1.633, 2.185)**
Stay in intensive care unit	**1.141 (1.059, 1.230)**	**1.340 (1.226, 1.466)**	**1.438 (1.277, 1.619)**	**1.762 (1.522, 2.039)**
Emergency room visit	**0.767 (0.710, 0.829)**	0.916 (0.836, 1.003)	1.054 (0.935, 1.188)	**1.254 (1.083, 1.452)**
All hospitalization	**1.212 (1.119, 1.312)**	**1.432 (1.299, 1.578)**	**1.581 (1.385, 1.806)**	**1.815 (1.535, 2.147)**
Cardiac arrest	1.055 (0.917, 1.213)	**1.210 (1.021, 1.434)**	1.189 (0.952, 1.486)	**1.668 (1.257, 2.212)**
All-cause mortality	**1.090 (1.012, 1.173)**	1.091 (0.999, 1.192)	**1.148 (1.021, 1.291)**	**1.170 (1.011, 1.353)**

Table 16. Incidence of outcomes among midodrine and non-midodrine (after prosperity score matching) stratified baseline LVEF

Outcome	Hazard ratio (95% with CI)			
	LVEF (%)			
	40-50%	30-40%	20-30%	<20%
CKD, stage 4	**1.849 (1.109, 3.082)**	0.948 (0.603, 1.490)	1.370 (0.957, 1.963)	0.957 (0.634, 1.444)
CKD, stage 5	1 (0.412, 2.428)	1 (0.412, 2.424)	1.512 (0.674, 3.389)	1.102 (0.464, 2.621)
Acute pulmonary edema	1.055 (0.555, 2.007)	1.302 (0.726, 2.336)	1.311 (0.863, 1.992)	1.252 (0.786, 1.994)
Respiratory failure	**2.073 (1.570, 2.739)**	**1.695 (1.313, 2.187)**	**1.565 (1.255, 1.953)**	1.192 (0.919, 1.547)
Stay in intensive care unit	**2.536 (1.918, 3.353)**	**2.203 (1.708, 2.842)**	**1.977 (1.589, 2.460)**	**1.658 (1.278, 2.150)**
Emergency room visit	0.823 (0.622, 1.090)	**0.709 (0.548, 0.917)**	**0.776 (0.622, 0.968)**	**0.702 (0.541, 0.912)**
All hospitalization	**3.388 (2.315, 4.958)**	**3.276 (2.294, 4.677)**	**2.158 (1.552, 3.000)**	**2.048 (1.345, 3.121)**
Cardiac arrest	1.538 (0.940, 2.515)	1.026 (0.660, 1.592)	0.941 (0.634, 1.397)	0.889 (0.553, 1.430)
All-cause mortality	**1.342 (1.019, 1.766)**	1.216 (0.947, 1.561)	1.037 (0.836, 1.285)	**1.398 (1.080, 1.808)**

Table 17. Incidence of all outcomes among midodrine and non-midodrine groups (summary of all conditions)

Outcome	All	Gender		Age		Medication refill times				Baseline LVEF			
	All	Male	Female	Young	The elderly	≥1	≥3	≥6	≥9	40-50%	30-40%	20-30%	<20%
CKD, stage 4	1.003<br>(0.869, 1.157)	0.975<br>(0.813, 1.169)	0.948<br>(0.745, 1.207)	1.307<br>(0.995, 1.716)	1.025<br>(0.870, 1.208)	1.003<br>(0.869, 1.157)	1.181<br>(0.994, 1.404)	1.199<br>(0.962, 1.493)	**1.750**<br>**(1.322, 2.316)**	**1.849**<br>**(1.109, 3.082)**	0.948<br>(0.603, 1.490)	1.370<br>(0.957, 1.963)	0.957<br>(0.634, 1.444)
CKD, stage 5	1.323<br>(0.904, 1.936)	1.632<br>(0.979, 2.719)	1.160<br>(0.626, 2.149)	0.944<br>(0.485, 1.837)	**1.553**<br>**(1.000, 2.412)**	1.323<br>(0.904, 1.936)	**1.728**<br>**(1.053, 2.834)**	1.189<br>(0.610, 2.318)	1.202<br>(0.518, 2.790)	1<br>(0.412, 2.428)	1<br>(0.412, 2.424)	1.512<br>(0.674, 3.389)	1.102<br>(0.464, 2.621)
Acute pulmonary edema	0.952<br>(0.817, 1.110)	0.905<br>(0.741, 1.107)	1.278<br>(0.994, 1.644)	0.992<br>(0.776, 1.268)	**1.264**<br>**(1.043, 1.532)**	0.952<br>(0.817, 1.110)	1.138<br>(0.939, 1.378)	**1.331**<br>**(1.035, 1.713)**	**1.569**<br>**(1.135, 2.168)**	1.055<br>(0.555, 2.007)	1.302<br>(0.726, 2.336)	1.311<br>(0.863, 1.992)	1.252<br>(0.786, 1.994)
Respiratory failure	**1.162**<br>**(1.080, 1.252)**	**1.169**<br>**(1.065, 1.283)**	**1.297**<br>**(1.146, 1.468)**	**1.187**<br>**(1.050, 1.342)**	**1.467**<br>**(1.337, 1.609)**	**1.162**<br>**(1.080, 1.252)**	**1.271**<br>**(1.164, 1.388)**	**1.592**<br>**(1.416, 1.790)**	**1.889**<br>**(1.633, 2.185)**	**2.073**<br>**(1.570, 2.739)**	**1.695**<br>**(1.313, 2.187)**	**1.565**<br>**(1.255, 1.953)**	1.192<br>(0.919, 1.547)
Stay in intensive care unit	**1.141**<br>**(1.059, 1.230)**	**1.170**<br>**(1.065, 1.285)**	**1.144**<br>**(1.009, 1.297)**	1.114<br>(0.985, 1.260)	**1.647**<br>**(1.498, 1.809)**	**1.141**<br>**(1.059, 1.230)**	**1.340**<br>**(1.226, 1.466)**	**1.438**<br>**(1.277, 1.619)**	**1.762**<br>**(1.522, 2.039)**	**2.536**<br>**(1.918, 3.353)**	**2.203**<br>**(1.708, 2.842)**	**1.977**<br>**(1.589, 2.460)**	**1.658**<br>**(1.278, 2.150)**
Emergency room visit	**0.767**<br>**(0.710, 0.829)**	**0.797**<br>**(0.723, 0.879)**	**0.687**<br>**(0.605, 0.781)**	**0.777**<br>**(0.685, 0.881)**	1.016<br>(0.923, 1.118)	**0.767**<br>**(0.710, 0.829)**	0.916<br>(0.836, 1.003)	1.054<br>(0.935, 1.188)	**1.254**<br>**(1.083, 1.452)**	0.823<br>(0.622, 1.090)	**0.709**<br>**(0.548, 0.917)**	**0.776**<br>**(0.622, 0.968)**	**0.702**<br>**(0.541, 0.912)**
All hospitalization	**1.212**<br>**(1.119, 1.312)**	**1.161**<br>**(1.052, 1.282)**	**1.231**<br>**(1.075, 1.409)**	1.110<br>(0.970, 1.271)	**1.810**<br>**(1.634, 2.004)**	**1.212**<br>**(1.119, 1.312)**	**1.432**<br>**(1.299, 1.578)**	**1.581**<br>**(1.385, 1.806)**	**1.815**<br>**(1.535, 2.147)**	**3.388**<br>**(2.315, 4.958)**	**3.276**<br>**(2.294, 4.677)**	**2.158**<br>**(1.552, 3.000)**	**2.048**<br>**(1.345, 3.121)**
Cardiac arrest	1.055<br>(0.917, 1.213)	0.959<br>(0.807, 1.138)	**1.457**<br>**(1.137, 1.867)**	1.152<br>(0.935, 1.419)	1.169<br>(0.976, 1.401)	1.055<br>(0.917, 1.213)	**1.210**<br>**(1.021, 1.434)**	1.189<br>(0.952, 1.486)	**1.668**<br>**(1.257, 2.212)**	1.538<br>(0.940, 2.515)	1.026<br>(0.660, 1.592)	0.941<br>(0.634, 1.397)	0.889<br>(0.553, 1.430)
All-cause mortality	**1.090**<br>**(1.012, 1.173)**	1.030<br>(0.940, 1.129)	1.132<br>(0.999, 1.283)	**1.196**<br>**(1.053, 1.360)**	1.087<br>(0.992, 1.191)	**1.090**<br>**(1.012, 1.173)**	1.091<br>(0.999, 1.192)	**1.148**<br>**(1.021, 1.291)**	**1.170**<br>**(1.011, 1.353)**	**1.342**<br>**(1.019, 1.766)**	1.216<br>(0.947, 1.561)	1.037<br>(0.836, 1.285)	**1.398**<br>**(1.080, 1.808)**

Figure 1. Propensity score density function – before (A) and after (B) matching (cohort 1 -midodrine group (purple), cohort 2 -non-midodrine group (green))

A. Before matching

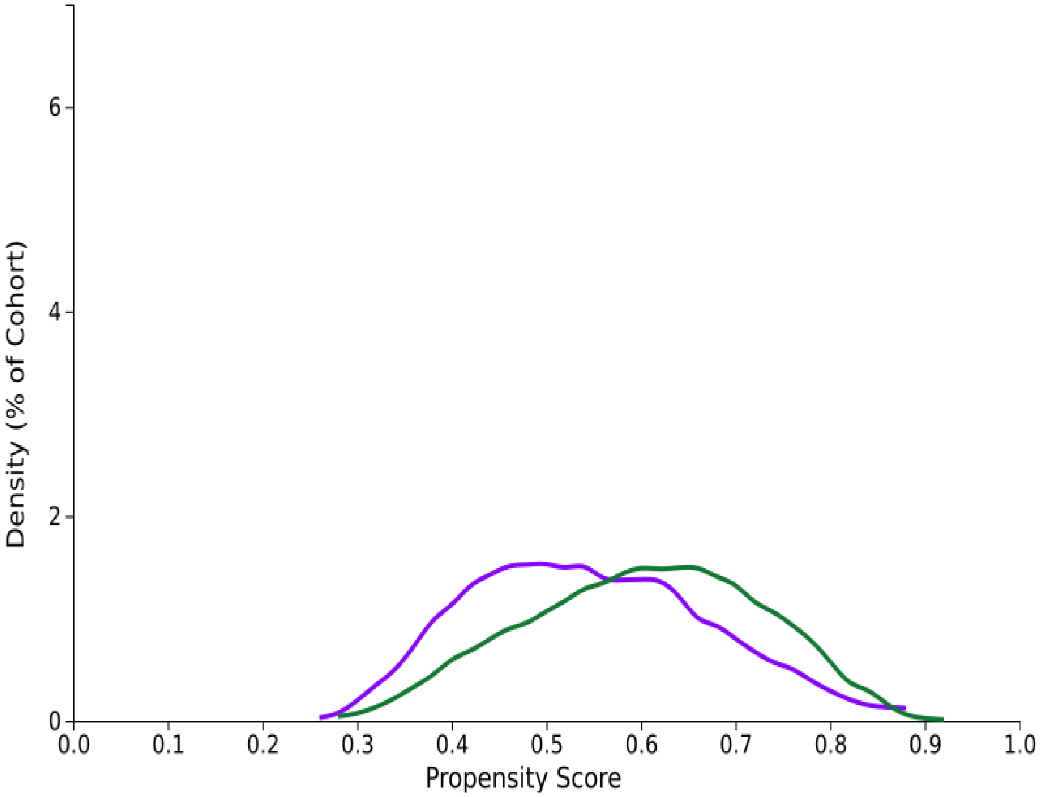

B. After matching

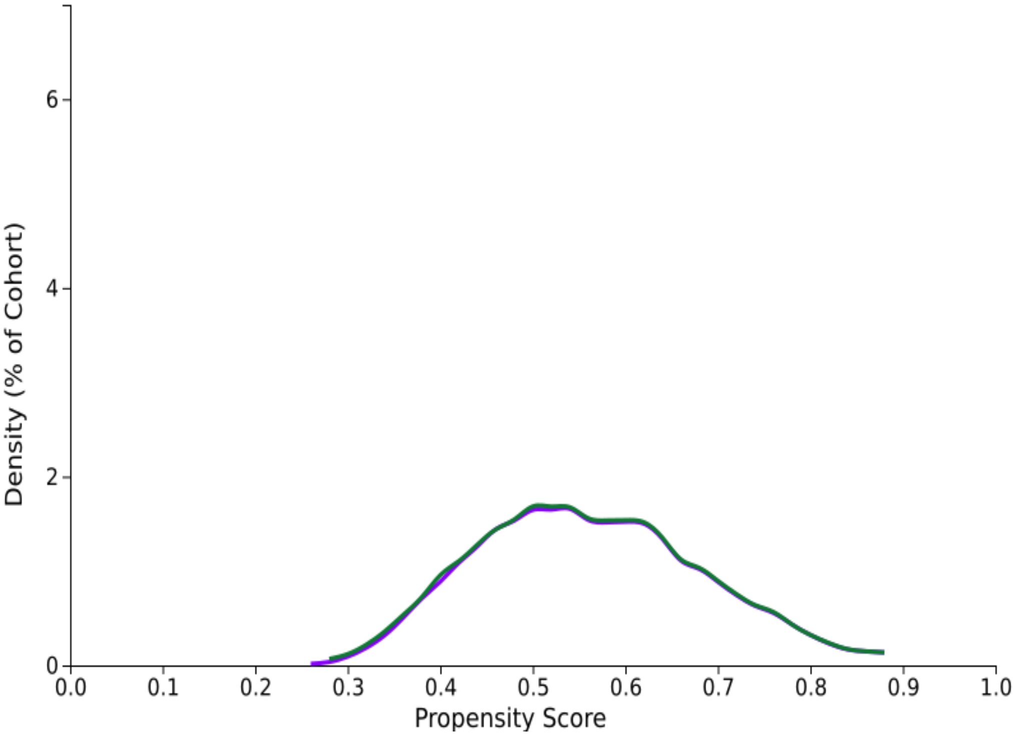

Figure 2. Forest plot of all outcomes in midodrine and non-midodrine groups in male (A) and female (B).

A. Male group

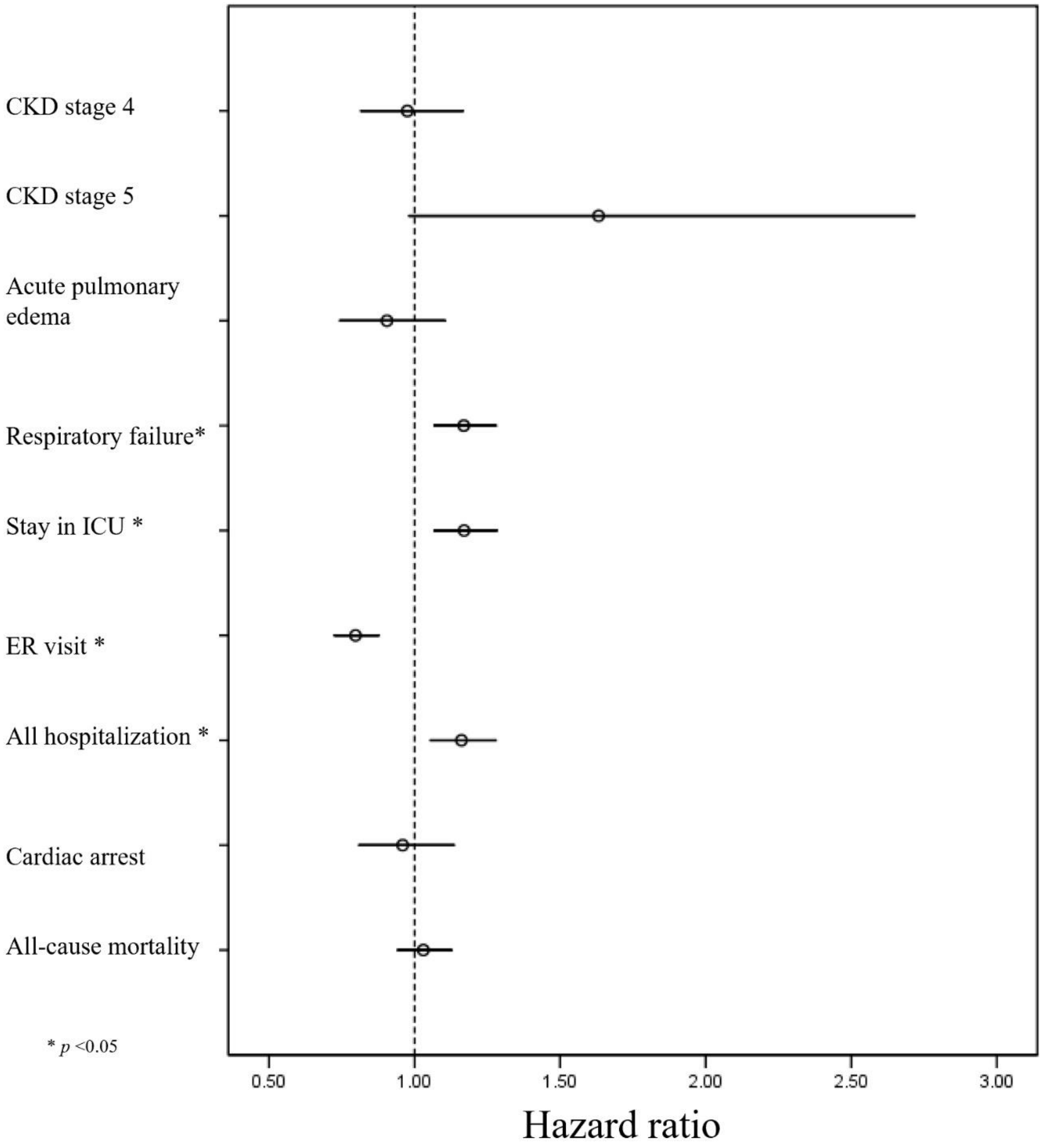

B Female group

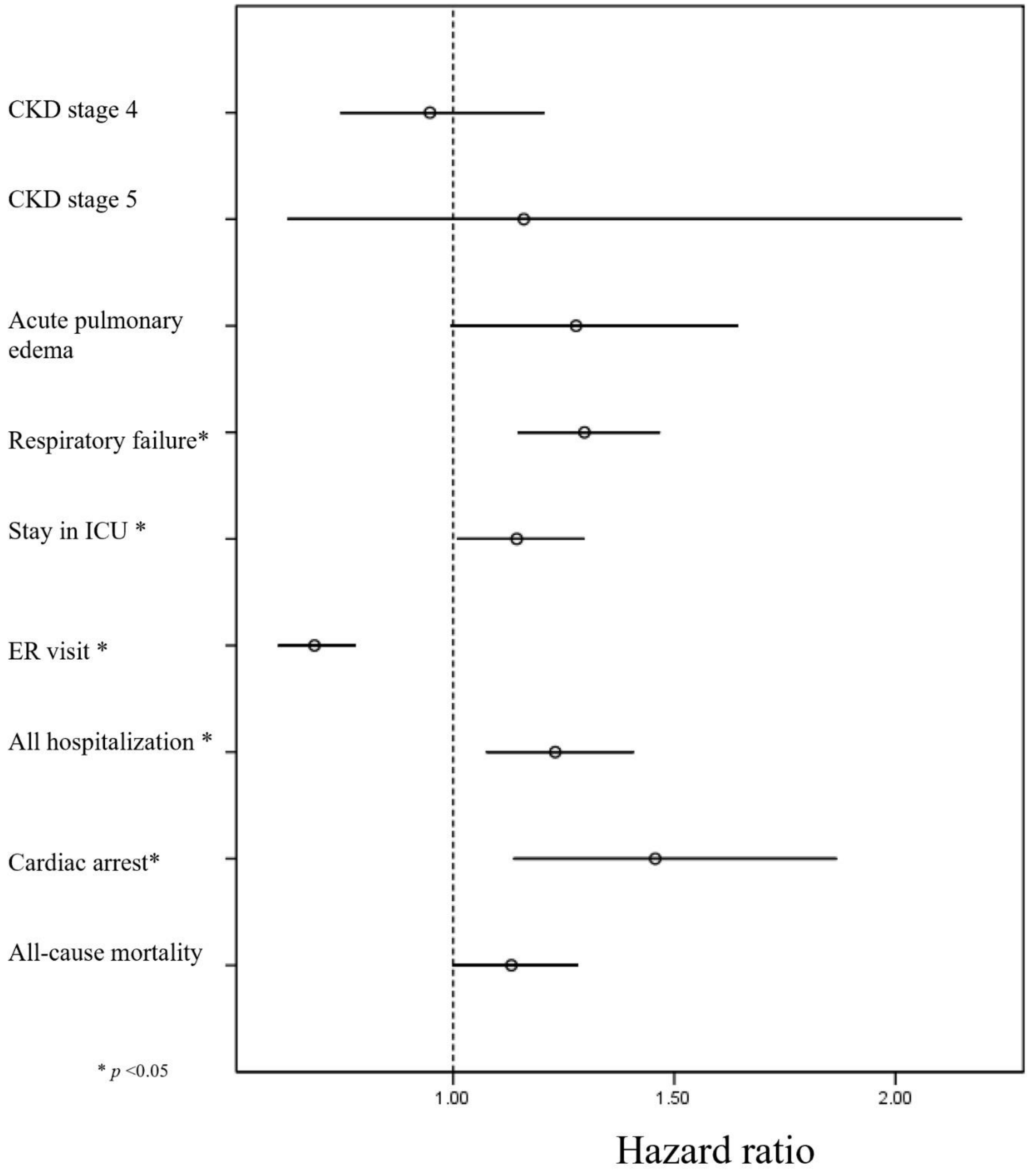

Figure 3. Forest plot of all outcomes in midodrine and non-midodrine groups in young (18-<65 y/o) (A) and elderly group (≥65 y/o) (B).

A. Young group

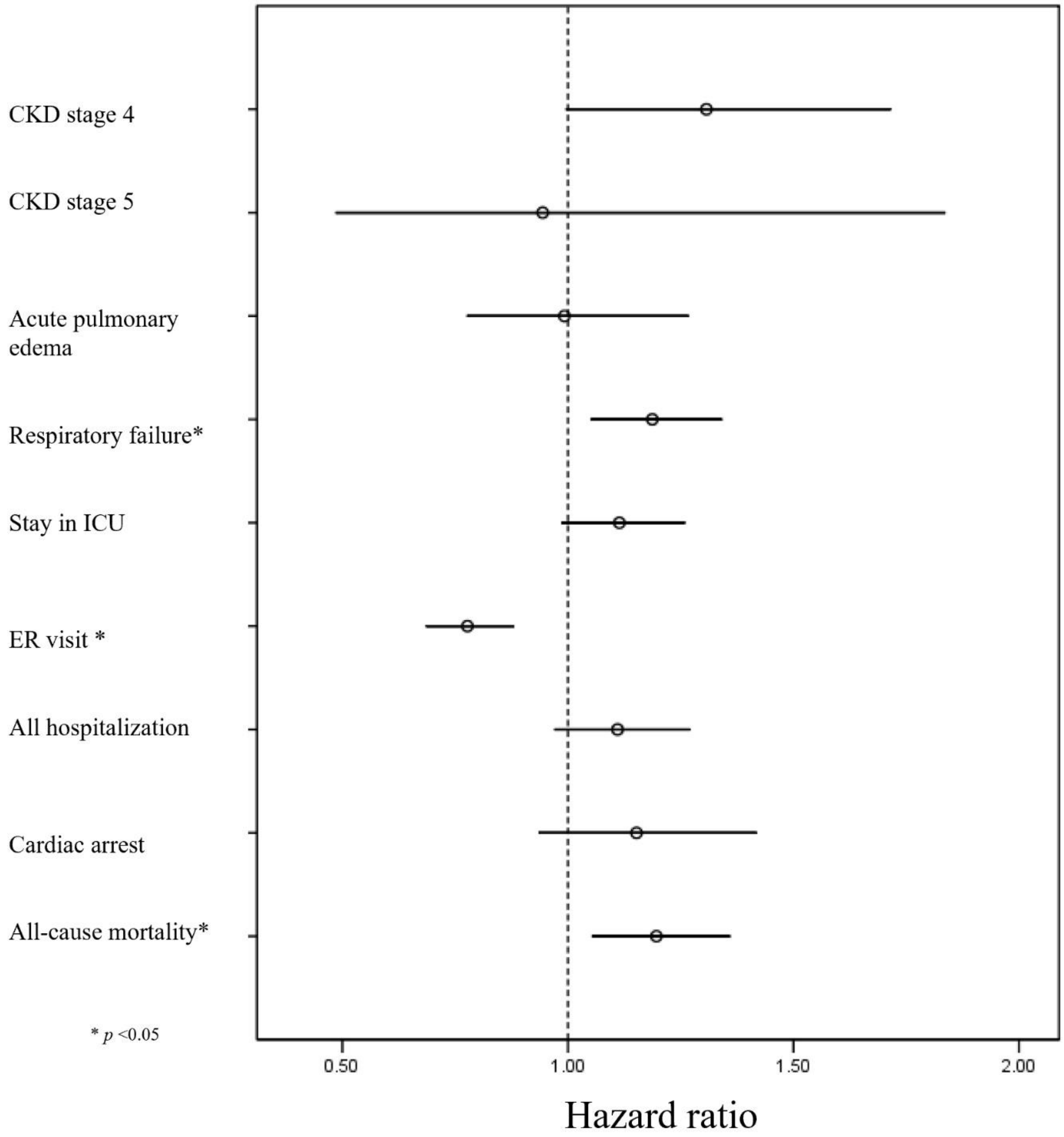

B. The elderly group

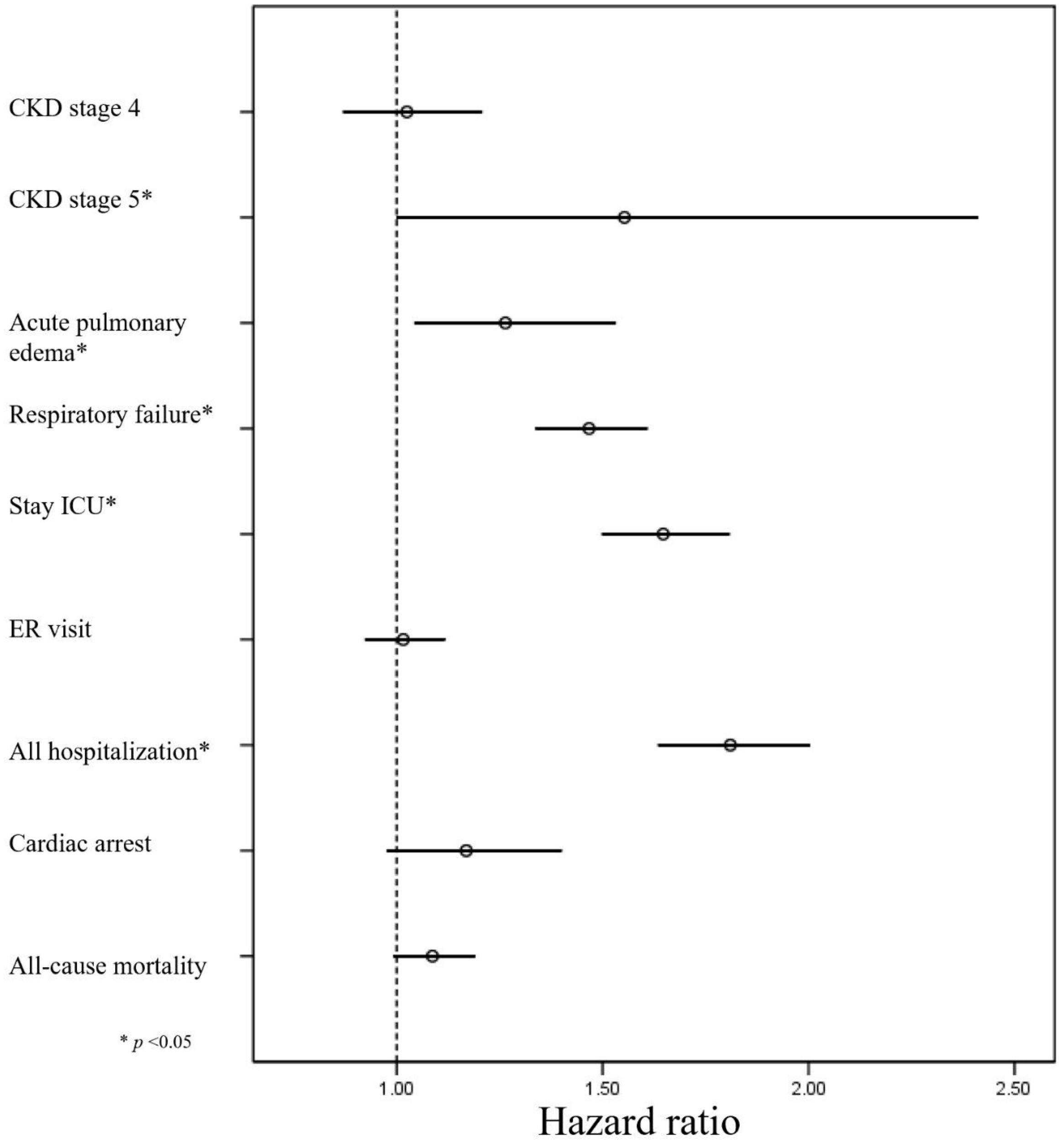

Figure 4. Forest plot of all outcomes in midodrine and non-midodrine groups in different groups according to times of prescription (at least a certain number of medication refills): ≥3 (A), ≥6 (B), ≥9 (C), and ≥12 (D)

A. 3 times at least

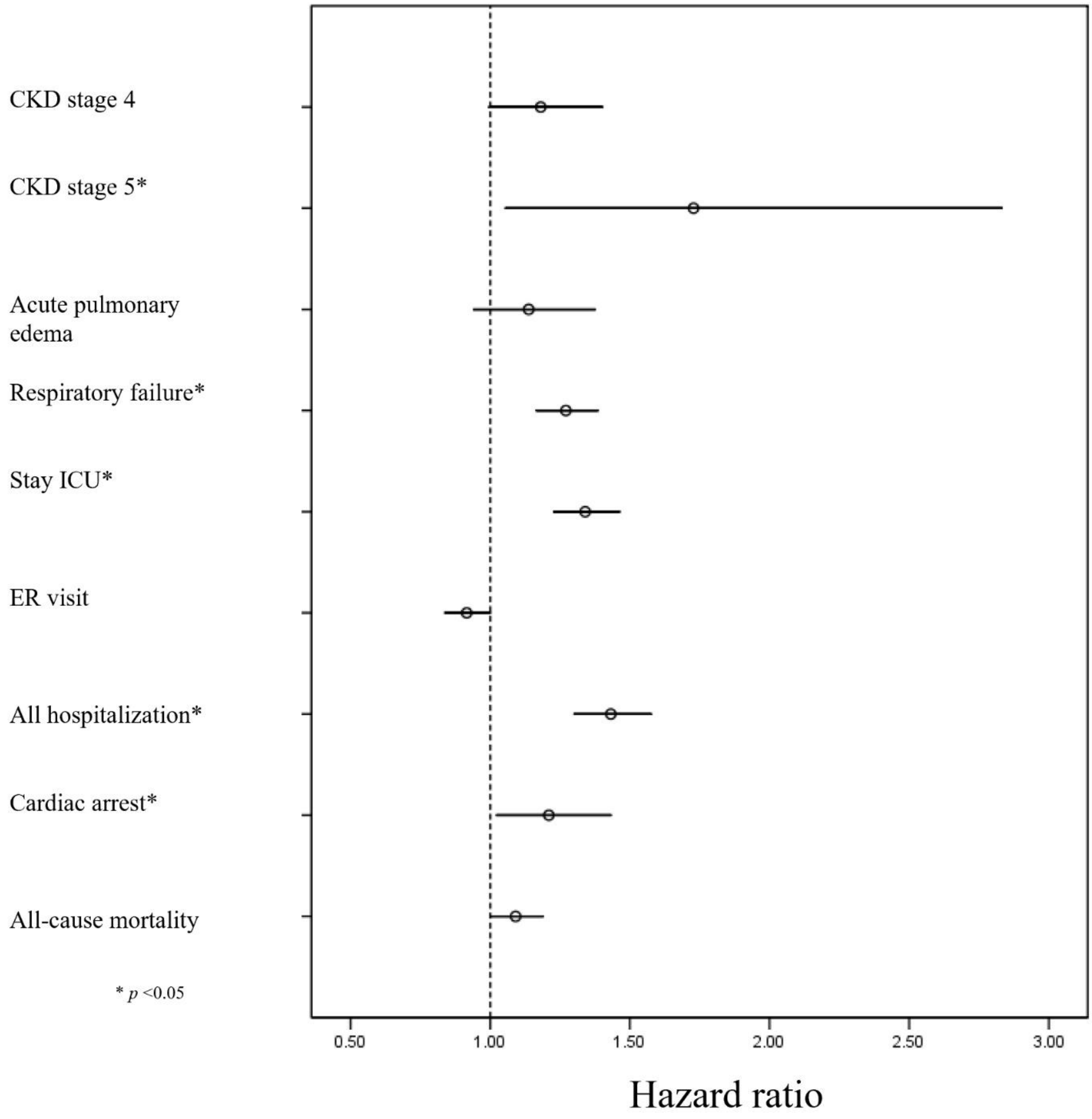

B. 6 times at least

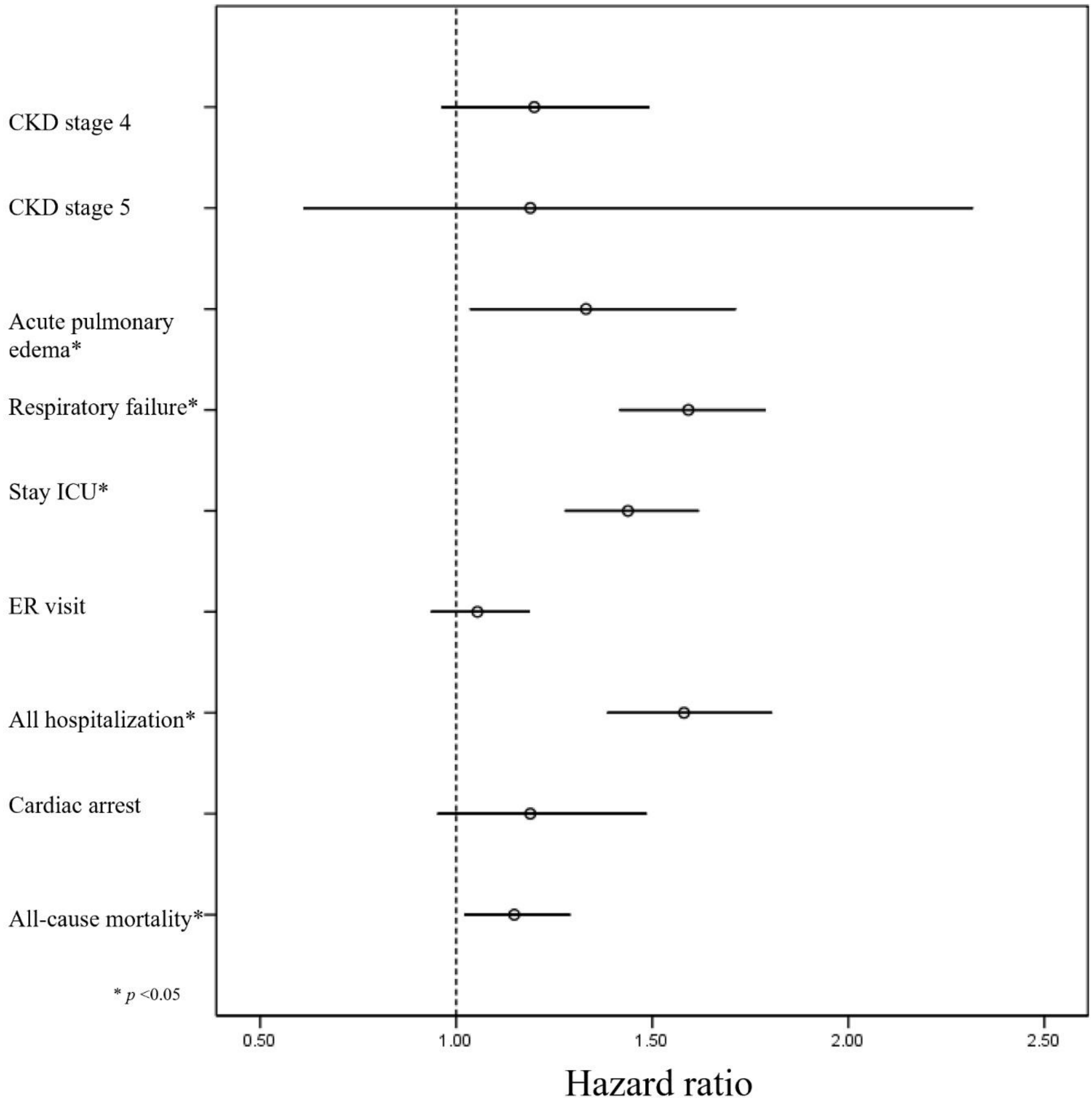

C. 9 times at least

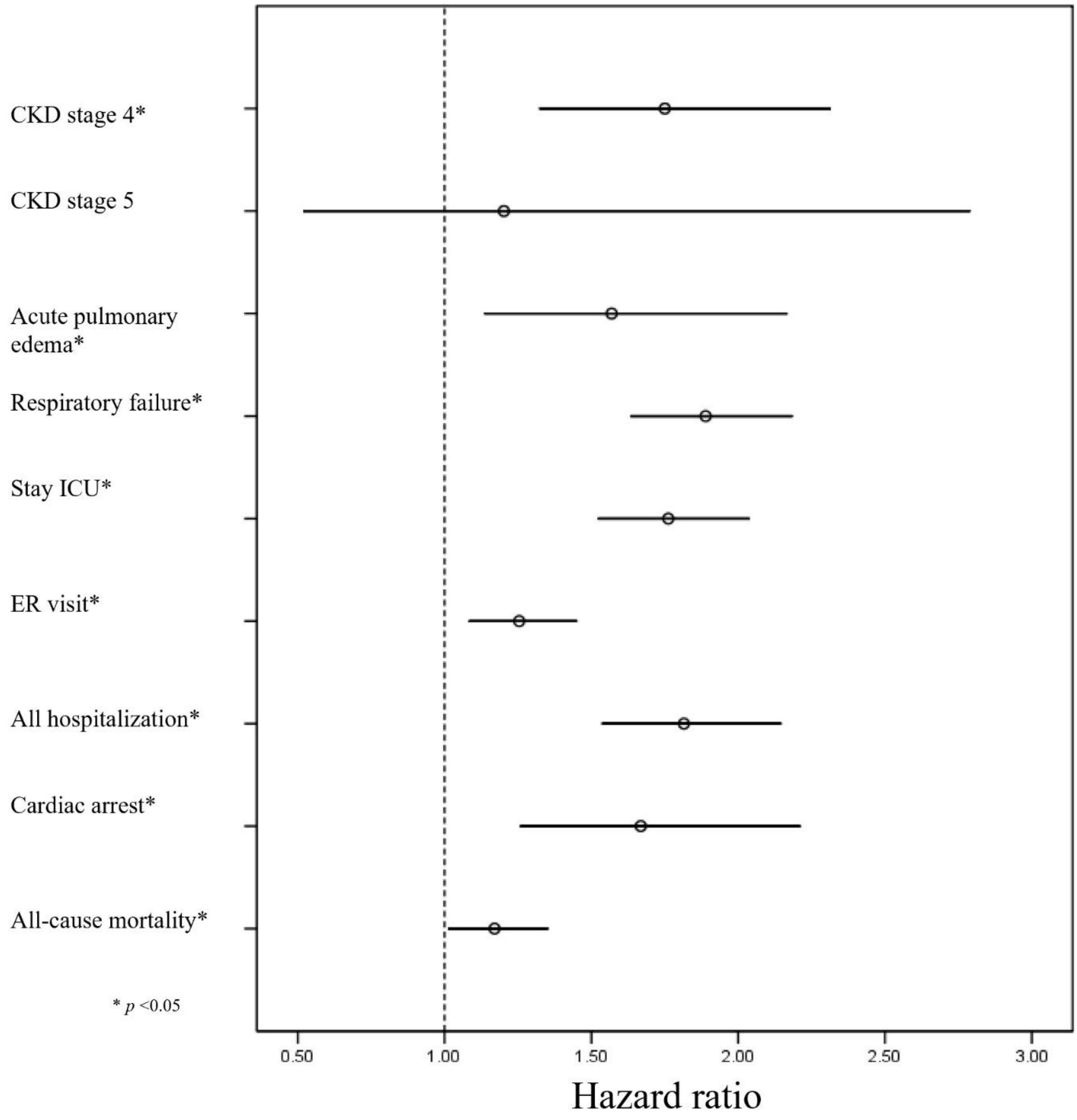

Figure 5. Forest plot of all outcomes in midodrine and non-midodrine groups in different groups according to baseline LVEF: 40-50% (A), 30-40%6 (B),20-30% (C), and <20% (D)

A. LVEF=40-50%

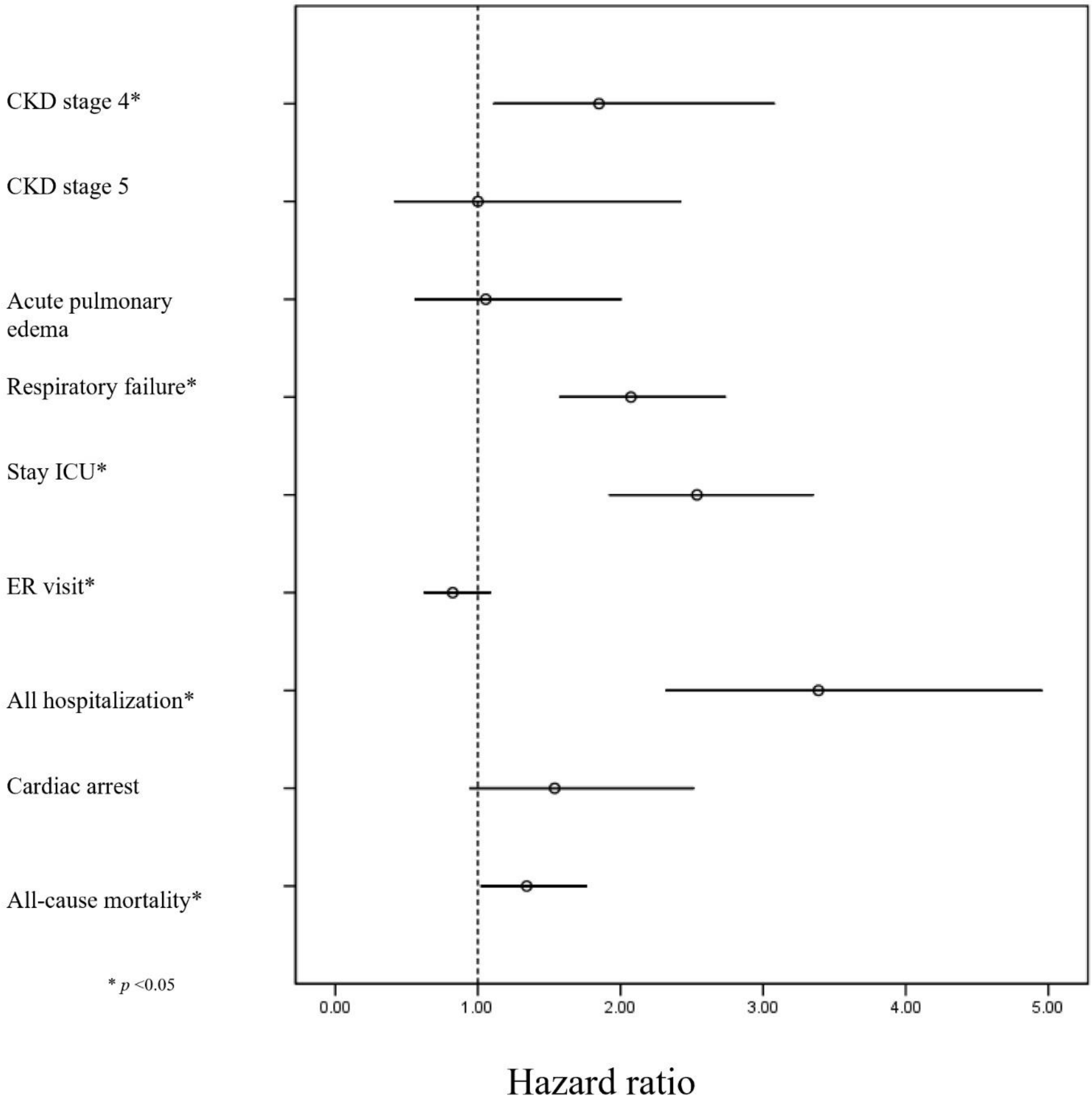

B. LVEF=30-40%

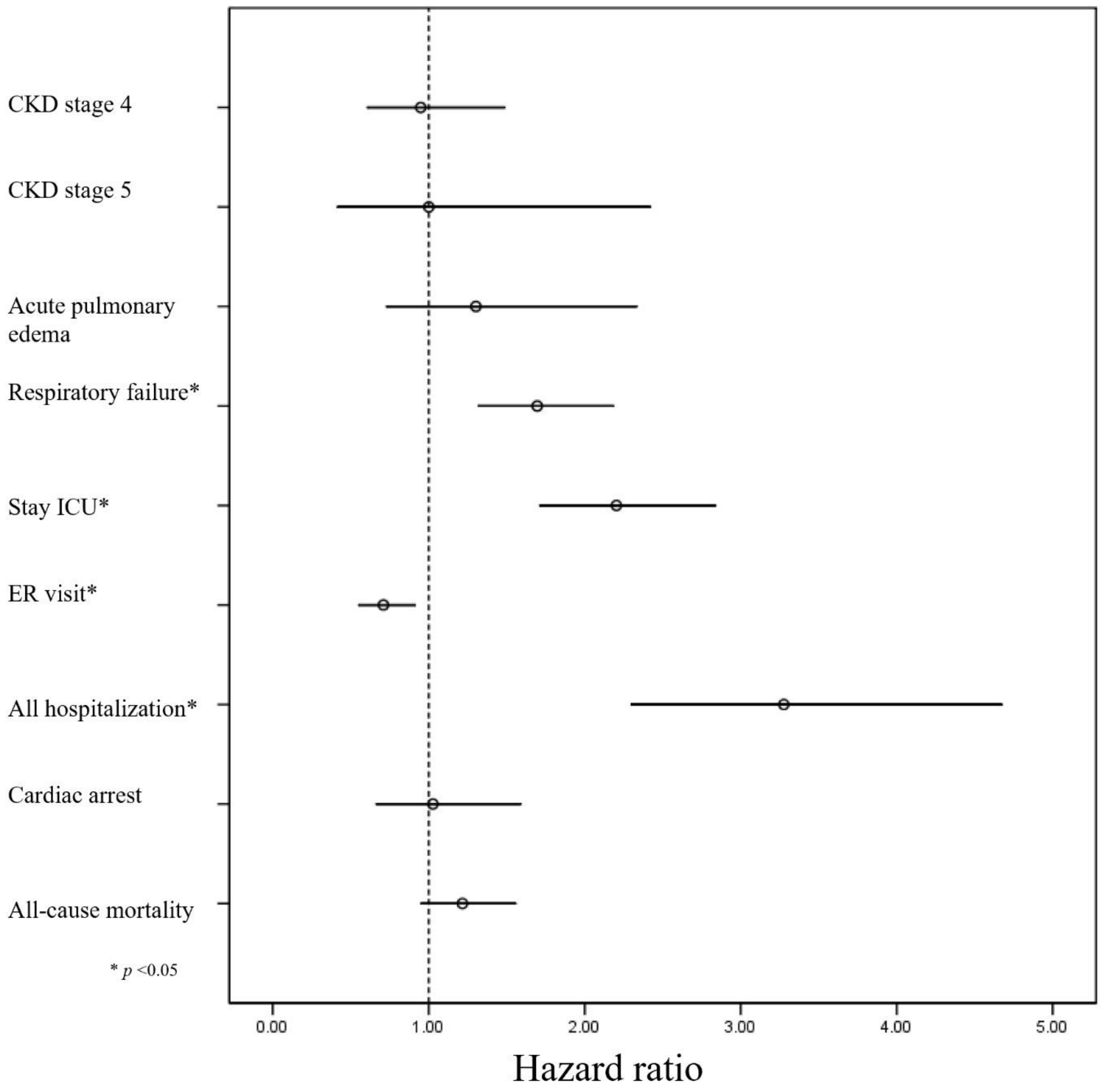
CKD stage 4
CKD stage 5
Acute pulmonary edema
Respiratory failure*
Stay ICU*
ER visit*
All hospitalization*
Cardiac arrest
All-cause mortality
* p <0.05
0.00
1.00
2.00
3.00
4.00
5.00
Hazard ratio

C. LVEF=20-30%

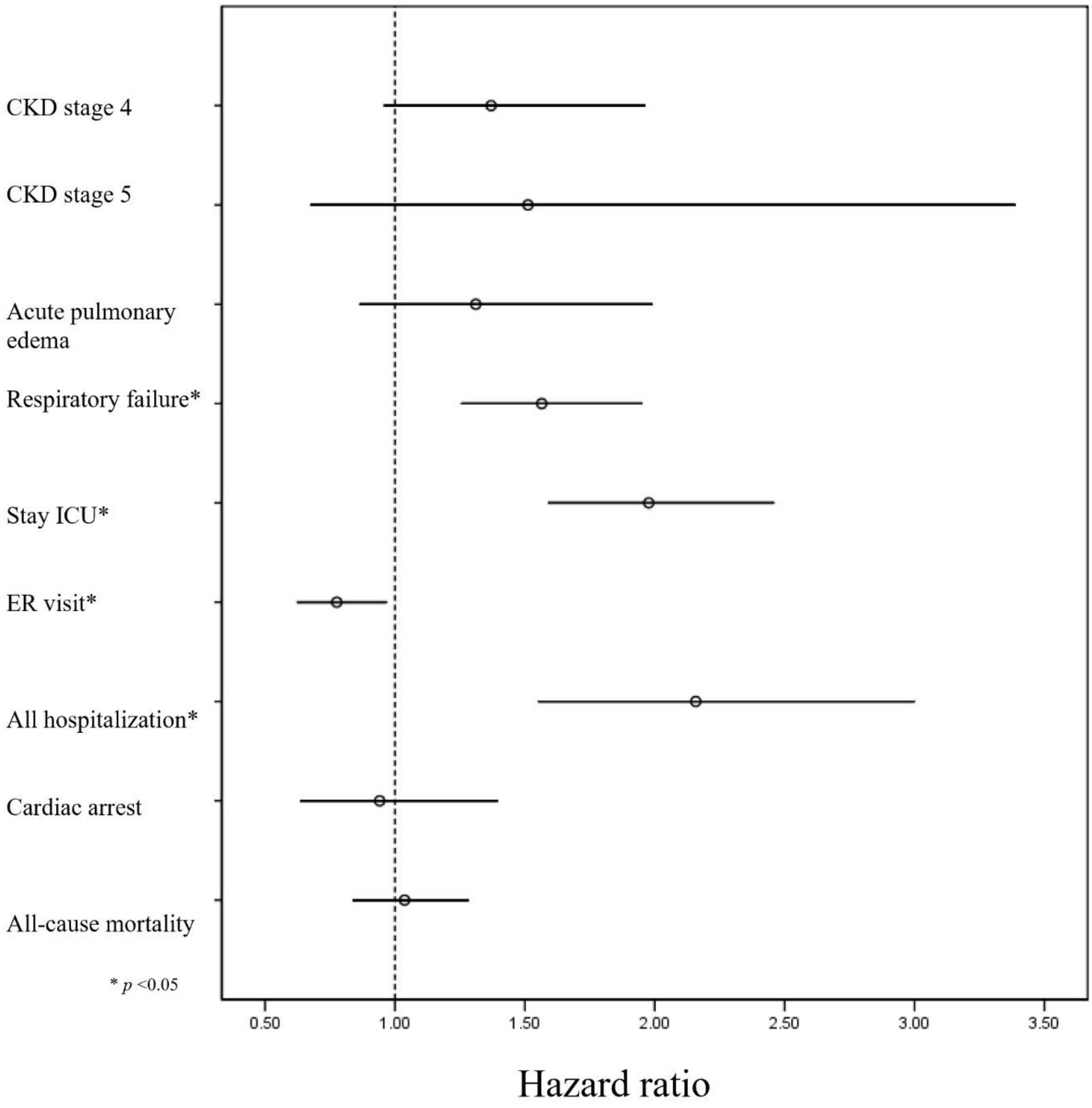
CKD stage 4
CKD stage 5
Acute pulmonary edema
Respiratory failure*
Stay ICU*
ER visit*
All hospitalization*
Cardiac arrest
All-cause mortality
* p <0.05
0.50
1.00
1.50
2.00
2.50
3.00
3.50
Hazard ratio

D. LVEF<20%

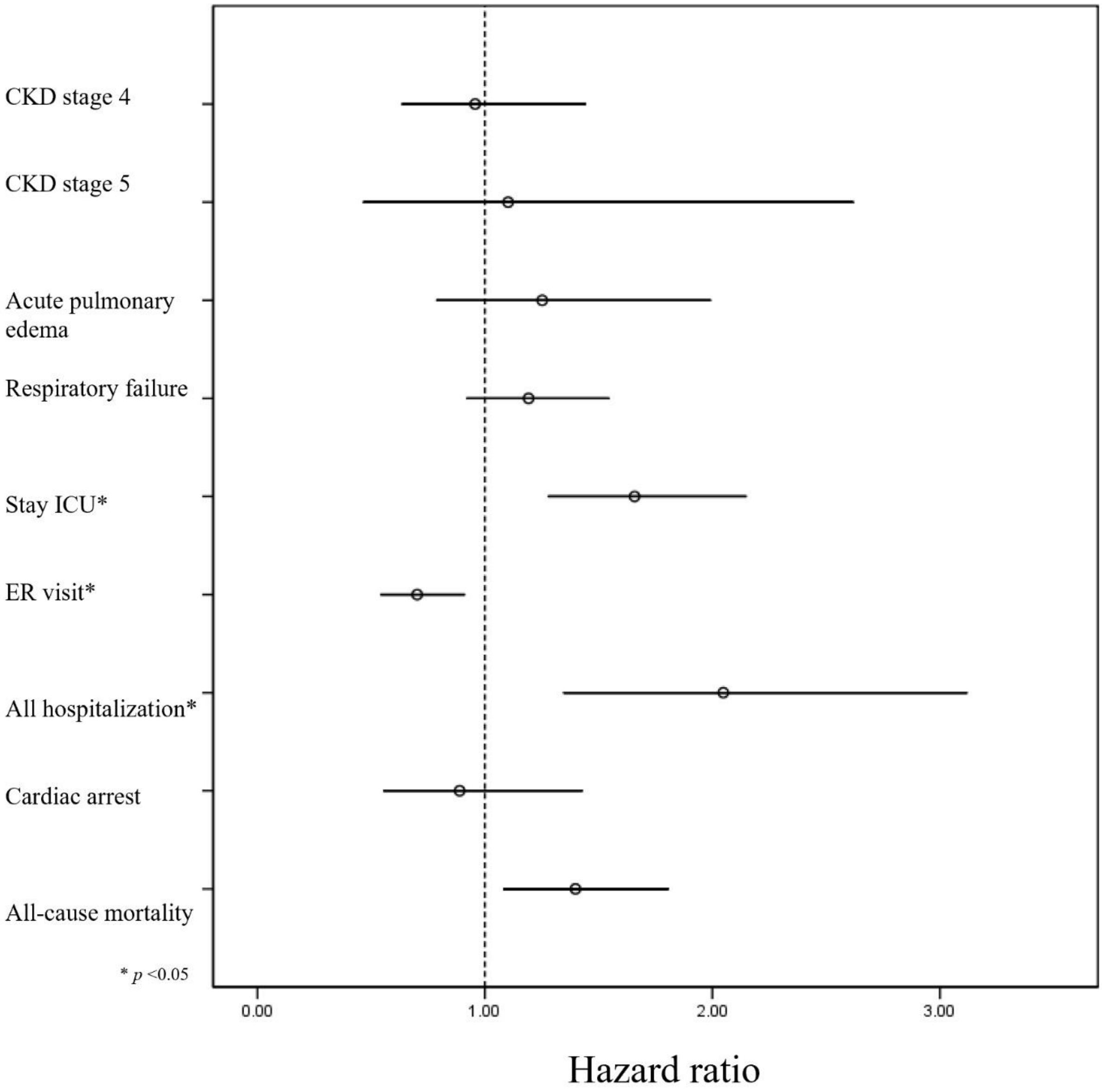

CKD stage 4
CKD stage 5
Acute pulmonary edema
Respiratory failure
Stay ICU*
ER visit*
All hospitalization*
Cardiac arrest
All-cause mortality
* p <0.05
0.00
1.00
2.00
3.00
Hazard ratio
